# Long-Term Once-Daily Tiotropium Respimat® Is Well Tolerated and Maintains Efficacy over 52 Weeks in Patients with Symptomatic Asthma in Japan: A Randomised, Placebo-Controlled Study

**DOI:** 10.1371/journal.pone.0124109

**Published:** 2015-04-20

**Authors:** Ken Ohta, Masakazu Ichinose, Yuji Tohda, Michael Engel, Petra Moroni-Zentgraf, Satoko Kunimitsu, Wataru Sakamoto, Mitsuru Adachi

**Affiliations:** 1 National Hospital Organization, Tokyo National Hospital, Tokyo, Japan; 2 Department of Respiratory Medicine, Tohoku University Graduate School of Medicine, Sendai, Japan; 3 Department of Respiratory Medicine and Allergology, Kinki University, Faculty of Medicine, Osakasayama City, Japan; 4 TA Respiratory Diseases, Boehringer Ingelheim Pharma GmbH & Co. KG, Ingelheim am Rhein, Germany; 5 Clinical Trial Management Department, Nippon Boehringer Ingelheim Co., Ltd., Tokyo, Japan; 6 Medical Data Services Department, Nippon Boehringer Ingelheim Co., Ltd., Tokyo, Japan; 7 Department of Clinical Research Center, International University of Health and Welfare, Sanno Hospital, Tokyo, Japan; University of California San Francisco, UNITED STATES

## Abstract

**Background:**

This study assessed the long-term safety and efficacy of tiotropium Respimat, a long-acting inhaled anticholinergic bronchodilator, in asthma, added on to inhaled corticosteroids (ICS) with or without long-acting β_2_-agonist (LABA).

**Methods:**

285 patients with symptomatic asthma, despite treatment with ICS±LABA, were randomised 2:2:1 to once-daily tiotropium 5 μg, tiotropium 2.5 μg or placebo for 52 weeks (via the Respimat SoftMist inhaler) added on to ICS±LABA, in a double-blind, placebo-controlled, parallel-group study (NCT01340209). Primary objective: to describe the long-term safety profile of tiotropium. Secondary end points included: trough forced expiratory volume in 1 second (FEV_1_) response; peak expiratory flow rate (PEFR) response; seven-question Asthma Control Questionnaire (ACQ-7) score.

**Results:**

At Week 52, adverse-event (AE) rates with tiotropium 5 μg, 2.5 μg and placebo were 88.6%, 86.8% and 89.5%, respectively. Commonly reported AEs with tiotropium 5 μg, 2.5 μg and placebo were nasopharyngitis (48.2%, 44.7%, 42.1%), asthma (28.9%, 29.8%, 38.6%), decreased PEFR (15.8%, 7.9%, 21.1%), bronchitis (9.6%, 13.2%, 7.0%), pharyngitis (7.9%, 13.2%, 3.5%) and gastroenteritis (10.5%, 3.5%, 5.3%). In the tiotropium 5 μg, 2.5 μg and placebo groups, 8.8%, 5.3% and 5.3% of patients reported drug-related AEs; 3.5%, 3.5% and 15.8% reported serious AEs. Asthma worsening was the only serious AE reported in more than one patient. At Week 52, adjusted mean trough FEV_1_ and trough PEFR responses were significantly higher with tiotropium 5 μg (but not 2.5 μg) versus placebo. ACQ-7 responder rates were higher with tiotropium 5 μg and 2.5 μg versus placebo at Week 24.

**Conclusions:**

The long-term tiotropium Respimat safety profile was comparable with that of placebo Respimat, and associated with mild to moderate, non-serious AEs in patients with symptomatic asthma despite ICS±LABA therapy. Compared with placebo, tiotropium 5 μg, but not 2.5 μg, significantly improved lung function and symptoms, supporting the long-term efficacy of the 5 μg dose.

**Trial Registration:**

ClinicalTrials.gov NCT01340209

## Introduction

Asthma is a chronic inflammatory disease affecting approximately 300 million people worldwide, and has a prevalence rate of around 7% in Japan [1,2]. Asthma treatment aims to reduce symptoms and exacerbation risk by treating the characteristic airway inflammation and bronchoconstriction, enabling patients to lead a normal and healthy life [1,3]. Inhaled corticosteroids (ICS) provide the cornerstone of asthma prophylactic treatment, supplemented by rapid-onset, short-acting bronchodilators for when fast symptomatic relief is required. Global Initiative for Asthma guidelines recommend a step-wise approach to treatment that relies on a continuous cycle of assessment, treatment and monitoring [1]. Low-dose ICS are recommended as the initial controller treatment. If symptoms are not adequately controlled, Global Initiative for Asthma guidelines recommend adding a long-acting β_2_-agonist (LABA), such as formoterol or salmeterol, before physicians consider increasing the dose of ICS or adding sustained-release theophylline or a leukotriene modifier. However, despite currently available therapies and detailed guidelines, at least 40% of patients with asthma have symptomatic or poorly controlled disease [4–6]. There remains a need to expand the therapeutic options available for patients whose asthma is not adequately controlled with ICS with or without a LABA.

Tiotropium is a once-daily long-acting anticholinergic bronchodilator currently approved for maintenance treatment in patients with chronic obstructive pulmonary disease (COPD). In Phase II and III placebo-controlled trials, tiotropium Respimat (Boehringer Ingelheim, Ingelheim am Rhein, Germany) add-on to high-dose ICS and LABA improved lung function versus placebo in patients who were still symptomatic despite treatment with ICS plus a LABA [7]. Tiotropium Respimat add-on also reduced the risk of asthma exacerbation and asthma worsening versus placebo [8]. Furthermore, the addition of tiotropium HandiHaler (Boehringer Ingelheim Pharmaceuticals, Inc., Ridgefield, Connecticut, USA) has been shown to be non-inferior to the addition of salmeterol [9,10] and superior to doubling the dose of ICS [10]. Tiotropium has demonstrated a favourable safety profile, with rates of adverse events (AEs) in patients with symptomatic asthma comparable with placebo when added to maintenance therapy in randomised clinical trials [7–10].

We conducted a Phase III, randomised, double-blind, placebo-controlled, parallel-group study to evaluate the long-term safety and efficacy of tiotropium Respimat (5 μg or 2.5 μg) compared with placebo Respimat in patients from Japan whose asthma was not being adequately controlled by ICS with or without a LABA.

## Methods

### Study design

The protocol for this trial and supporting CONSORT checklist are available as supporting information; see S1 CONSORT Checklist and S1 Protocol). In this Phase III, randomised, double-blind, placebo-controlled, parallel-group study (ClinicalTrials.gov identifier: NCT01340209; http://www.clinicaltrials.gov/ct2/show/NCT01340209?term=NCT01340209&rank=1), patients entered a 4-week screening period before being randomised to receive once-daily tiotropium 5 μg (two actuations of 2.5 μg), tiotropium 2.5 μg (two actuations of 1.25 μg) or placebo for 52 weeks, all delivered via the Respimat SoftMist inhaler (Boehringer Ingelheim) as add-on to ICS with or without a LABA. Patients were followed up for an additional 3 weeks following completion of the randomised treatment period. Patients were required to visit the trial centre every 4 weeks during the treatment period to record AEs and drug accountability. Lung function was assessed at initial screening, randomisation, Week 12, Week 24, Week 36 and Week 52 (at the end of treatment); these visits were conducted in the evening whereas all other visits could be conducted during the day. During the screening and treatment periods, patients used an electronic diary to record their asthma symptoms, quality of life, use of rescue medication and administration of the study medication. Patients who withdrew prematurely from the randomised treatment period continued to be followed up for their vital status.

The trial was carried out in compliance with the Declaration of Helsinki and in accordance with the International Conference on Harmonisation Harmonised Tripartite Guideline for Good Clinical Practice. Before initiation, the trial protocol, patient information sheet and consent form were reviewed and approved, according to national and international regulations, by the Japanese competent authority and the institutional review board of each participating centre: Obihiro Kokyukika Naika Hospital; Hitachi, Ltd. Hitachinaka General Hospital; Maebashi Hirosegawa Clinic; Shinagawa East One Medical Clinic; Sekino Hospital; Kimura Hospital; Urban Heights Clinic; Matsumoto Clinic; Nagoya City; Kyoto-Katsura Hospital; Hiroshima Allergy & Respiratory Clinic; Kyushu Central Hospital of the Mutual Aid Association of Public School Teachers; Kagoshima Seikyo Hospital; Makita Hospital; Tokushukai Group; National Hospital Organization Asahikawa Medical Center; Jimbo Orthopaedic; Kosei Clinic; Kameda Clinic; Sano Toranomon Clinic; Suzuki Internal Circulation; Meiwa Hospital; Tomei Atsugi Hospital; Mizuo Clinic; Yaesu Sakura-dori Clinic; Chubu Rosai Hospital; Social Insurance Chukyo Hospital; Japanese Red Cross Nagoya Daiichi Hospital; Keiseikai Yasuda Clinic; Yodogawa Christian Hospital; National Hospital Organization Himeji Medical Center; Matsue City Hospital; National Public Service Personnel Mutual Aid Associations, Kure Kyosai Hospital; Ehime University Hospital; National Hospital Organization Kochi National Hospital; K-you Health Care Co. Kirigaoka Tsuda Hospital; Iizuka Hospital; Saga University Hospital; Tokyo-Eki Center-Building Clinic; AMC Nishi-Umeda Clinic; Nakatani Hospital; Oita-chuo. Prior to participation in the trial, written, informed consent was required from each patient.

### Participants

Outpatients with symptomatic asthma lasting 12 weeks or more, and who were aged 18–75 years, were eligible for enrolment. The initial diagnosis of asthma must have been made before the age of 40 years, and was confirmed at screening with a bronchodilator reversibility (15–30 minutes after 400 μg salbutamol) resulting in a forced expiratory volume in 1 second (FEV_1_) increase of ≥12% and ≥200 mL. Patients were required to have been receiving maintenance treatment with stable medium-dose ICS (eg ≥400 μg and ≤800 μg budesonide or equipotent dose), alone or in a fixed combination with a LABA, for at least 4 weeks prior to screening. Symptomatic asthma was defined as a seven-question Asthma Control Questionnaire (ACQ-7) score of ≥1.5 at screening and prior to randomisation. All patients must also have had pre-bronchodilator FEV_1_ ≥60% and ≤90% of predicted normal, and to have never smoked or be ex-smokers who stopped smoking at least 1 year prior to enrolment, with a smoking history of less than 10 pack-years.

The main exclusion criteria were COPD and other significant unstable concomitant disease. Patients were not permitted to take any investigational drug, non-topical beta-blockers or other asthma therapies within 4 weeks prior to enrolment and/or during the screening period, or anti-immunoglobulin E antibodies within 6 months of enrolment and/or during the screening period.

Patients were also excluded if they: had any asthma exacerbation or any respiratory tract infection within 4 weeks prior to enrolment and/or during the screening period; were participating in another trial; had narrow-angle glaucoma and/or micturition disorder because of prostatic hyperplasia; and had failed to complete ≥80% of their electronic diary during the run-in period.

### Randomisation and treatment

Eligible patients were randomised in blocks, 2:2:1, to once-daily add-on tiotropium Respimat 5 μg, tiotropium Respimat 2.5 μg or placebo Respimat for 52 weeks. The 2:2:1 randomisation ratio was employed due to ethical considerations, as it would reduce the number of patients receiving placebo Respimat. Each patient received a single randomisation number indicating their allocated treatment. Randomisation was achieved via a third-party phone- or web-based system involving a validated pseudo-random number generator and a supplied seed number, using a block size of 5. All patients, including those randomised to the placebo Respimat group, continued to receive background medium-dose ICS with or without a LABA.

Study medication was taken each evening when patients inhaled two actuations using the Respimat SoftMist inhaler. In order to maintain the blind, patients in the placebo group also used the Respimat SoftMist inhaler, and the placebo inhalation solution was identical in appearance to the tiotropium inhalation solution. Blinding was maintained until after database lock.

Administration of rescue medication was allowed at any point during the trial. Open-label salbutamol hydrofluoroalkane metered-dose inhaler (100 μg per actuation) was provided as rescue medication for use as needed. Formoterol, alone or in fixed combinations with ICS, was not allowed as rescue medication in this trial. Temporary addition of systemic corticosteroids and theophylline preparations, temporary increases in the dose of ICS and the use of antibiotics were also allowed, at the discretion of the investigator, to control acute asthma exacerbations.

### Study end points

The primary objective of this trial was to evaluate the long-term safety of two doses (5 μg and 2.5 μg) of tiotropium Respimat, compared with placebo Respimat, as add-on to maintenance therapy with medium-dose ICS with or without a LABA, in patients with symptomatic asthma that was not adequately controlled at baseline. Safety was assessed by recorded AEs, vital signs (pulse rate and blood pressure), laboratory data and electrocardiogram.

Secondary efficacy end points included: trough FEV_1_ and trough forced vital capacity responses; in-clinic trough peak expiratory flow rate (PEFR) response using a spirometer; and ACQ-7 responder rate (minimal important difference of 0.5). Lung function responses were defined as change from baseline to the stated week; baseline was the pre-treatment value recorded at the randomisation visit 10 minutes before the evening dose of the patient’s usual ICS controller medication and first dose of study medication.

### Statistical analyses

The sample size was determined with reference to the International Conference on Harmonisation of Technical Requirements for Registration of Pharmaceuticals for Human Use E1 guideline on population exposure, to assess long-term clinical safety and to satisfy Japanese regulatory authority requirements. At least 100 patients were required to remain on treatment for 52 weeks to describe the safety profile of tiotropium Respimat 5 μg and 2.5 μg. Assuming a drop-out rate of 10%, it was calculated that 280 patients (112 patients per tiotropium Respimat group and 56 patients in the placebo Respimat group) should be enrolled.

The summary of safety data was based on the treated set (all randomised patients who received one or more doses of study medication) and was evaluated using descriptive analysis. The study was not designed to detect any difference between the two doses of tiotropium Respimat and placebo Respimat in terms of overall or any specific AE incidence. The placebo Respimat group was included to obtain information on the safety profile of the placebo-control group (on a background of ICS with or without a LABA) and to reduce any possible reporting bias for drug-related AEs.

The analysis of efficacy data was performed on the full analysis set (all treated patients with baseline data and at least one on-treatment efficacy measurement). Power calculations for these secondary analyses were not conducted, as the primary objective of the study was to assess the safety profile of tiotropium Respimat. Lung function tests were evaluated using a restricted maximum likelihood-based mixed effects model with repeated measures. Analyses included the fixed, categorical effects of ‘treatment’, ‘visit’ and ‘treatment-by-visit interaction’, as well as the continuous, fixed covariates of ‘baseline value’ and ‘baseline value-by-visit interaction’. ‘Patient’ was included as random effect. Analyses were performed on least squares means using a two-sided α = 0.05 (two-sided 95% confidence intervals [CIs]). As it was not planned to test any statistical hypothesis in a confirmatory manner, all p values were nominally interpreted and no correction for multiple testing was applied. Other end points were summarised using descriptive statistics.

## Results

### Patient disposition and demographics

**Fig 1:**
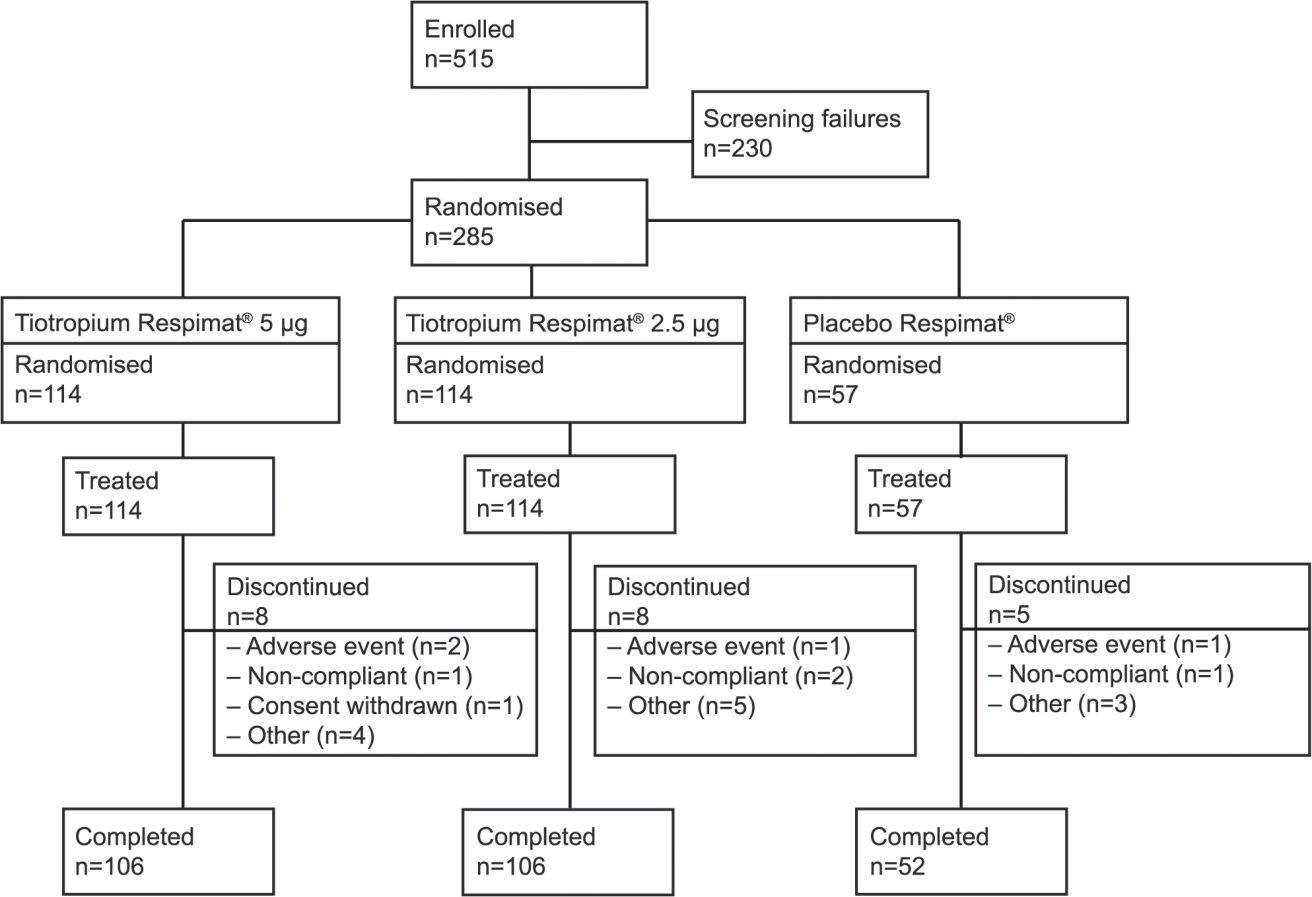
CONSORT diagram.

Between April 2011 and April 2013, 515 patients were enrolled from 54 centres in Japan. Following screening, 285 patients were randomised to treatment. As per the study protocol, twice as many patients were randomised to each of the tiotropium Respimat groups compared with the placebo Respimat group. Overall, 21 patients (7.4%) prematurely discontinued trial medication and 264 patients completed the study (Fig 1).

Baseline demographic characteristics were generally well balanced across the three treatment groups (Table 1). The majority of patients were female (61.8%), mean age was 44.5 years and mean body mass index at baseline was 24.4 kg/m^2^. Most patients had never smoked (75.1%); one patient (1.8%) in the placebo Respimat group was a current smoker at screening, which was in violation of the protocol. In terms of lung function at baseline, mean (pre-dose) FEV_1_ was 2.28 L and mean percent of predicted FEV_1_ was 80.2%. Mean ACQ-7 total score at baseline was 1.95, and at the screening visit mean FEV_1_ reversibility was 23%. Overall, mean duration of asthma was 22.4 years (median duration of asthma, 22.0 years) and mean age at onset of asthma was 22.1 years (median age, 24.8 years). Concomitant LABA was used by 57.0% of patients in the tiotropium Respimat 5 μg group, 54.4% of patients in the tiotropium Respimat 2.5 μg group and 61.4% of patients in the placebo Respimat group. Overall, the mean ICS dose of stable maintenance therapy (budesonide equipotent dose) at baseline was 661.7 μg and the median ICS dose was 800 μg (range 400–1600 μg); doses were comparable across the three treatment groups.

**Table 1:** Baseline patient and disease characteristics.

	Tiotropium Respimat 5 μg (n = 114)^a^	Tiotropium Respimat 2.5 μg (n = 114) ^a^	Placebo Respimat (n = 57) ^a^
Sex, n (%)			
Male	48 (42.1)	42 (36.8)	19 (33.3)
Female	66 (57.9)	72 (63.2)	38 (66.7)
Age, years	42.6 (12.8)	44.7 (12.1)	47.8 (13.0)
Weight, kg	64.1 (12.8)	64.6 (17.1)	61.5 (13.6)
Height, cm	162.6 (8.4)	161.0 (8.8)	159.6 (8.6)
Body mass index, kg/m^2^	24.2 (4.1)	24.8 (5.6)	24.0 (4.1)
Smoking status			
Never smoked, n (%)	87 (76.3)	88 (77.2)	39 (68.4)
Ex-smoker, n (%)	27 (23.7)	26 (22.8)	17 (29.8)
Current smoker, n (%)	0	0	1 (1.8)
Smoking history, pack-years^b^	4.51 (2.71)	4.25 (2.31)	5.33 (4.69)
Concomitant treatment, n (%)			
ICS	114 (100)	114 (100)	57 (100)
Long-acting β_2_-agonists	65 (57.0)	62 (54.4)	35 (61.4)
Short-acting β-agonists	1 (0.9)	7 (6.1)	2 (3.5)
Leukotriene modifiers	29 (25.4)	36 (31.6)	14 (24.6)
Theophyllines	19 (16.7)	26 (22.8)	10 (17.5)
Systemic antihistamines	20 (17.5)	23 (20.2)	7 (12.3)
Omalizumab	0	0	0
ICS maintenance dose, μg^c^	658.9 (220.5)	673.2 (247.4)	644.2 (220.9)
Median (range) age at asthma onset, years	23.9 (1–39)	25.0 (1–40)	25.7 (1–40)
Median (range) duration of asthma, years	21.0 (0.3–54.0)	21.0 (0.8–57.8)	26.8 (0.8–63.0)
ACQ-7 total score	1.97 (0.38)	1.94 (0.43)	1.90 (0.32)
FEV_1_ reversibility at screening, % of pre-bronchodilator	23.5 (12.5)	22.4 (10.8)	23.0 (10.6)

Values are mean (standard deviation) unless otherwise stated.

^a^Patients were randomised 2:2:1 to the tiotropium Respimat 5 μg, tiotropium Respimat 2.5 μg and placebo Respimat groups, respectively.

^b^Calculated in smokers and ex-smokers.

^c^Budesonide equipotent dose.

ACQ-7, seven-question Asthma Control Questionnaire; FEV_1_, forced expiratory volume in 1 second; ICS, inhaled corticosteroids.

### Long-term safety

All randomised patients received one or more doses of trial medication and were therefore included in the treated set for long-term safety analysis. The median exposure (ie duration of treatment) was 365 days in all three treatment groups, and 93.0% of patients had ≥364 days of exposure. Median treatment compliance was 93.7%.

The incidence of AEs was similar across treatment groups; AEs were reported in 101/114 (88.6%), 99/114 (86.8%) and 51/57 (89.5%) patients in the tiotropium Respimat 5 μg, tiotropium Respimat 2.5 μg and placebo Respimat groups, respectively (Table 2). Most AEs were mild to moderate in intensity. Drug-related AEs were reported in 10 (8.8%), six (5.3%) and three (5.3%) patients in the tiotropium Respimat 5 μg, tiotropium Respimat 2.5 μg and placebo Respimat groups, respectively.

**Table 2:** Overall summary of adverse events (treated set).

n (%)	Tiotropium Respimat 5 μg (n = 114)^a^	Tiotropium Respimat 2.5 μg (n = 114)^a^	Placebo Respimat (n = 57)^a^
Any AE	101 (88.6)	99 (86.8)	51 (89.5)
Severe AEs	2 (1.8)	1 (0.9)	3 (5.3)
Drug-related AEs^b^	10 (8.8)	6 (5.3)	3 (5.3)
AEs leading to discontinuation	2 (1.8)	1 (0.9)	1 (1.8)
Significant (pre-specified) AEs^c^	0	0	0
Serious AEs	4 (3.5)	4 (3.5)	9 (15.8)
Requiring hospitalisation	4 (3.5)	4 (3.5)	7 (12.3)
Drug-related	0	0	1 (1.8)^d^
Fatal	0	0	0
Other	0	0	2 (3.5)

^a^Patients were randomised 2:2:1 to the tiotropium Respimat 5 μg, tiotropium Respimat 2.5 μg and placebo Respimat groups, respectively.

^b^As determined by the investigator.

^c^Elevation of aspartate aminotransferase and/or alanine aminotransferase ≥3 × upper limit of normal combined with elevated total bilirubin ≥2 × upper limit of normal at the same visit.

^d^Asthma worsening.

AE, adverse event.

The most commonly reported AEs were nasopharyngitis, decreased PEFR, asthma worsening, bronchitis, pharyngitis and gastroenteritis (Table 3). A higher proportion of patients reported bronchitis in the tiotropium Respimat groups than in the placebo Respimat group: tiotropium 5 μg, 11 (9.6%); tiotropium 2.5 μg, 15 (13.2%); placebo, four (7.0%). Conversely, a lower proportion of patients reported asthma worsening in the tiotropium Respimat groups than in the placebo Respimat group: tiotropium Respimat 5 μg, 33 (28.9%); tiotropium Respimat 2.5 μg, 34 (29.8%); placebo Respimat, 22 (38.6%).

**Table 3:** Frequency of adverse events with incidence ≥4% in any treatment group (treated set).

n (%)	Tiotropium Respimat 5 μg (n = 114)^a^	Tiotropium Respimat 2.5 μg (n = 114)^a^	Placebo Respimat (n = 57) ^a^
Infections and infestations	81 (71.1)	81 (71.1)	33 (57.9)
Nasopharyngitis	55 (48.2)	51 (44.7)	24 (42.1)
Bronchitis	11 (9.6)	15 (13.2)	4 (7.0)
Pharyngitis	9 (7.9)	15 (13.2)	2 (3.5)
Gastroenteritis	12 (10.5)	4 (3.5)	3 (5.3)
Upper respiratory tract infection	5 (4.4)	8 (7.0)	2 (3.5)
Influenza	5 (4.4)	7 (6.1)	2 (3.5)
Cystitis	5 (4.4)	3 (2.6)	1 (1.8)
Respiratory, thoracic and mediastinal disorders	47 (41.2)	46 (40.4)	28 (49.1)
Asthma worsening^b^	33 (28.9)	34 (29.8)	22 (38.6)
Upper respiratory tract inflammation	7 (6.1)	7 (6.1)	4 (7.0)
Dysphonia	7 (6.1)	2 (1.8)	0
Allergic rhinitis	2 (1.8)	3 (2.6)	4 (7.0)
Investigations	23 (20.2)	12 (10.5)	16 (28.1)
Decreased peak expiratory flow rate	18 (15.8)	9 (7.9)	12 (21.1)
Gastrointestinal disorders	26 (22.8)	18 (15.8)	13 (22.8)
Gastritis	6 (5.3)	3 (2.6)	4 (7.0)
Constipation	1 (0.9)	0	3 (5.3)
Gastro-oesophageal reflux disease	6 (5.3)	1 (0.9)	1 (1.8)
Diarrhoea	5 (4.4)	5 (4.4)	0
Musculoskeletal and connective tissue disorders	15 (13.2)	9 (7.9)	9 (15.8)
Back pain	4 (3.5)	4 (3.5)	3 (5.3)
Injury, poisoning and procedural complications	12 (10.5)	8 (7.0)	7 (12.3)
Ligament sprain	3 (2.6)	2 (1.8)	3 (5.3)
Nervous system disorders	10 (8.8)	12 (10.5)	3 (5.3)
Headache	4 (3.5)	7 (6.1)	1 (1.8)
Eye disorders	6 (5.3)	7 (6.1)	5 (8.8)
Allergic conjunctivitis	2 (1.8)	5 (4.4)	1 (1.8)
Vascular disorders	5 (4.4)	1 (0.9)	2 (3.5)
Hypertension	5 (4.4)	1 (0.9)	0

^a^Patients were randomised 2:2:1 to the tiotropium Respimat 5 μg, tiotropium Respimat 2.5 μg and placebo Respimat groups, respectively.

^b^The preferred term ‘asthma’ summarises several lowest level terms.

The type of AEs that were considered by the treating physician to be drug-related varied widely. Drug-related AEs reported in more than one patient were asthma worsening (tiotropium Respimat 5 μg, 1.8%; tiotropium Respimat 2.5 μg, 0%; placebo Respimat, 1.8%), thirst (tiotropium Respimat 5 μg, 1.8%; tiotropium Respimat 2.5 μg, 0.9%; placebo Respimat, 0%), dysphonia (tiotropium Respimat 5 μg, 1.8%; tiotropium Respimat 2.5 μg, 0%; placebo Respimat, 0%) and headache (tiotropium Respimat 5 μg, 0%; tiotropium Respimat 2.5 μg, 1.8%; placebo Respimat, 0%). All other drug-related AEs were single occurrences.

Nine cardiac disorder events were reported in five patients (4.4%) in the tiotropium Respimat 5 μg group, compared with one event in one patient (0.9%) in the tiotropium Respimat 2.5 μg group, and no patients in the placebo Respimat group (Table 4). Five cardiac AEs in the tiotropium Respimat 5 μg group occurred in the same patient at the same time, soon after the start of trial medication, and resolved shortly after discontinuation, which were therefore assessed by the investigator as being drug-related. These events (atrioventricular block [first degree], extrasystoles, palpitations, supraventricular extrasystoles and ventricular extrasystoles) were all mild in intensity and required no treatment. The same patient in the tiotropium Respimat 5 μg group also had hypertension that was considered to be drug-related.

**Table 4:** Frequency of cardiac adverse events (treated set).

n (%)	Tiotropium Respimat 5 μg (n = 114)^a^	Tiotropium Respimat 2.5 μg (n = 114) ^a^	Placebo Respimat (n = 57) ^a^
Any cardiac adverse event	5 (4.4)	1 (0.9)	0
Palpitations^b^	2 (1.8)	1 (0.9)	0
Ventricular extrasystoles^b^	2 (1.8)	0	0
Atrioventricular block, first degree^b^	1 (0.9)	0	0
Extrasystoles^b^	1 (0.9)	0	0
Prinzmetal’s angina	1 (0.9)	0	0
Supraventricular extrasystoles^b^	1 (0.9)	0	0
Tachycardia	1 (0.9)	0	0

^a^Patients were randomised 2:2:1 to the tiotropium Respimat 5 μg, tiotropium Respimat 2.5 μg and placebo Respimat groups, respectively.

^b^One event from each category occurred in a single patient prior to Day 59.

Serious AEs (SAEs) were less frequent with tiotropium Respimat 5 μg (n = 4; 3.5%) or tiotropium Respimat 2.5 μg (n = 4; 3.5%) than with placebo Respimat (n = 9; 15.8%). One SAE in each of the tiotropium Respimat 5 μg and placebo Respimat groups was coded as asthma worsening, all other SAEs were single occurrences, and only the asthma worsening event in the placebo Respimat group was considered to be drug-related (S1 Table). No deaths or life-threatening AEs were reported during the study.

### Long-term efficacy

The full analysis set for the long-term efficacy analysis comprised 114, 114 and 56 patients in the tiotropium Respimat 5 μg, tiotropium Respimat 2.5 μg and placebo Respimat groups, respectively, for whom baseline data and at least one on-treatment efficacy measurement were available.

**Fig 2:**
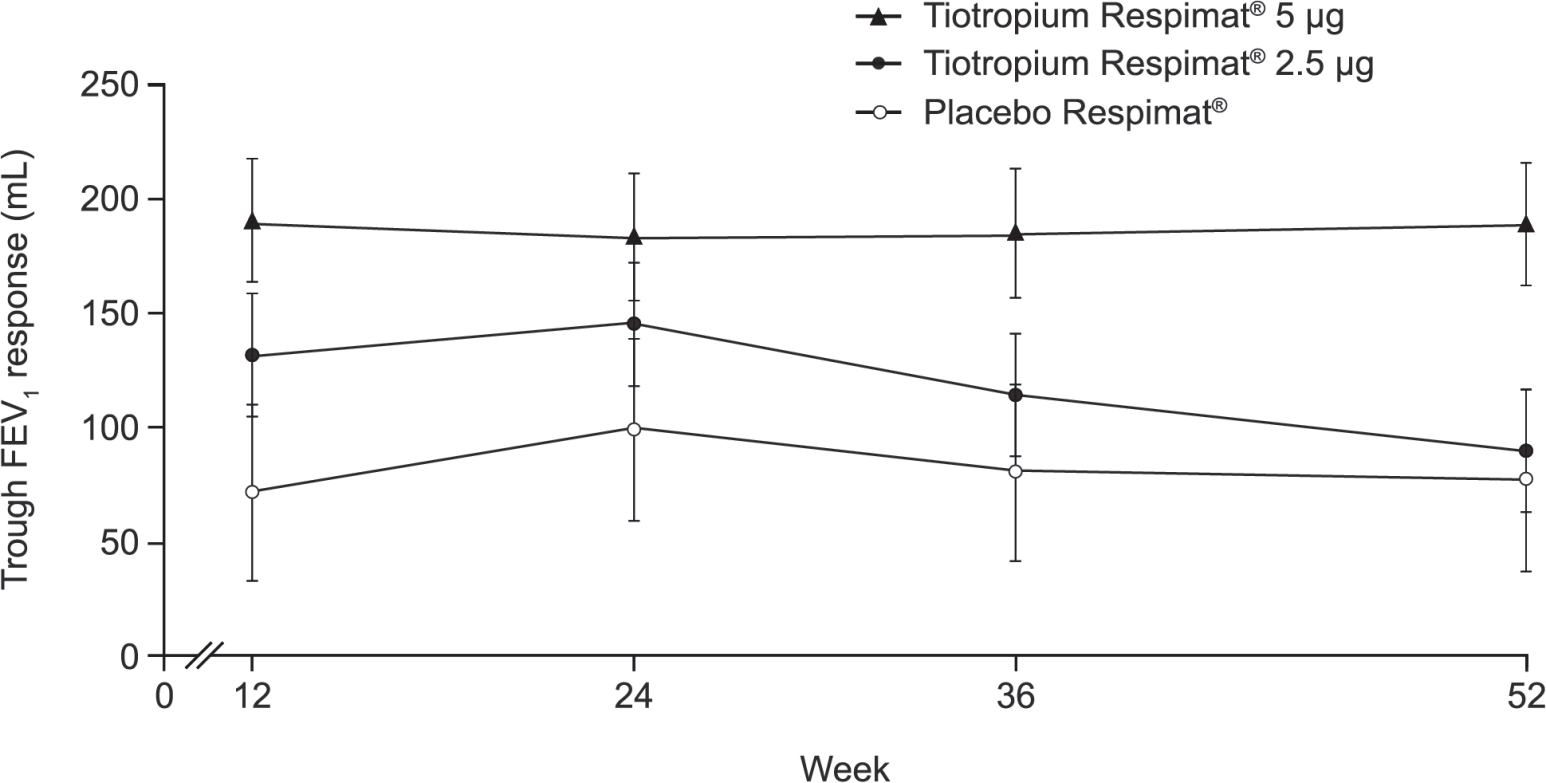
Trough FEV_1_ response for patients receiving tiotropium Respimat add-on or placebo Respimat add-on. Trough FEV_**1**_ response for patients receiving tiotropium Respimat or placebo Respimat as add-on to maintenance therapy of inhaled corticosteroids, with or without a long-acting β_**2**_-agonist, over the 52-week study period (full analysis set). Tiotropium Respimat and placebo Respimat dosed once-daily in the evening. Common baseline mean (standard deviation) at visit 2 (randomisation), mL: 2285 (645). FEV_**1**_, forced expiratory volume in 1 second.

Adjusted mean trough FEV_1_ response was significantly higher with tiotropium Respimat 5 μg than with placebo Respimat at Weeks 12, 36 and 52 (p = 0.0119 to 0.0295), with a treatment difference versus placebo Respimat at Week 52 of 112 mL (95% CI: 18, 207; p = 0.0203) (Fig 2). No statistically significant difference in adjusted mean trough FEV_1_ response was observed for tiotropium Respimat 2.5 μg versus placebo Respimat, with a treatment difference at Week 52 of 12 mL (95% CI: −82, 106; p = 0.7971). Trough forced vital capacity responses with tiotropium Respimat were not significantly different from those seen with placebo Respimat (Supporting Information).

**Fig 3:**
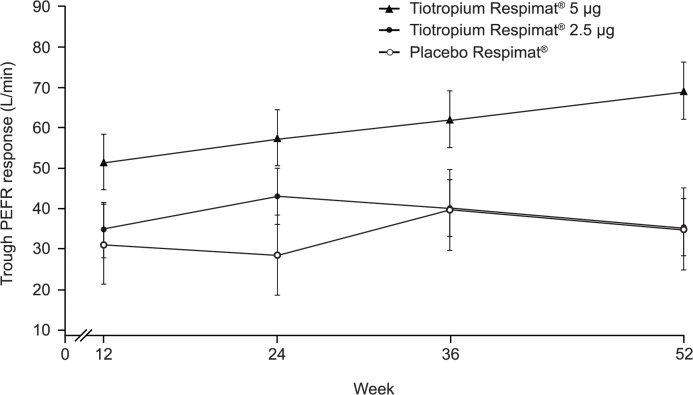
In-clinic trough PEFR response for patients receiving tiotropium Respimat add-on or placebo Respimat add-on. In-clinic trough PEFR response for patients receiving tiotropium Respimat or placebo Respimat as add-on to maintenance therapy of inhaled corticosteroids, with or without a long-acting β_**2**_-agonist, over the 52-week study period (full analysis set). Tiotropium Respimat and placebo Respimat dosed once-daily in the evening. Baseline mean (standard deviation) at visit 2 (randomisation), L/min: 371.0 (120.2). PEFR, peak expiratory flow rate.

At Weeks 24 and 52, adjusted mean in-clinic trough PEFR response was significantly higher with tiotropium Respimat 5 μg than with placebo Respimat (treatment difference: 28.9 L/min [95% CI: 5.047, 52.916; p = 0.0177] and 34.2 L/min [95% CI: 9.919, 58.432; p = 0.0058], respectively) (Fig 3). No significant difference in adjusted mean in-clinic trough PEFR response was observed for tiotropium Respimat 2.5 μg versus placebo Respimat; at Week 52, the treatment difference was 0.498 L/min (95% CI: −23.634, 24.630; p = 0.9677).

At Week 24, ACQ-7 responder rates improved, with numerically higher response rates observed in the tiotropium Respimat 5 μg (67.5%) and 2.5 μg (69.3%) groups compared with the placebo Respimat group (58.9%). However, by Week 52, ACQ-7 responder rates were similar across treatment groups: tiotropium Respimat 5 μg, 76.3%; tiotropium Respimat 2.5 μg, 71.1%; placebo Respimat, 73.2%.

## Discussion

Data presented here show that once-daily tiotropium Respimat add-on to current ICS maintenance therapy, with or without a LABA, had a safety and tolerability profile that was balanced compared with placebo Respimat in patients from Japan whose asthma was not being adequately controlled at baseline. Rates of SAEs and AEs leading to discontinuation were low. None of the SAEs occurring in patients receiving either dose of tiotropium Respimat was considered to be drug-related. The majority of AEs were mild to moderate in intensity.

The study sample size was based on local regulatory requirements, and the study was therefore not formally powered for safety or efficacy. Although this may be a limitation of the study, a placebo Respimat group was included that allows comparison and gives scope to compare the results described here with those obtained in other studies. In addition, the placebo Respimat arm may reduce possible reporting bias for drug-related AEs.

Overall, it appeared that rates of AEs were similar between treatment groups, and rates of SAEs were numerically lower in patients receiving tiotropium Respimat compared with those receiving placebo Respimat. Drug-related AEs were slightly more frequent with tiotropium Respimat 5 μg than with tiotropium Respimat 2.5 μg or placebo Respimat; most drug-related AEs were single occurrences and none was considered to be serious. None of the SAEs was considered by the treating physician to be drug-related.

The frequency of AEs (>86%) reported in all groups, including the placebo Respimat group, was higher than observed in other Phase II and III trials with tiotropium in populations with moderate to severe asthma [7–9]. However, in this study with a small sample size, the overall AE trends for tiotropium Respimat versus placebo Respimat were comparable with other trials, and no new safety signals were observed. The predominant AE in each group was nasopharyngitis, suggesting that the elevated AE rates may have been due to seasonal symptoms such as the common cold [11], or that air pollution may have contributed to respiratory illnesses that were reported as AEs [12]. It should also be noted that studies of long-term asthma treatments in Japanese patients tend to report relatively high AE rates. For example, AE rates were 81% and ≥90%, respectively, in a long-term safety study of fluticasone alone versus fluticasone combined with vilanterol (ClinicalTrials.gov identifier: NCT01244984), whereas in a study of fluticasone versus fluticasone/vilanterol combination that was conducted in 11 countries outside of Japan, the overall AE rates were 65% and 63% [13].

Previous research has prompted discussions surrounding the cardiac safety of inhaled anticholinergics in patients with respiratory diseases. For example, a meta-analysis of the cardiovascular risks associated with inhaled anticholinergics in patients with COPD identified an increased risk of cardiovascular death, myocardial infarction or stroke [14]. However, a randomised, placebo-controlled, 4-year trial that evaluated the long-term efficacy and safety of tiotropium given by HandiHaler in nearly 6000 patients with COPD [15] demonstrated reduced cardiac morbidity with tiotropium versus placebo, including a lower risk of serious cardiac events (relative risk: 0.84; p<0.05) such as myocardial infarction (relative risk: 0.71; p<0.05) and congestive heart failure (relative risk: 0.59; p<0.05). More recently, a study in 17,000 patients with COPD has shown that the safety profile of tiotropium Respimat 5 μg is comparable with that of tiotropium HandiHaler 18 μg, with an incidence of serious cardiac disorder AEs of 4.8% versus 4.7%, respectively [16]. In the current study, five patients (4.4%) receiving tiotropium Respimat 5 μg reported nine cardiac events, and one patient receiving tiotropium Respimat 2.5 μg reported a cardiac event. Five cardiac events in the tiotropium Respimat group were considered to be drug-related; these all occurred in the same patient. Although the investigator considered the cardiac events to be related to tiotropium therapy, the patient had co-morbid diagnoses of lumbar pain, hepatitis C virus and anaemia at baseline, and was continuously treated with intravenous peginterferon alfa-2 and ribavirin for hepatitis, both of which have been associated with cardiovascular events [17]. Furthermore, each cardiac event was mild in intensity, and it is worth considering whether the events may have caused morbidity or been detected outside of a clinical trial setting.

With regards to efficacy, this trial confirmed the long-term lung function benefits of tiotropium Respimat add-on to ICS with or without a LABA in patients from Japan. At Week 52, statistically greater responses that were in line with results from previous international Phase II and III trials [7–9] were observed in trough FEV_1_ response and in-clinic trough PEFR response for tiotropium Respimat 5 μg versus placebo Respimat. A previous subgroup analysis of an international tiotropium trial in patients with COPD demonstrated that the pattern of treatment benefit from tiotropium was similar in patients from Asia versus the global population [18]. Therefore, tiotropium Respimat 5 μg may be considered appropriate for long-term therapy.

The magnitude of improvement in lung function seen in the study presented here with the addition of tiotropium Respimat appears to be of a similar degree to the addition of a LABA, based on a Cochrane review of inhaled LABAs versus placebo on a background of ICS, which reported improvements in FEV_1_ and evening PEFR of 110 mL and 17.89 L/min, respectively, with LABAs [19]. This finding is in line with previous tiotropium Respimat trials that included an add-on salmeterol arm, and concluded that tiotropium as add-on to ICS was non-inferior to salmeterol as add-on to ICS [9,10].

ACQ-7 responder rates are reported as an indicator of asthma control (eg minimisation of symptoms, activity limitation, bronchoconstriction and rescue β_2_-agonist use); however, the ACQ-7 results presented here should also be interpreted with caution as the study was not designed to show statistical superiority in ACQ-7. Lung function improvements appeared to translate into improved asthma control in favour of tiotropium Respimat 5 μg over placebo Respimat at Week 24, but by Week 52 ACQ-7 responder rates were similar across all treatment groups. The improvements in ACQ-7 with the addition of tiotropium Respimat are even more striking considering that the majority of patients (almost 60%), including those in the placebo Respimat group, were receiving background ICS and LABA and the remainder at least ICS. Furthermore, placebo responses are not uncommon in asthma trials using ICS as background, likely attributable to improved adherence to ICS in clinical trial settings [20]. An earlier pooled analysis from two Phase III studies (NCT01172808 and NCT01172821) comparing tiotropium Respimat 5 μg or 2.5 μg or placebo Respimat also showed that, at 24 weeks, tiotropium Respimat 5 μg and 2.5 μg significantly improved the ACQ-7 responder rate compared with placebo [21]. Therefore, the totality of evidence suggests that tiotropium Respimat 5 μg use may have beneficial consequences for overall asthma control.

The efficacy of tiotropium Respimat 5 μg was greater than that of tiotropium Respimat 2.5 μg; the treatment difference for the 2.5 μg dose versus placebo Respimat peaked at Week 12 for trough FEV_1_ response and at Week 24 for in-clinic trough PEFR response, but did not clearly separate from placebo for either end point. The efficacy of the 5 μg dose has been demonstrated by previous studies [7–9], two of which enrolled Japanese patients [8], and the data presented here provide further support for the benefits of this dose. Compliance with the treatment regimen was high (>90%), indicating that the trial results are a reliable portrayal of the efficacy of tiotropium Respimat.

## Conclusions

In this trial, the tolerability profile of once-daily tiotropium Respimat was confirmed in patients from Japan who had moderate to severe symptomatic asthma while receiving medium-dose ICS, with or without a LABA, as maintenance therapy. The safety and efficacy profile of tiotropium Respimat reported in this trial was in line with previous studies and should be broadly applicable to other patient populations with symptomatic asthma. Over 52 weeks, AEs were generally mild and non-serious, and the overall tolerability profile of tiotropium Respimat was comparable with that of placebo Respimat. Lung function and symptom control data indicate that tiotropium Respimat shows sustained efficacy, with significant improvements over placebo Respimat in trough FEV_1_ and trough PEFR parameters over 1 year.

## Supporting Information

S1 CONSORT Checklist (DOCX)

S1 Fig Trough FVC response for patients receiving tiotropium Respimat add-on or placebo Respimat add-on. Trough FVC response for patients receiving tiotropium Respimat or placebo Respimat as add-on to maintenance therapy of inhaled corticosteroids, with or without long-acting β_2_-agonist, over the 52-week study period (full analysis set). Tiotropium Respimat and placebo Respimat dosed once-daily in the evening. FVC, forced vital capacity.
(TIF)

S1 File Trough forced vital capacity response. (DOCX)

S1 Protocol (PDF)

S1 Table Serious adverse events in each treatment group. (DOCX)
