# Supplementary figures and images for: Long-Term Once-Daily Tiotropium Respimat® Is Well Tolerated and Maintains Efficacy over 52 Weeks in Patients with Symptomatic Asthma in Japan: A Randomised, Placebo-Controlled Study
Source: PLoS One. 2015 Apr 20;10(4):e0124109. doi: 10.1371/journal.pone.0124109 (PMC4404354; doi:10.1371/journal.pone.0124109)

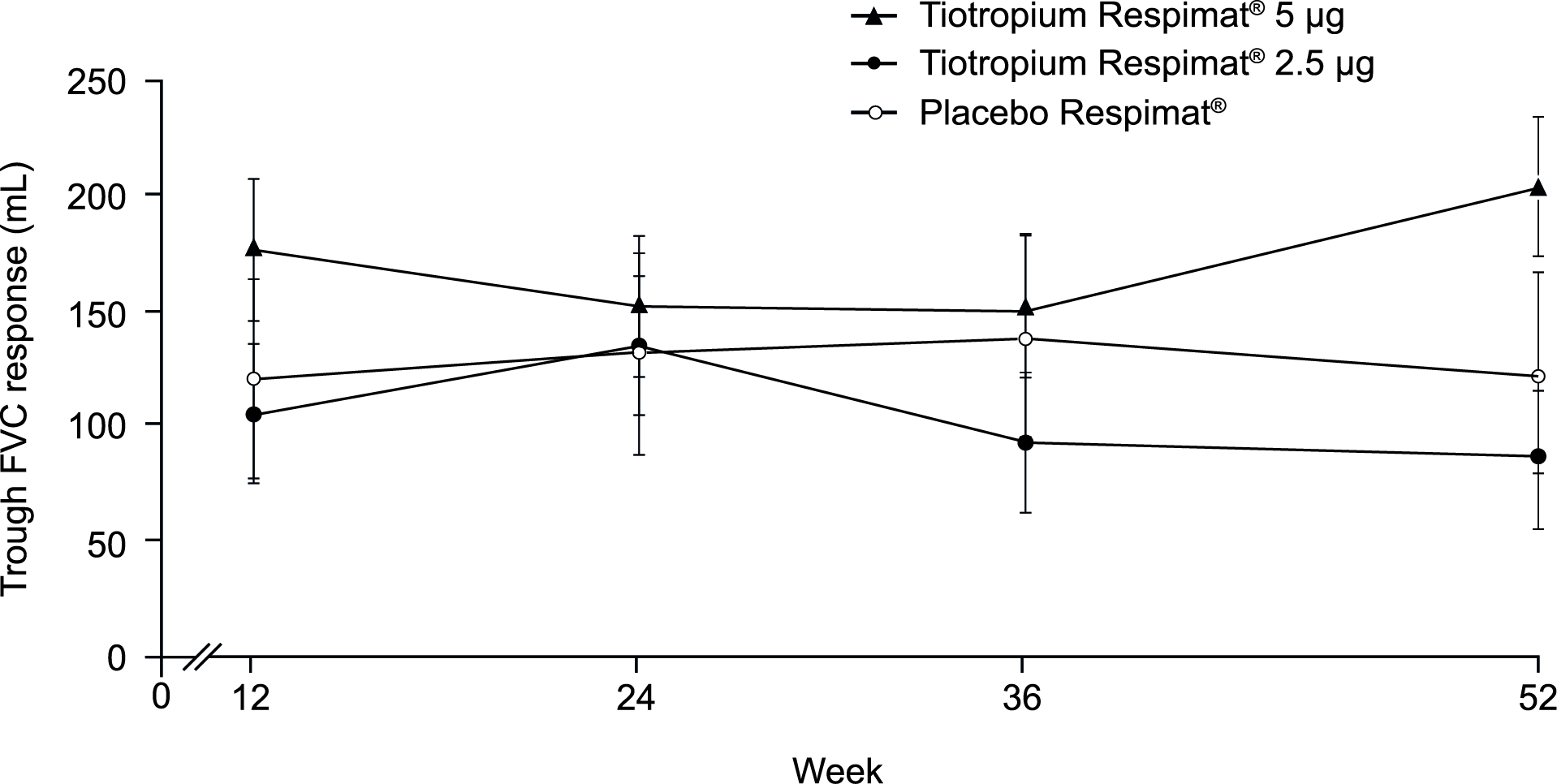

Supplement: S1 Fig — Trough FVC response for patients receiving tiotropium Respimat or placebo Respimat as add-on to maintenance therapy of inhaled corticosteroids, with or without long-acting β2-agonist, over the 52-week study period (full analysis set). Tiotropium Respimat and placebo Respimat dosed once-daily in the evening. FVC, forced vital capacity. (TIF) [file pone.0124109.s002.tif]
