# Supplementary material for: Long-Term Once-Daily Tiotropium Respimat® Is Well Tolerated and Maintains Efficacy over 52 Weeks in Patients with Symptomatic Asthma in Japan: A Randomised, Placebo-Controlled Study
Source: PLoS One. 2015 Apr 20;10(4):e0124109. doi: 10.1371/journal.pone.0124109 (PMC4404354; doi:10.1371/journal.pone.0124109)
Supplement: S1 File — (DOCX) [file pone.0124109.s003.docx]

**Trough forced vital capacity response**

Adjusted mean trough forced vital capacity responses were not significantly different from placebo Respimat^®^ for either dose of tiotropium Respimat^®^, although adjusted mean trough forced vital capacity response was numerically higher with tiotropium Respimat^®^ 5 µg than with placebo Respimat^®^ throughout the treatment period (Fig. S1). The treatment difference versus placebo Respimat^®^ at Week 52 was 82 mL (95% confidence interval: −23, 188; p=0.1270) in the tiotropium Respimat^®^ 5 µg group and −37 mL (95% confidence interval: −141, 68; p=0.4944) in the tiotropium Respimat^®^ 2.5 µg group.
