# Supplementary material for: Long-Term Once-Daily Tiotropium Respimat® Is Well Tolerated and Maintains Efficacy over 52 Weeks in Patients with Symptomatic Asthma in Japan: A Randomised, Placebo-Controlled Study
Source: PLoS One. 2015 Apr 20;10(4):e0124109. doi: 10.1371/journal.pone.0124109 (PMC4404354; doi:10.1371/journal.pone.0124109)
Supplement: S1 Protocol — (PDF) [file pone.0124109.s004.pdf]

Clinical Trial Protocol

Doc. No.: U10-4021-04

**BI Trial No.:**	205.464
**BI Investigational Product:**	Tiotropium inhalation solution - Respimat Inhaler (Ba 679 BR Respimat)
**Title:**	A Phase III randomised, double-blind, placebo-controlled, parallel-group trial to evaluate safety and efficacy of tiotropium inhalation solution delivered via Respimat inhaler (2.5 and 5 µg once daily) compared with placebo over 52 weeks in patients with moderate to severe persistent asthma
**Clinical Phase:**	III
**Trial Clinical Monitor:**	Satoko Kunimitsu<br>Clinical Research Department, Nippon Boehringer Ingelheim Co., Ltd.<br>2-1-1 ThinkPark Tower, Osaki, Shinagawa-ku, Tokyo 141-6017, Japan<br>TEL: +81 (0) 3 6417 2770<br>FAX: +81 (0) 3 5435 2932
**Coordinating investigator:**	Professor, Ken Ohta MD<br>**President**<br>**National Hospital Organization Tokyo National Hospital**<br>**3-1-1 Takeoka, Kiyose, Tokyo 204-8585, Japan**<br>**TEL: +81 (0) 42 491 2111**<br>**FAX: +81 (0) 42 494 2168**
**Status:**	Final Protocol (Revised Protocol (based on global amendment 3)
**Version and Date:**	**Version:4.0** **Date: 01 Jun 2012**

Proprietary confidential information.
© 2012 Boehringer Ingelheim International GmbH or one or more of its affiliated companies. All rights reserved.
This document may not - in full or in part - be passed on, reproduced, published or otherwise used without prior written permission.

CLINICAL TRIAL PROTOCOL SYNOPSIS

**Name of company:**<br><br>Boehringer Ingelheim	**Tabulated**<br>**Trial Protocol**	
**Name of finished product:**<br><br>Not applicable		
**Name of active ingredient:**<br><br>Tiotropium bromide - Respimat<br>(Ba 679 BR Respimat)		

**Protocol date:**<br>20 December 2010	**Trial number:**<br>205.464		**Revision date:**<br>**01 Jun 2012**

**Title of trial:**	A Phase III randomised, double-blind, placebo-controlled, parallel-group trial to evaluate safety and efficacy of tiotropium inhalation solution delivered via Respimat inhaler (2.5 and 5 µg once daily) compared with placebo over 52 weeks in patients with moderate to severe persistent asthma
**Coordinating investigator:**	Professor Ken Ohta, MD<br>**President,**<br>**National Hospital Organization Tokyo National Hospital**<br>**3-1-1 Takeoka, Kiyose, Tokyo 204-8585, Japan**
**Trial sites:**	Multi-centre trial
**Clinical phase:**	III
**Objectives:**	The primary objective is to evaluate the long term (52 weeks) safety of two doses (2.5 µg and 5 µg) of tiotropium inhalation solution (administered once daily) compared with placebo on top of maintenance therapy with inhaled corticosteroid (ICS) controller medication in patients with moderate to severe persistent asthma. Long term efficacy will also be evaluated under the same design as a secondary objective.
**Methodology:**	Randomised, double-blind, placebo-controlled, parallel-group
**No. of patients:**	
**total entered:**	280
**each treatment:**	Tiotropium 2.5 µg: 112<br>Tiotropium 5 µg: 112<br>Placebo: 56
**Diagnosis :**	Asthma
**Main criteria for inclusion:**	Outpatients of either sex, age 18-75 years, never-smokers or ex-smokers with<10 pack years and smoking cessation at least one year prior to enrolment. Patients must have at least a 12-week history of asthma that was diagnosed before the age of 40, and a current diagnosis of moderate to severe persistent asthma according to Global Initiative for Asthma (GINA) guideline. Patients need to be still symptomatic, i.e. not fully controlled with their current maintenance treatment [assessed by Asthma Control Questionnaire (ACQ) mean score and pulmonary function tests (PFTs)]. Maintenance treatment with a medium dose of ICS is required.
**Test products:**	Tiotropium inhalation solution-Respimat

Proprietary confidential information.
© 2012 Boehringer Ingelheim International GmbH or one or more of its affiliated companies. All rights reserved.
This document may not - in full or in part - be passed on, reproduced, published or otherwise used without prior written permission.

**Name of company:**<br><br>Boehringer Ingelheim		**Tabulated Trial Protocol**	
**Name of finished product:**<br><br>Not applicable			
**Name of active ingredient:**<br><br>Tiotropium bromide - Respimat<br>(Ba 679 BR Respimat)			
**Protocol date:**<br>20 December 2010	**Trial number:**<br>205.464		**Revision date:**<br>**01 Jun 2012**

**dose:**	2.5 µg (2 actuations of 1.25 µg) and 5 µg (2 actuations of 2.5 µg) once daily in the evening (17:00 - 20:00)
**mode of admin.:**	Oral inhalation via Respimat inhaler
**Comparator products:**	Placebo inhalation solution-Respimat
**dose:**	Not applicable
**mode of admin.:**	Oral inhalation via Respimat inhaler
**Duration of treatment:**	52 weeks
**Criteria for efficacy:**	Primary endpoints: No primary efficacy endpoint is defined in this study because the primary objective is to assess the long term safety in the patients with asthma who are treated with tiotropium delivered via the Respimat inhaler.<br><br>Secondary endpoints: Trough forced expiratory volume in one second (FEV_1) response; Trough forced vital capacity (FVC) response; individual in-clinic peak expiratory flow (PEF) measurements; PEF variability; Home assessment: PEF AM/PM, use of as occasion requires (PRN) rescue medication; daytime and nocturnal symptoms<br><br>Other endpoints: Home assessments during treatment period: FEV_1 AM/PM; Control of asthma (assessed by ACQ) at Visits 5 and 7
**Criteria for safety:**	Adverse events (AEs), vital signs, laboratory data, 12-lead electrocardiogram (ECG), vital status information.
**Statistical methods:**	Safety analysis:<br>Safety endpoints will be summarised in descriptive statistics.<br>Efficacy analysis:<br>・PFTs and ACQ:<br>Restricted maximum likelihood (REML)-based mixed effect model with repeated measures (MMRM) will be used to evaluate PFTs.<br>The model will include terms for treatment, test day, baseline and some of those terms interaction.<br>・Use of PRN rescue medication, Asthma symptoms, ACQ responder:<br>These endpoints will be summarised in descriptive statistics.

Proprietary confidential information.
© 2012 Boehringer Ingelheim International GmbH or one or more of its affiliated companies. All rights reserved.
This document may not - in full or in part - be passed on, reproduced, published or otherwise used without prior written permission.

FLOW CHART

Trial periods	Screening		Treatment*										Follow up
**Visit**	**0**	**1**	**2**	**3**	**3A**	**4**	**4A/ 4B**	**5**	**5A/ 5B**	**6**	**6A/ 6B/ 6C**	**7 (EoT***)**	**8**
**Week**	**-**	**-4**	**0**	**4**	**8**	**12**	**16/ 20**	**24**	**28/ 32**	**36**	**40/ 44/ 48**	**52**	**55**
**Day Time window****	**-**	**-28 ± 4**	**1**	**29 ± 7**	**57 ± 7**	**85 ± 7**	**113/ 141 ± 7**	**169 ± 7**	**197/ 225 ± 7**	**253 ± 7**	**281/ 309/ 337 ± 7**	**365 + 7**	**V7 + 21 + 7**
Informed consent[a)]	X												
Instruct patient on washout/restrictions[a)]	X	X	X	X		X		X		X		X	
Demographics		X											
Medical History/Baseline conditions[b)]		X											
Physical examination (incl. vital signs)		X	X	X		X		X		X		X[c)]	X[o)]
Review smoking status		X										X[c)]	
Laboratory test[d)]		X		X		X		X		X		X[c)]	X[o)]
ECG		X										X[c)]	
Pregnancy test[e)]		X										X[c)]	
Inclusion/exclusion criteria	X[a)]	X	X										
Dispense rescue medication	X	X	X	X	X	X	X	X	X	X	X	X	
Respimat training		X	X										
Randomisation			X										
Dispense study medication			X	X		X		X		X			
Administration of study medication in clinic[f)]			X			X		X		X		X	
Collect study medication				X		X		X		X		X[c)]	
Drug accountability		X[n)]	X[n)]	X	X	X	X	X	X	X	X	X[c)]	X[n)]
Training electronic diary (eDiary) with PEF-meter		X	X										
Issue eDiary with PEF-meter		X											
Download eDiary with PEF-meter			X[g)]	X[g)]	X[g)]	X[g)]	X[g)]	X[g)]	X[g)]	X[g)]	X[g)]	X[c), g)]	
Collect eDiary with PEF-meter												X[c)]	
Issue paper diary card		X	X	X		X		X		X			
Review/collect paper diary card			X	X	X	X	X	X	X	X	X	X[c)]	
ACQ[h)]		Xi)	Xi)					X				X	
Review exacerbation			X	X	X	X	X	X	X	X	X	X	
Medication washout check[j)]		X	X			X		X		X		X	
PFT[k)]		X[k)]	X[l)]			X[l)]		X[l)]		X[l)]		X[l)]	
Vital signs (seated) (during PFTs)			X[m)]			X[m)]		X[m)]		X[m)]		X[m)]	
AEs		X	X	X	X	X	X	X	X	X	X	X[c)]	X
Concomitant therapy		X	X	X	X	X	X	X	X	X	X	X[c)]	X
Vital status		X	X	X	X	X	X	X	X	X	X	X[c)]	X[c)]
Termination of study medication												X[c)]	
Completion of trial													X

Proprietary confidential information.
© 2012 Boehringer Ingelheim International GmbH or one or more of its affiliated companies. All rights reserved.
This document may not - in full or in part - be passed on, reproduced, published or otherwise used without prior written permission.

Note: Patient should visit site every four weeks in between visits (Visits 3 to 7) (i.e. once between Visits 3 and 4 [Week 8], twice between Visits 4 and 5 [Weeks 16 and 20] and Visits 5 and 6 [Weeks 28 and 32], three times between Visits 6 and 7 [Weeks 40, 44 and 48]) in order to collect safety information and ensure correct drug accountability (study medication and use of PRN rescue medication) and concomitant medication.

* Visits 0, 3, 3A, 4A/B, 5A/B, 6A/B/C, and 8 may be conducted during business hours. Visits 1 to 7 except Visit 3, 3A, 4A/B, 5A/B, and 6A/B/C will always start in the evening in order to perform PFTs and study medication administration between 17:00 and 20:00.

** Each Respimat inhaler contains drug supply for 30 days.

*** End of trial (EoT)

a) All patients must sign an informed consent consistent with International Conference on Harmonisation-Good Clinical Practice (ICH-GCP) guidelines and Good Clinical Practice (GCP) prior to participation in the trial, which includes medication washout and restrictions (see Section 4.2.2). The interval between Visit 0 and Visit 1 may be between 1 and 28 days depending on medication washout requirements and restrictions. A preliminary check of in-/exclusion criteria is recommended at Visit 0 to avoid unnecessary washout procedures in non-eligible patients.

b) Including asthma background characteristics.

c) To be completed by all patients who take at least one dose of study medication including those who discontinue early. If they discontinue prior to completion of treatment, it is recommended to be performed of X[c)] at Visit 7 and the data will be recorded on electronic case report form (eCRF) at Visit 7 as EoT and vital status information has to be collected on the originally planned follow up visit date (Visit 8).

d) Haematology and blood chemistry (central laboratory). White blood cell count, eosinophil count (absolute and relative), total serum immunoglobulin E (IgE) and creatinine levels will be documented in eCRF only at Visit 1.

e) Urine pregnancy test required for all women of child-bearing potential.

f) Medications are administered in the following fixed sequence: 1. ICS (if regular medication/schedule for the patient) and leukotriene modifier (LTRA) (if applicable), 2. study medication from assigned Respimat because time point zero for PFTs is defined as the complete time of inhalation (i.e. end time of the second inhalation from Respimat).
Patient's ICS should be administered without change in posology (b.i.d., q.d., AM, PM), i.e. as prescribed by patient's treating physician prior to this trial entry.

g) e-Diary compliance check (see Sections 4.3 and 6.1). At between visit (Visit 3A, 4A/B, 5A/B, 6A/B/C), download eDiary with PEF-meter can be conducted.

h) ACQ will be patient self-administered at the beginning of the visit and should not precede any discussion with a health professional prior to completion of the questionnaire.

i) ACQ at screening will be used for assessment of degree of asthma control. If the patient is not eligible due to the predefined score at Visit 1, the patient should not be further evaluated. If the patient is not eligible due to the predefined score at Visit 2, the patient's Visit 2 can be repeated only once for further assessment (see Section 6.1).

j) Refer to Section 4.2.2.1.

k) 10 minutes pre- and 15-30 minutes post-bronchodilator PFT after inhalation of 4 actuations (100 μg/actuation) salbutamol.

l) 10 minutes prior to trial drug administration (pre-dose).

m) In conjunction with pulmonary function testing at pre-dose (measured immediately before PFT).

n) Rescue medication only.

o) To be performed only if relevant findings at Visit 7, which is judged at the discretion of the investigator.

Proprietary confidential information.
© 2012 Boehringer Ingelheim International GmbH or one or more of its affiliated companies. All rights reserved.
This document may not - in full or in part - be passed on, reproduced, published or otherwise used without prior written permission.

TIMING OF TRIAL PROCEDURES DURING THE TREATMENT PERIOD

PFT at Visits 2, 4, 5, 6 and 7[a)]

<table>
<tr><th rowspan="2"></th><th colspan="5">Timing related to evening inhalation of study drug</th></tr>
<tr><th>-1h</th><th>-30 min</th><th>-15 min</th><th>-10 min</th><th>0</th></tr>
<tr><td>Administer patient's usual ICS medication followed by study medication[b)]</td><td></td><td></td><td></td><td></td><td>X</td></tr>
<tr><td>Asthma Monitor® 3 (AM3)</td><td colspan="2">←→</td><td></td><td></td><td></td></tr>
<tr><td>Patient self-administration of questionnaire[c)]</td><td colspan="3">←→</td><td></td><td></td></tr>
<tr><td>Vital signs (seated)</td><td></td><td></td><td></td><td>X</td><td></td></tr>
<tr><td>PFT</td><td></td><td></td><td></td><td>X</td><td></td></tr>
</table>

a) Order of procedures if performed at the same time point:
- Vital signs followed by pulmonary function testing
- Use of AM3 device followed by filling out questionnaire

b) Evening trial drug administration will occur between 17:00 and 20:00 and within ± 30 minutes of time of administration at Visit 2. Medications are administered in a fixed sequence (as described in footnote f) of the main flowchart). Time point zero for PFTs is defined as the complete time of inhalation (i.e. end time of the second inhalation from Respimat).

c) ACQ will be patient self-administered prior to any discussion with a health professional only at Visits 2, 5 and 7.

Proprietary confidential information.
© 2012 Boehringer Ingelheim International GmbH or one or more of its affiliated companies. All rights reserved.
This document may not - in full or in part - be passed on, reproduced, published or otherwise used without prior written permission.

TABLE OF CONTENTS

TITLE PAGE....1
CLINICAL TRIAL PROTOCOL SYNOPSIS 2
FLOW CHART 4
TABLE OF CONTENTS....7
ABBREVIATIONS 10
1. INTRODUCTION.... 12
1.1 MEDICAL BACKGROUND....12
1.2 DRUG PROFILE 12
1.2.1 Inhalation solution and Respimat Inhaler....14
2. RATIONALE, OBJECTIVES, AND BENEFIT - RISK ASSESSMENT.... 15
2.1 RATIONALE FOR PERFORMING THE TRIAL 15
2.2 TRIAL OBJECTIVES....15
2.3 BENEFIT - RISK ASSESSMENT 15
3. DESCRIPTION OF DESIGN AND TRIAL POPULATION.... 17
3.1 OVERALL TRIAL DESIGN AND PLAN 17
3.1.1 Administrative structure of the trial....18
3.2 DISCUSSION OF TRIAL DESIGN, INCLUDING THE CHOICE OF CONTROL GROUP....19
3.3 SELECTION OF TRIAL POPULATION 20
3.3.1 Main diagnosis for study entry....20
3.3.2 Inclusion criteria 20
3.3.3 Exclusion criteria 21
3.3.4 Removal of patients from therapy or assessments....23
3.3.4.1 Removal of individual patients....23
3.3.4.2 Discontinuation of the trial by the sponsor....24
4. TREATMENTS.... 25
4.1 TREATMENTS TO BE ADMINISTERED....25
4.1.1 Identity of BI investigational product and comparator product....25
4.1.2 Method of assigning patients to treatment groups 26
4.1.3 Selection of doses in the trial....26
4.1.4 Drug assignment and administration of doses for each patient 26
4.1.5 Blinding and procedures for unblinding 29
4.1.5.1 Blinding....29
4.1.5.2 Procedures for emergency unblinding 29
4.1.6 Packaging, labelling, and re-supply 29
4.1.7 Storage conditions....31
4.1.8 Drug accountability 31

Proprietary confidential information.
© 2012 Boehringer Ingelheim International GmbH or one or more of its affiliated companies. All rights reserved.
This document may not - in full or in part - be passed on, reproduced, published or otherwise used without prior written permission.

4.2 CONCOMITANT THERAPY, RESTRICTIONS, AND RESCUE TREATMENT 32
4.2.1 Rescue medication, emergency procedures, and additional treatments 33
4.2.1.1 Rescue medication 33
4.2.1.2 Emergency procedures 33
4.2.1.3 Additional treatments 33
4.2.2 Restrictions 34
4.2.2.1 Restrictions regarding concomitant treatment 34
4.2.2.2 Restrictions on diet and life style 39
4.3 TREATMENT COMPLIANCE 39
5. VARIABLES AND THEIR ASSESSMENT 40
5.1 EFFICACY 40
5.1.1 Endpoints of efficacy 40
5.1.1.1 Primary endpoints 40
5.1.1.2 Secondary endpoints 40
5.1.1.3 Other endpoints 40
5.1.2 Assessment of efficacy 41
5.2 SAFETY 44
5.2.1 Endpoints of safety 44
5.2.2 Assessment of adverse events 44
5.2.2.1 Definitions of adverse events 44
5.2.2.2 Adverse event and serious adverse event reporting 46
5.2.3 Assessment of safety laboratory parameters 47
5.2.4 Electrocardiogram 48
5.2.5 Assessment of other safety parameters 49
5.3 APPROPRIATENESS OF MEASUREMENTS 50
6. INVESTIGATIONAL PLAN 51
6.1 VISIT SCHEDULE 51
6.2 DETAILS OF TRIAL PROCEDURES AT SELECTED VISITS 52
6.2.1 Screening and run-in period 52
6.2.2 Treatment period 54
6.2.3 End of trial and follow-up period 56
7. STATISTICAL METHODS AND DETERMINATION OF SAMPLE SIZE 59
7.1 STATISTICAL DESIGN - MODEL 59
7.2 NULL AND ALTERNATIVE HYPOTHESES 60
7.3 PLANNED ANALYSES 60
7.3.1 Primary analyses 60
7.3.2 Secondary analyses 60
7.3.3 Safety analyses 61
7.3.4 Interim analyses 61

Proprietary confidential information.
© 2012 Boehringer Ingelheim International GmbH or one or more of its affiliated companies. All rights reserved.
This document may not - in full or in part - be passed on, reproduced, published or otherwise used without prior written permission.

7.4 HANDLING OF MISSING DATA 61
7.5 RANDOMISATION 62
7.6 DETERMINATION OF SAMPLE SIZE 62
8. INFORMED CONSENT, DATA PROTECTION, TRIAL RECORDS 63
8.1 STUDY APPROVAL, PATIENT INFORMATION, AND INFORMED CONSENT 63
8.2 DATA QUALITY ASSURANCE 64
8.3 RECORDS 64
8.3.1 Source documents 64
8.3.2 Direct access to source data and documents 64
8.3.3 Storage of records 64
8.4 LISTEDNESS AND EXPEDITED REPORTING OF ADVERSE EVENTS 65
8.4.1 Listedness 65
8.4.2 Expedited reporting to health authorities and IECs/IRBs 65
8.5 STATEMENT OF CONFIDENTIALITY 65
8.6 COMPLETION OF TRIAL 65
8.7 PROTOCOL VIOLATIONS 65
8.8 COMPENSATION AVAILABLE TO THE PATIENT IN THE EVENT OF TRIAL RELATED INJURY 65
9. REFERENCES 66
9.1 PUBLISHED REFERENCES 66
9.2 UNPUBLISHED REFERENCES 66
10. APPENDICES 68
10.1 INSTRUCTIONS FOR THE USE OF THE RESPIMAT INHALER 68
10.2 INSTRUCTIONS FOR RETURN OF MALFUNCTIONING RESPIMAT INHALERS 74
10.3 ADDITIONAL INFORMATION REGARDING IN/EX CRITERIA 75
10.4 ASTHMA CONTROL QUESTIONNAIRE (ACQ) 77
10.5 PAPER PATIENT DIARY CARD 80
10.6 AM3 PATIENT INSTRUCTION CARD 81
10.7 DEFINITION ASTHMA EXACERBATION 82
10.8 CLINICAL LAB PARAMETERS 84
11. DESCRIPTION OF GLOBAL AMENDMENT 85

Proprietary confidential information.
© 2012 Boehringer Ingelheim International GmbH or one or more of its affiliated companies. All rights reserved.
This document may not - in full or in part - be passed on, reproduced, published or otherwise used without prior written permission.

ABBREVIATIONS

ACQ	Asthma Control Questionnaire
ADRB2	beta 2-adrenergic receptor
AE(s)	adverse event(s)
ALT	alanine aminotransferase
AM3	Asthma Monitor® 3
ATS	American Thoracic Society
AST	aspartate aminotransferase
BAC	benzalkonium chloride
BI	Boehringer Ingelheim
BPRM	blineded report planning meeting
CML(s)	clinical monitor local(s)
COPD	chronic obstructive pulmonary disease
CRA(s)	clinical research assistant(s)/associate(s)
CRF	case report form
CRO	contract research organisation
CTMF	clinical trial master file
CTP	clinical trial protocol
CTR	clinical trial report
CVA(s)	cerebrovascular accident(s)
DSMB	data safety monitoring board
ECG	electrocardiogram
eCRF	electronic case report form
ECSC	European Community for Steel and Coal
eDiary	electronic diary
EDTA	ethylenediaminetetraacetic acid
EoT	end of trial
ERS	European Respiratory Society
EU	European Union
FAS	full analysis set
FDA	Food and Drug Administration
FEV_1	forced expiratory volume in one second
FVC	forced vital capacity
γ-GT	gamma-glutamyltransferase
GCP	Good Clinical Practice
GINA	Global Initiative for Asthma
h(s)	hour(s)
HFA	hydrofluororalkane
IB	investigator's brochure
ICH	International Conference on Harmonisation
ICS	inhaled corticosteroids
IgE	immunoglobulin E
INN	international non-proprietary name
IRB	institutional review board
ISF	investigator site file
IVRS	interactive voice response system
IWRS	interactive web response system
kg	kilogram

Proprietary confidential information.
© 2012 Boehringer Ingelheim International GmbH or one or more of its affiliated companies. All rights reserved.
This document may not - in full or in part - be passed on, reproduced, published or otherwise used without prior written permission.

L	litre(s)
LABA	long-acting beta-adrenergic
LDH	lactate dehydrogenase
LOCF	last observation carried forward
LTRA	leukotriene receptor antagonist (leukotriene modifier)
µg	microgram
MCID	minimum clinically important difference
MDI	metered dose inhaler
MedDRA	Medical Dictionary for Regulatory Activities
mg	milligram
MHLW	Ministry of Health, Labour and Welfare
min	minimal; minute
mL	millilitre(s)
MMRM	mixed effect model with repeated measures
NBI	Nippon Boehringer Ingelheim
No.	number
QOL	quality of life
PEF	peak expiratory flow
PFT	pulmonary function test
PMDA	Pharmaceuticals and Medical Devices Agency
PRN	pro re nata (as occasion requires)
REML	restricted maximum likelihood
RDC	Remote Data Capture (eCRF)
SABA	short-acting beta-adrenergic
SAE(s)	serious adverse event(s)
SGOT	serum glutamic oxaloacetic transaminase
SGPT	serum glutamic pyruvic transaminase
SOP(s)	standard operating procedures
SPC	summary of product characteristics
SUSAR	suspected unexpected serious adverse reaction
TCM	trial clinical monitor
TLC	total lung capacity
TNF	tumor necrosis factor
TS	treated set
TSAP	trial statistical analysis plan
ULN	upper limit of normal

Proprietary confidential information.
© 2012 Boehringer Ingelheim International GmbH or one or more of its affiliated companies. All rights reserved.
This document may not - in full or in part - be passed on, reproduced, published or otherwise used without prior written permission.

1. INTRODUCTION

1.1 MEDICAL BACKGROUND

Asthma is a chronic inflammatory disorder of the airways in which many cells and cellular products play a role. The overall worldwide prevalence of asthma is about 5%, affecting 300 million people worldwide with over 60 million affected in the United States and Europe and approximately two million affected in Japan and high variability from country to country. In Japan, symptom prevalence of asthma has been increasing rapidly in recent years. It is estimated that the average prevalence in our country increased from about 1% in the 1960s to about 6% recently in children, and from a little less than 1% to about 3% in adults (P10-03196 and R10-4989).

Central to the various phenotypic patterns of asthma is the presence of chronic underlying airway inflammation. The inflammatory cell components involved are variable, but with overlapping patterns that reflect the different phenotypes of the disease, such as intermittent versus persistent or acute versus chronic manifestations.The inflammation causes airway hyperresponsiveness that leads to recurrent episodes of wheezing, breathlessness, chest tightness, and coughing, particularly at night or in the early morning. These episodes are usually associated with widespread but variable airflow obstruction that is often reversible either spontaneously or with treatment (P10-03196).

Tiotropium is a quaternary ammonium compound developed as a long-acting orally inhaled anticholinergic bronchodilator. Anticholinergics are considered as a first-line therapy in chronic obstructive pulmonary disease (COPD) and there is a large body of evidence demonstrating its efficacy and safety, whereas, the place of anticholinergics in the treatment of asthma is less well-defined, particularly in patients with not optimally controlled or uncontrolled asthma. Patients with severe persistent asthma who are inadequately controlled despite treatment with a combination of inhaled steroids (ICS)/long-acting beta-adrenergic (LABA) therapy are a therapeutic challenge with significant unmet medical need. An additional anticholinergic bronchodilator may provide added benefits for these patients. For some patients still symptomatic on maintenance therapy with an ICS alone, treatment with a long-acting anticholinergic could be an alternative bronchodilator controller medication instead of a LABA. It is anticipated helpful especially for a subpopulation of patients with asthma who has a risk for severe exacerbations or death with use of LABA.

1.2 DRUG PROFILE

The beneficial effect of tiotropium on bronchoconstriction is well established and clinically used for years in the treatment of COPD. It is three to eight-fold more potent than ipratropium bromide and has a slower onset action. The duration of action for tiotropium exceeds 24 hours compared to 6 hours for ipratropium bromide. As with other inhaled quaternary anticholinergic agents, tiotropium does not cross the blood-brain barrier to any relevant extent. It has a slow oral bioavailability. Clinical trials have established that the adverse event (AE) profile of tiotropium follows that of ipratropium bromide. In clinical trials with 2-8 times the recommended therapeutic dose of tiotropium 18 µg up to 144 µg in healthy volunteers, there were no clinically significant changes in vital signs or laboratory parameters.

Proprietary confidential information.
© 2012 Boehringer Ingelheim International GmbH or one or more of its affiliated companies. All rights reserved.
This document may not - in full or in part - be passed on, reproduced, published or otherwise used without prior written permission.

Two product formulations of tiotropium have been registered in Japan. The first formulation is a single-dose capsule containing 18 µg of tiotropium (equivalent to 22.5 µg of tiotropium bromide monohydrate) formulated in a powder blend with lactose monohydrate. The second product is an aqueous solution of tiotropium formulated with the excipients benzalkonium chloride (BAC) and ethylenediaminetetraacetic acid, disodium salt (EDTA) (2.5 µg tiotropium per actuation, 2 actuations per dose), which is intended for oral inhalation only via the Respimat inhaler. Direct comparability regarding efficacy and safety between the two products, tiotropium inhalation solution/Respimat and tiotropium inhalation powder/HandiHaler is provided from the three Phase III trials (BI Trial Nos. 205.249, 205.250, and 205.291) (U05-1949, U04-2041, and U07-3262) and non-inferiority was established for tiotropium inhalation solution 5µg /Respimat compared to tiotropium inhalation powder18 µg /HandiHaler.

Four randomised clinical trials have been conducted in patients with asthma using the inhalation powder capsule formulation of tiotropium (BI Trial Nos. 205.121, 205.201, 205.202, and 205.203) (U96-0240, U98-3174, U98-3274, U99-1019). These trials in the general (and exercise-induced) asthma population have demonstrated that tiotropium provides some degree of bronchodilation in asthmatic patients. The incidence of AEs was low in all four asthma trials using doses up to 36 µg inhalation powder/HandiHaler over 21 to 28 days of treatment.

Three trials have been conducted with Spiriva Respimat in patients with asthma (BI Trial Nos. 205.248, 205.341, and 205.342).

Boehringer Ingelheim (BI) Trial No. 205.248 was a Phase II, single-dose, randomised, double-blind (within-device), four way crossover trial conducted to evaluate the local tolerability of an acidic solution (pH=2.7) for inhalation with the Spiriva Respimat placebo solution. This trial was conducted in 34 hypersensitive asthmatic patients. No adverse effects were attributed to the acidic solution, which was well tolerated. Neither spirometric parameters nor vital signs were changed by study treatment (U02-1222).

BI Trial No. 205.341 was a Phase II proof-of-concept study and an 8-week randomised, placebo-controlled, double-blind, 3-way crossover comparison of 5 µg and 10 µg Spiriva Respimat and placebo Respimat administered once daily in the morning as add-on therapy in 100 adult asthmatics with maximised controller medication, who were still symptomatic (U08-2081).

BI Trial No. 205.342 was a Phase II proof-of-concept study and a 16-week randomised, placebo- and active-controlled, double-blind, double-dummy, parallel-group study comparing the efficacy and safety of Spiriva Respimat (5 µg once daily) in the evening with that of salmeterol hydrofluororalkane metered dose inhaler (HFA MDI) (2 actuations of 25 µg twice daily) both in addition to maintenance ICS in the patients with moderate persistent asthma and homozygote for arginine at beta 2-adrenergic receptor (ADRB2) (U09-1701).

Above two completed Phase II proof-of-concept trials (BI Trial Nos. 205.341 and 205.342) showed a significant efficacy signal and a favourable safety profile for tiotropium administered via the Respimat inhaler in the patients with moderate or severe persistent asthma, who were adequately treated with ICS according to current treatment guidelines. All trials for tiotropium Respimat in asthma were and will be conducted only with an appropriate maintenance treatment with an ICS.

Proprietary confidential information.
© 2012 Boehringer Ingelheim International GmbH or one or more of its affiliated companies. All rights reserved.
This document may not - in full or in part - be passed on, reproduced, published or otherwise used without prior written permission.

Two identical 1-year (48-week treatment period) phase III trials (BI Trial Nos. 205.416 and 205.417) are currently in conduct to confirm the safety and efficacy of 5 µg tiotropium inhalation solution (on top of at least ICS and LABA) in patients with severe persistent asthma. Another two identical 6-month (24-week treatment period) phase III trials (BI Trial Nos. 205.418 and 205.419) are also currently in conduct to evaluate the safety and efficacy of 2.5 and 5 µg tiotropium inhalation solution (on top of ICS) in patients with moderate persistent asthma. Japanese patients participate in both of the phase III studies.

Detailed outline of the existing quality, non-clinical and clinical data of tiotropium is described in the "Investigator's Brochure (IB)" (U92-0551).

1.2.1 Inhalation solution and Respimat Inhaler

Active ingredient solution

The tiotropium inhalation solution is aqueous based. The pH value is adjusted to pH 2.9 ± 0.2, near the stability optimum of the active substance.

Administration of tiotropium inhalation solution is achieved with the Respimat inhaler in combination with a drug reservoir/cartridge. The drug is delivered from the Respimat inhaler as two actuations per dose. As a multi-dose device and solution, the drug formulation contains EDTA and the bacteriostatic agent, benzalkonium chloride (BAC), which have been reported to induce bronchospasm in some patients inhaling such solutions from a nebuliser. However, the doses of EDTA and BAC administered with two actuations of the Respimat are well below the amounts for which bronchospasm has been reported with nebulised solutions. Additionally, clinical data for the Respimat inhaler with a variety of drug substances (including tiotropium) indicate that it is unlikely that patients using the Respimat inhaler will experience an EDTA or preservative-related bronchospasm (see Section 6.2.4.4.4 of the tiotropium IB for further information) (U92-0551).

Details of the Respimat device and the cartridge for active ingredient solution and the instructions for use are found in Appendix 8.2 of the tiotropium IB (U92-0551) and in this protocol (Appendix 10.1).

Proprietary confidential information.
© 2012 Boehringer Ingelheim International GmbH or one or more of its affiliated companies. All rights reserved.
This document may not - in full or in part - be passed on, reproduced, published or otherwise used without prior written permission.

2. RATIONALE, OBJECTIVES, AND BENEFIT - RISK ASSESSMENT

2.1 RATIONALE FOR PERFORMING THE TRIAL

Rationale for performing this trial is to evaluate long term safety of the 2.5 and 5 µg tiotropium inhalation solution (on top of ICS) in approximately 100 Japanese patients with asthma per each dose based on International Conference on Harmonisation- Good Clinical Practice (ICH-GCP) E1 Guideline and the data from this trial will be submitted to Japanese regulatory authority [Pharmaceuticals and Medical Devices Agency (PMDA)] for the registration of tiotropium in an indication for asthma.

The data collected in this trial will provide useful information to health care providers and patients regarding the safety and secondarily the efficacy of a once daily inhalation of two doses of tiotropium inhalation solution delivered by the Respimat inhaler in addition to ICS in the treatment of not fully controlled moderate to severe asthma in comparison to placebo.

2.2 TRIAL OBJECTIVES

The primary objective of this trial is to evaluate the long term (52 weeks) safety of two doses (2.5 µg and 5 µg) of tiotropium inhalation solution (administered once daily in the evening delivered by the Respimat inhaler) compared with placebo on top of maintenance therapy with ICS controller medication in patients with moderate to severe persistent asthma. The secondary objective of this trial is to evaluate the long term efficacy with the same dose regimen and population.

Refer to Section 5 for the endpoints.

2.3 BENEFIT - RISK ASSESSMENT

The favourable benefit-risk ratio based on the so far acquired knowledge about inhaled tiotropium is the rationale to conduct further studies with tiotropium in asthma.

The incidence of AEs was low in all four asthma trials using doses up to 36 µg inhalation powder/HandiHaler over 21 to 28 days of treatment (BI Trial Nos. 205.121, 205.201, 205.202, and 205.203). The type and rate of events were not different from those seen in the trials with COPD patients, aside from “asthma exacerbation”. The most common events were asthma exacerbation, upper respiratory infection, headache and dry mouth. Single doses of placebo inhalation solution/Respimat were well tolerated, as evaluated in 32 mild asthmatic patients.

In BI Trial No. 205.341 the differences in the primary endpoint which was change in peak forced expiratory volume in one second (FEV_1) after 8 weeks of treatment between the active dose groups and the placebo group were statistically significant ($p<0.0001$). In the subset group who underwent 24-hour lung function testing, the differences in FEV_1 response between the active treatment groups and the placebo group were statistically significant ($p<0.01$), thus demonstrating that the duration of effect with tiotropium was at least 24 hours. During this study, the overall occurrence of AEs was similar between the placebo and 5 µg tiotropium groups (39.8% and 42.3% of patients, respectively, reported at least one AE), but slightly higher in the 10 µg tiotropium group (49.5% of patients reported at least one AE). The most common treatment-emergent AEs were nasopharyngitis and asthma [Medical Dictionary for Regulatory Activities (MedDRA) preferred

Proprietary confidential information.
© 2012 Boehringer Ingelheim International GmbH or one or more of its affiliated companies. All rights reserved.
This document may not - in full or in part - be passed on, reproduced, published or otherwise used without prior written permission.

term classification including aggravated asthma and exacerbation of asthma], with both being reported overall by 28 patients (26.2%). The only treatment-emergent AE reported in more than one patient, which was considered drug-related and observed in 4 patients (3.9%) only in the 10 µg tiotropium group, was dry mouth. None of the serious adverse events (SAEs) were considered drug-related and none were fatal or life threatening (U08-2081).

During the double-blind treatment and follow-up period of trial (BI Trial No. 205.342), mean (±standard deviation) duration of double-blind exposure to study medication was 109.6 (± 21.3) days (placebo), 110.9 (± 16.2) days (tiotropium), and 111.8 (± 16.8) days (salmeterol). In this study, the primary endpoint, the change in mean weekly morning peak expiratory flow (PEF) from baseline to the last week of treatment, demonstrated the statistical non-inferiority of 5 µg tiotropium inhalation solution/Respimat versus salmeterol and its superiority versus placebo. Thus, 5 µg tiotropium inhalation solution/Respimat was as effective as salmeterol in the treatment of patients homozygous for arginine at the 16th amino acid position of the ADRB2 (B16-Arg/Arg) with moderate persistent asthma. During the double-blind treatment and follow-up periods, the overall incidence of AEs was similar in the active treatment and placebo groups: 52 (41.3%) placebo patients, 51 (39.8%) tiotropium patients, and 56 (41.8%) salmeterol patients. Few AEs were considered drug-related and the incidences of such AEs were also similar across groups: 4 (3.2%) placebo patients, 6 (4.7%) tiotropium patients, and 3 (2.2%) salmeterol patients. The most common AEs by preferred term were asthma exacerbation (including preferred term asthma) and nasopharyngitis. No SAEs were considered to be drug-related (U09-1701).

In conclusion, the administration of tiotropium can be considered as safe for patients with ashma.

In two completed Phase II proof-of-concept trials (BI Trial Nos. 205.341 and 205.342) no untoward events happened to patients treated with placebo and the overall incidence of AEs and the incidence of asthma exacerbations were similar in active treatment and placebo arms. Based on these data and because all patients are at least on a maintenance treatment with a stable dose of an anti-inflammatory medication (ICS) and as a basic rule they can keep their general treatments which are previously prescribed by patient's treating physician, a 'placebo' (i.e. no tiotropium on top of ICS as a controller medication) treatment group in this trial could be considered safe. Moreover, all patients will be provided with so-called rescue medication (open-label salbutamol HFA MDI provided by the sponsor) during the trial.

The potential benefit for the patients includes an improvement in the pulmonary function testing. Close observation and monitoring during the study is expected to lead to an improved asthma control of the participating patients.

Proprietary confidential information.
© 2012 Boehringer Ingelheim International GmbH or one or more of its affiliated companies. All rights reserved.
This document may not - in full or in part - be passed on, reproduced, published or otherwise used without prior written permission.

3. DESCRIPTION OF DESIGN AND TRIAL POPULATION

3.1 OVERALL TRIAL DESIGN AND PLAN

This is a randomised, double-blind, placebo-controlled, parallel-group trial to evaluate the safety and efficacy of 2.5 and 5 µg of tiotropium inhalation solution delivered by the Respimat inhaler (once daily) compared with placebo over 52 weeks in the patients with moderate to severe persistent asthma who were treated with a medium dose of ICS (Appendix 10.3).

After signing informed consent at Visit 0 and an initial screening visit at Visit 1, patients will enter a 4-week screening period. Patients who meet all inclusion and none of the exclusion criteria will be randomised into the 52-week treatment period in which they will receive either 2.5 µg tiotropium (2 actuations of 1.25 µg) once daily, 5 µg tiotropium (2 actuations of 2.5 µg) once daily or placebo in a double-blind fashion. Patients will be evaluated for an additional 3 weeks following completion of the randomised treatment period. Visits 0, 3, 3A, 4A/B, 5A/B, 6A/B/C, and 8 may be conducted during business hours. Visits 1 to 7 except Visit 3, 3A, 4A/B, 5A/B, and 6A/B/C will always start in the evening.

Patients who withdraw prematurely from the randomised treatment period will be followed up regarding their vital status. They will be contacted at their predicted normal exit date from the trial, i.e. completion of the 52-week treatment period plus 3-week follow-up period.

For an overview plan of procedures please see to the Flow Chart and see the figure below.

AEs will be documented throughout the trial, i.e. starting with informed consent and ending 3 weeks after last administration of study medication in case no follow-up is necessary.

All trial relevant documentation will be stored by BI in the clinical trial master file (CTMF). Trial relevant documentation for the trial sites will be filed in the Investigator Site File (ISF) at the investigator sites.

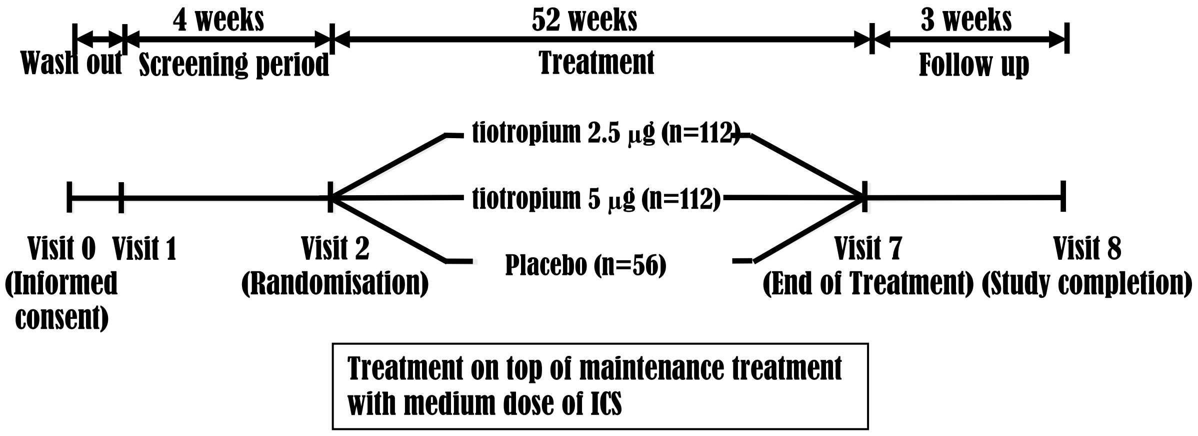

Figure 3.1:1 Trial Design

Proprietary confidential information.
© 2012 Boehringer Ingelheim International GmbH or one or more of its affiliated companies. All rights reserved.
This document may not - in full or in part - be passed on, reproduced, published or otherwise used without prior written permission.

3.1.1 Administrative structure of the trial

Sponsor:
The trial is sponsored by Nippon Boehringer Ingelheim Co., Ltd. (NBI).

NBI will appoint a trial clinical monitor (TCM), responsible for writing and preparing the Core documents for the conduct of the trial [clinical trial protocol (CTP), core consent, etc.], for directing the clinical trial team in the preparation, conduct and reporting of the trial, for ordering clinical trial supplies and materials necessary for the trial, for ensuring appropriate information and training of clinical monitor local (CML)/clinical research associates (CRAs) /investigators of participating sites, for ensuring timely cleaning of data, database lock and delivery of results and for writing the clinical trial report (CTR) or overseeing preparation of associated publications.

Data management and statistical evaluation will be done by NBI according to BI standard operating procedures (SOPs). For these activities, a trial data manager and a trial statistician will be appointed.

Tasks and functions assigned in order to organise, manage, and evaluate the trial will be defined according to BI SOPs. A list of responsible persons will be given in the CTMF document. It will be also given in the protocol attachments.

The organisation of the trial will be done by NBI or a by a contract research organisation (CRO) with which the responsibilities and tasks have been agreed and a written contract has been filed before initiation of the clinical trial. In NBI, a CML will be appointed responsible for coordinating the activities required in order to manage the trial in accordance with applicable regulations and internal SOPs in Japan.

Coordinating investigator:
A Coordinating investigator will be nominated to coordinate investigators at different sites participating in this multicentre trial. Tasks and responsibilities for the Coordinating investigator will be defined in a contract.

Documents on participating (principal) investigators and other important participants, especially their curricula vitae, will be filed in the CTMF.

Data safety monitoring board (DSMB):
A DSMB will not be implemented on trial level, but might be implemented on project level. If so, safety review meetings will be held as per separate DSMB charter.

Interactive voice response system (IVRS) and interactive web response system (IWRS):
An IVRS and an IWRS will be used for randomisation to a treatment group in this trial and for appropriate re-supply of medication to patients. The ability to unblind will be available to the investigators via the IVRS/IWRS.

Proprietary confidential information.
© 2012 Boehringer Ingelheim International GmbH or one or more of its affiliated companies. All rights reserved.
This document may not - in full or in part - be passed on, reproduced, published or otherwise used without prior written permission.

CROs:
1) CRO for provision Asthma Monitor® 3 (AM3) devices (Spirometry measurement and 12-lead ECG will be performed with a site-equipment).
2) Central laboratory for clinical lab sample measurement and logistics
3) CRO for a service organization that provides support to the field monitoring in the form of outsourced CRAs

All contracts and relevant meeting minutes will be stored by BI in the CTMF.

Details on handling of the trial supplies including responsible institutions are given in chapter 4 of this protocol.

The ISF document will be kept in print-out version at the sites as far as required by local regulation and BI-SOP. A copy of the ISF documents will be kept as an electronic CTMF document according to BI SOPs.

3.2 DISCUSSION OF TRIAL DESIGN, INCLUDING THE CHOICE OF CONTROL GROUP

The trial design has been selected to evaluate the safety and efficacy of two doses of tiotropium in patients with moderate to severe persistent asthma that is not fully controlled although the patients are treated with a medium dose of ICS. However, this study is not designed to detect any difference of the two tiotropium doses against placebo in terms of overall or any specific AE incidence. It is designed to assess safety reducing possible bias against tiotropium-specific AEs by placing a placebo group. The comparisons of tiotropium versus placebo are not part of an inferential analysis.

In two completed Phase II proof-of-concept trials (BI Trial Nos. 205.341 and 205.342) no untoward events happened to patients treated with placebo and the overall incidence of AEs and the incidence of asthma exacerbations were similar in active treatment and placebo arms. Based on these data and because all patients are at least on a maintenance treatment with a stable dose of an anti-inflammatory medication (ICS) and as a basic rule they can keep their general treatments which are previously prescribed by patient’s treating physician, a 'placebo' (i.e. no tiotropium on top of ICS as a controller medication) treatment group in this trial could be considered safe. Moreover, all patients will be provided with so-called rescue medication (open-label salbutamol HFA MDI provided by the sponsor).

Washout requirements prior to pulmonary function testing and other medication restrictions (see Section 4.2.2) are given to reduce possible influences on pulmonary function testing and ensure patient's safety during the trial. The permitted concomitant asthma medication (see Section 4.2) should be kept stable during the complete trial period with the exception of acute treatment of asthma exacerbations.

Proprietary confidential information.
© 2012 Boehringer Ingelheim International GmbH or one or more of its affiliated companies. All rights reserved.
This document may not - in full or in part - be passed on, reproduced, published or otherwise used without prior written permission.

3.3 SELECTION OF TRIAL POPULATION

A sufficient number of outpatients of either sex with a diagnosis of moderate to severe persistent asthma will be enrolled in the study to ensure approximately 280 patients in about 60 sites at maximum (preferably at least 10 patients per site) are entered (randomised) in the trial. Additional sites may be initiated to ensure sponsor's timelines. Randomisation will end when the TCM has determined that enough number of patients for evaluation is randomised even though some patients are in screening period..

Every effort should be made to keep patients in the trial until they complete all trial procedures. Patients who discontinue after randomisation should not be re-enrolled at a later date. A record will be kept of all patients who fail to complete all trial visits and their reason for discontinuation.

A log of all patients included into the study (i.e. having given informed consent) will be maintained in the ISF at the investigational site irrespective of whether they have been treated with the investigational drug or not.

3.3.1 Main diagnosis for study entry

Outpatients with a history of asthma and a current diagnosis of moderate to severe persistent asthma according to Global Initiative for Asthma (GINA) guideline and who are symptomatic (partly controlled) despite their current maintenance treatment with a medium dose of ICS are eligible for inclusion if they fulfil all the inclusion criteria (Section 3.3.2) and none of the exclusion criteria (Section 3.3.3).

3.3.2 Inclusion criteria

1. All patients including the patients under age (under 20 years old) must sign and date an Informed Consent Form consistent with ICH-GCP guidelines and Good Clinical Practice (GCP) prior to participation in the trial [i.e. prior to any trial procedures, including any pre-trial washout of medications and medication restrictions for pulmonary function test (PFT) at Visit 1]. Regarding patients under age, a guardian or a legally authorised representative must also sign and date an Informed Consent Form.

2. Male or female outpatients aged at least 18 years but not more than 75 years at Visit 0.

3. All patients must have at least a 12-week history of asthma at the time of enrolment (Visit 0) into the trial. The diagnosis should be confirmed at Visit 1 by fulfilling inclusion criterion 5.

4. The initial diagnosis of asthma must have been made before the patient's age of 40.

5. The diagnosis of asthma has to be confirmed at Visit 1 with a bronchodilator reversibility (15-30 minutes after 400 µg salbutamol) resulting in a FEV_1 increase of ≥12% and ≥200 mL (see Appendix 10.3).

   NOTE: If the patient is not eligible due to the predefined reversibility at Visit 1, the patient's reversibility test can be repeated only once within 2 weeks (see Section 6.1). Patient must fulfill all spirometry criteria (incl. pre-bronchodilator values) at the repeated lung function test, which excludes ACQ.

Proprietary confidential information.
© 2012 Boehringer Ingelheim International GmbH or one or more of its affiliated companies. All rights reserved.
This document may not - in full or in part - be passed on, reproduced, published or otherwise used without prior written permission.

6. All patients must have been on maintenance treatment with a medium, stable dose of ICS (see Appendix 10.3) (alone or in a fixed combination with a LABA) for at least 4 weeks prior to Visit 1.

7. All patients must be symptomatic at Visit 1 (screening) and prior to randomisation at Visit 2 as defined by an ACQ (see Appendix 10.4) mean score of ≥1.5.

   NOTE: If the patient is not eligible due to the predefined score at Visit 1, the patient should not be further evaluated. If the patient is not eligible due to the predefined score at Visit 2, the patient's Visit 2 can be repeated only once for further assessment (see Section 6.1).

8. All patients must have a pre-bronchodilator FEV_1 ≥60% and ≤90% of predicted normal at Visit 1.

   Predicted normal values will be calculated according to European Community for Steel and Coal (ECSC) (R94-1408) (see Appendix 10.3).

9. Patients must be never-smokers or ex-smokers who stopped smoking at least one year (52 weeks) prior to enrolment (Visit 0) and who have a smoking history of less than 10 pack years (see Appendix 10.3 for calculation).

10. Patients must be able to use the Respimat inhaler (Appendix 10.1) correctly, which is judged at the discretion of the investigator..

11. Patients must be able to perform all trial related procedures including technically acceptable PFTs and use of electronic diary (eDiary)/peak flow meter, which is judged at the discretion of the investigator.

3.3.3 Exclusion criteria

1. Patients with a significant disease other than asthma.

   A significant disease is defined as a disease which, in the opinion of the investigator, may (i) put the patient at risk because of participation in the trial, or (ii) influence the results of the trial, or (iii) cause concern regarding the patient's ability to participate in the trial.

2. Patients with a clinically relevant abnormal screening (Visit 1) haematology or blood chemistry if the abnormality defines a significant disease as defined in exclusion criterion no 1.

3. Patients with a recent history (i.e. 6 months or less) of myocardial infarction prior to Visit 0.

4. Patients who have been hospitalised for cardiac failure during the past year prior to Visit 0.

5. Patients with any unstable or life-threatening cardiac arrhythmia or cardiac arrhythmia requiring intervention or a change in drug therapy within the past year prior to Visit 0.

6. Patients with lung diseases other than asthma (e.g. COPD).

7. Patients with known active tuberculosis.

Proprietary confidential information.
© 2012 Boehringer Ingelheim International GmbH or one or more of its affiliated companies. All rights reserved.
This document may not - in full or in part - be passed on, reproduced, published or otherwise used without prior written permission.

8. Patients with malignancy and/or patients who have undergone resection, radiation therapy or chemotherapy for malignancy within the last 5 years prior to Visit 0. Patients with treated basal cell carcinoma are allowed.

9. Patients who have undergone thoracotomy with pulmonary resection. Patients with a history of thoracotomy for other reasons should be evaluated as per exclusion criterion no. 1.

10. Patients with significant alcohol or drug abuse, which is judged at the discretion of the investigator, within the past 2 years prior to Visit 0.

11. Patients with known hypersensitivity to anticholinergic drugs, BAC, EDTA, or any other components of the study medication delivery systems.

12. Pregnant or nursing women.

13. Women of childbearing potential not using a highly effective method of birth control.

    Highly effective methods of birth control are defined as those which result in a low failure rate (i.e. less than 1% per year) when used consistently and correctly. Female patients will be considered to be of childbearing potential unless surgically sterilised by hysterectomy or bilateral tubal ligation/salpingectomy, or post-menopausal for at least 2 years.

14. Patients who have taken an investigational drug within 4 weeks prior to Visit 1.

15. Patients who have been treated with beta-blocker medication
    - within 4 weeks prior to Visit 1 and/or
    - during the screening period (period between Visit 1 and Visit 2).

    Topical beta-blocker eye medications for non-narrow angle glaucoma are allowed.

16. Patients who have been treated with long-acting anticholinergic tiotropium (Spiriva)
    - within 4 weeks prior to Visit 1 and/or
    - during the screening period (period between Visit 1 and Visit 2).

17. Patients who have been treated with oral beta-adrenergics
    - within 4 weeks prior to Visit 1 and/or
    - during the Screening period (period between Visit 1 and Visit 2)

18. Patients who have been treated with systemic corticosteroids
    - within 4 weeks prior to Visit 1 and/or
    - during the screening period (period between Visit 1 and Visit 2).

19. Patients who have been treated with anti-immunoglobulin E (IgE) antibodies, e.g. omalizumab (Xolair®),
    - within 6 months prior to Visit 1 and/or
    - during the screening period (period between Visit 1 and Visit 2).

Proprietary confidential information.
© 2012 Boehringer Ingelheim International GmbH or one or more of its affiliated companies. All rights reserved.
This document may not - in full or in part - be passed on, reproduced, published or otherwise used without prior written permission.

20. Patients who have been treated with other non-approved and according to international guidelines not recommended 'experimental' drugs for routine asthma therapy [e.g. tumor necrosis factor (TNF)-alpha blockers, methotrexate, cyclosporine]
    - within 4 weeks prior to Visit 1 and/or
    - during the screening period (period between Visit 1 and Visit 2).

21. Patients with any asthma exacerbation or any respiratory tract infection
    - in the 4 weeks prior to Visit 1 and/or
    - during the screening period (period between Visit 1 to Visit 2).

    Visit 1 and/or Visit 2 should be postponed in case of an asthma exacerbation or respiratory tract infection. Refer to Section 6.1 for information on re-scheduling of visits.

22. Patients who are currently participating in another trial.

23. Patients with narrow-angle glaucoma and/or micturition disorder due to prostatic hyperplasia.

24. Patients with below 80% of the eDiary completion compliance on Visit 2 (diary compliance of at least 80% is required; refer to Section 6.1 for instructions).
    If on Visit 2, the eDiary completion compliance is below 80%, rescheduling of Visit 2 is required. Rescheduling of Visit 2 (for 2 weeks) is allowed twice.

Precautionary statement

Tiotropium

As an anticholinergic drug, tiotropium may potentially worsen symptoms and signs associated with narrow-angle glaucoma, prostatic hyperplasia or bladder-neck obstruction.

As tiotropium is a predominantly renally excreted drug, patients with moderate to severe renal impairment (creatinine clearance ≤50 mL/min) treated with tiotropium should be monitored closely.

3.3.4 Removal of patients from therapy or assessments

3.3.4.1 Removal of individual patients

An individual patient is to be withdrawn from the trial if any of the following criteria apply:

- The patient withdraws consent, without the need to justify the decision.
- The patient is no longer able to participate for medical reasons (e.g. pregnancy, surgery, AEs, or other diseases).
- Decision by BI to discontinue a specific patient for his/her safety (e.g. in case of SAEs).

Withdrawal from the trial of an individual patient may be considered if any of the following criteria apply:

Intercurrent illness or an AE, which requires discontinuation of treatment per protocol. Investigators should check carefully if this applies for patients who experience any of the following criteria:

- More than 3 courses of systemic corticosteroids are required to treat asthma exacerbations.

Proprietary confidential information.
© 2012 Boehringer Ingelheim International GmbH or one or more of its affiliated companies. All rights reserved.
This document may not - in full or in part - be passed on, reproduced, published or otherwise used without prior written permission.

- Twelve or more actuations of rescue medication (salbutamol HFA MDI) per day are used for more than 2 consecutive days (use of 12 or more actuations of rescue medication for at least 2 consecutive days will be alerted by the AM3)
- A drop of patient's pre-bronchodilator FEV_1 (clinic assessment) below 40% predicted.
- A decrease of patient's best morning PEF of ≥40% from the patient's mean morning PEF for more than 2 consecutive days (a decrease of ≥30% for at least 2 consecutive days will be alerted by the AM3).

  During the screening period, patient's mean morning PEF is defined as the mean value of all best morning PEF values obtained during the first 7 days after Visit 1.

  During the treatment period, patient's mean morning PEF is defined as the mean value of all best morning PEF values obtained during the complete screening period including the morning of Visit 2.

Data of patients who discontinue or withdraw prior to randomisation will be entered in the trial database and will be listed. Data of patients who discontinue or withdraw after randomisation must be documented and the reason for withdrawal must be recorded in the electronic case report form (eCRF). The data must be included in the trial database and must be reported.

Refer to Section 6.2.3 for procedures to be followed for patients prematurely terminating the trial.

Pregnancy

If a patient becomes pregnant during the trial the investigational product needs to be stopped and the patient should be followed up until birth or otherwise termination of the pregnancy. The data of the patient will be collected and reported in the CTR until the patient's last visit and any events thereafter will be reported in the BI drug safety database. Refer to Section 5.2.2.2 for detailed information on event reporting in case of pregnancy.

3.3.4.2 Discontinuation of the trial by the sponsor

BI reserves the right to discontinue the trial overall or at a particular trial site at any time for the following reasons:

1. Failure to meet expected enrolment goals overall or at a particular trial site,

2. Emergence of any efficacy/safety information that could significantly affect continuation of the trial,

3. Violation of GCP, the CTP, or the contract by a trial site or investigator, disturbing the appropriate conduct of the trial.

The investigator/the trial site will be reimbursed for reasonable expenses incurred in case of trial termination (except in case of the third reason).

Proprietary confidential information.
© 2012 Boehringer Ingelheim International GmbH or one or more of its affiliated companies. All rights reserved.
This document may not - in full or in part - be passed on, reproduced, published or otherwise used without prior written permission.

4. TREATMENTS

4.1 TREATMENTS TO BE ADMINISTERED

Patients will be randomised 2:2:1 to 2.5 µg tiotropium inhalation solution, 5 µg tiotropium inhalation solution, or placebo over the 52-week treatment period. The double-blind study is realised by the use of matching placebos. During the treatment period the patients inhale two actuations from the Respimat inhaler (tiotropium or placebo) every evening.

Patients randomised to 2.5 µg tiotropium inhale the following medication
In the evening: two actuations of tiotropium from the 1.25 µg Respimat inhaler.

Patients randomised to 5 µg tiotropium inhale the following medication
In the evening: two actuations of tiotropium from the 2.5 µg Respimat inhaler.

Patients randomised to placebo inhale the following medication
In the evening: two actuations from the tiotropium placebo Respimat inhaler.

NBI will supply the investigational product.

4.1.1 Identity of BI investigational product and comparator product

Investigational product - 2.5 µg tiotropium bromide

Substance (INN):	tiotropium bromide
Pharmaceutical form:	inhalation solution
Unit strength:	2.5 µg (1.25 µg per actuation) delivered dose ex mouthpiece
Device:	Respimat inhaler
Posology:	2 actuations once daily (in the evening)
Route of administration:	oral inhalation

Investigational product - 5 µg tiotropium bromide

Substance (INN):	tiotropium bromide
Pharmaceutical form:	inhalation solution
Unit strength:	5 µg (2.5 µg per actuation) delivered dose ex mouthpiece
Device:	Respimat inhaler
Posology:	2 actuations once daily (in the evening)
Route of administration:	oral inhalation

Placebo - inhalation solution

Substance (INN):	-
Pharmaceutical form:	inhalation solution
Unit strength:	Not applicable
Device:	Respimat inhaler
Posology:	2 actuations once daily (in the evening)
Route of administration:	oral inhalation

Instruction for use of the Respimat is provided in Appendix 10.1.

Proprietary confidential information.
© 2012 Boehringer Ingelheim International GmbH or one or more of its affiliated companies. All rights reserved.
This document may not - in full or in part - be passed on, reproduced, published or otherwise used without prior written permission.

4.1.2 Method of assigning patients to treatment groups

When a patient is qualified for entry into the randomised treatment period, treatment assignment will be made by means of a third-party phone/web-based randomisation on Visit 2. This will involve the use of an IVRS/IWRS. To facilitate the use of an IVRS/IWRS, the investigator will receive an IVRS/IWRS worksheet for each patient with the complete IVRS/IWRS dialogue and all necessary instructions for using the IVRS/IWRS.

Upon signing informed consent, patients will be assigned a unique patient number. At each visit when study medication is dispensed (Visits 2, 3, 4, 5, and 6) the IVRS/IWRS will assign medication numbers to each patient. Refer to Section 4.1.6 for details on packaging and labelling,

Details on the IVRS/IWRS system are provided in the ISF.

4.1.3 Selection of doses in the trial

Two completed Phase II proof-of-concept trials (BI Trial Nos. 205.341 and 205.342) provide evidence that the 5 µg dose of Spiriva Respimat is effective in severe persistent asthma on top of ICS-LABA and provide clinically effective bronchodilation in patients with severe and moderate persistent asthma. BI Trial No. 205.341 also evaluated the 10 µg dose and both active doses 5 and 10 µg established the therapeutic plateau for the higher dosing level. Moreover, both active doses were shown to be safe and well tolerated, however, considering the overall risk and benefit evaluation of two doses, the 5 µg dose was selected for further evaluation in Phase III trials. The lower dose of 2.5 µg has been added to the Phase III trials (BI Trial Nos. 205.418 and 205.419), which are ongoing in patients including Japanese with moderate persistent asthma, to assess whether a lower dose may still offer sufficient response. In these trials the actual severity of asthma in patients is “moderate to severe” in a precise sense even the above term indicates only “moderate”. In this local trial (BI Trial No. 205.464), since the final dose has not been decided yet, the lower 2 doses of 2.5 µg and 5 µg will be administered to assess long term (52 weeks) safety and efficacy in Japanese patients with moderate to severe persistent asthma, which are the same severity of asthma as the mentioned Phase III trials. The selection of evening administration in this patient group was mainly to consider nocturnal control of airway patency.

4.1.4 Drug assignment and administration of doses for each patient

Dispensing of study medication

Patients will be randomised at Visit 2 to one of the three treatment groups. Study medication will be dispensed to the patient by the investigator/pharmacist at Visits 2 to 6. The amount of study medication dispensed will be recorded on the drug accountability forms.

Priming of the Respimat inhaler

Each newly assembled Respimat inhaler has to be primed. The inhaler should be primed by actuating it until an aerosol is visible plus 3 additional actuations. All priming actuations should be directed to the ground.

Proprietary confidential information.
© 2012 Boehringer Ingelheim International GmbH or one or more of its affiliated companies. All rights reserved.
This document may not - in full or in part - be passed on, reproduced, published or otherwise used without prior written permission.

Once assembled, the shelf-life of the Respimat is 3 months (study medication, reserve medication, and training devices). Therefore it is important to ALWAYS enter the date of first priming on the medication label of the Respimat immediately after first priming.

Instructing the patient

Detailed instructions and training for the use of the Respimat inhaler will be given to the patient at Visits 1 and 2 (see Appendix 10.1). Patients should NOT inhale from a training device on Visit 2. At Visit 2, after the patient returns the training device to the site and study medication will be dispensed to the patient by the investigator/pharmacist. At all subsequent visits (Visits 3 to 7) the investigator or qualified study personnel will train the inhalation procedure and will reinforce a correct inhalation technique.

Patients will be instructed to contact the site that they need to use their reserve inhaler and the site will document this.

Patients will be instructed at each visit to retain and return all used and unused medication and devices at the subsequent visit.

Patients who couldn't take a dose within the specified time frame should be instructed as a basic rule to take the next dose at the next scheduled time. However, patients who couldn't take a dose within the specified time frame can take their medication with the following rule even if it applies a protocol deviation.

Specified time frame for study medication: between 17:00 and 20:00 (± 30 minutes of evening administration time at Visit 2)

- Evening dose including study medication: until 24:00

Study medication administration at clinic visits (Visits 2, 4, 5, 6, and 7)

Patients will be instructed to withhold their evening dose of study medication and the evening dose of their usual ICS (if regular medication/schedule for the patient) and their leukotriene modifier (LTRA) (if applicable) on the day of clinic visits. The administration of the evening doses of ICS, LTRA (if applicable) and study medication should be done in the clinic only after the pre-dose procedures (which include AM3 PEF/FEV_1 measurement and eDiary, questionnaires, vital signs and PFT).

At each clinic visit during the treatment period medication administration will be conducted in a fixed sequence [1. ICS (if regular medication/schedule for the patient) and LTRA (if applicable), 2. from Respimat] because time point zero for PFTs is defined as the complete time of inhalation (i.e. end time of the second inhalation from Respimat). Patient's ICS should be administered without change in posology (b.i.d., q.d., AM, PM), i.e. as previously prescribed by patient's treating physician. Patient should inhale from the Respimat immediately after the inhalation of the patient's own ICS medication. The patient should be in a seated position under the direct supervision of the investigator or his/her delegate.

Proprietary confidential information.
© 2012 Boehringer Ingelheim International GmbH or one or more of its affiliated companies. All rights reserved.
This document may not - in full or in part - be passed on, reproduced, published or otherwise used without prior written permission.

Study medication will be administered in the evening between 17:00 and 20:00 (± 30 minutes of the evening administration time at Visit 2) at all visits except Visits 3, 3A, 4A/B, 5A/B, and 6A/B/C during the treatment period (at Visits 3, 3A, 4A/B, 5A/B, and 6A/B/C, the patient can take his/her study medication at home).

On Visit 2 the start time of the inhalation and the end time of the second inhalation from the Respimat will be recorded on the eCRF. From Visits 4, 5, 6, and 7 the end time of the inhalation from the Respimat on one day prior to visit day will be recorded on the eCRF because the end time of the second inhalation of study medication from Respimat is defined as time point zero for PFTs. A Visit card will be provided to the patients to record the end time of inhalation at home on one day prior to visit day of Visits 4, 5, 6, and 7.

Study medication administration at home

Patients will self-administer the evening doses of study medication between clinic visits and will record the administration of each dose of study medication in the eDiary.

The evening dose of the patient's own ICS, LTRA (if applicable) and study medication should be administered between 17:00 and 20:00 (± 30 minutes of evening administration time at Visit 2. It is recommended that medication is taken immediately after the eDiary questions have been answered and the PEF measurements have been performed.

Respimat Return

Patients should return all dispensed study medication (including training, reserve and rescue medication) to the clinic at all visits.

Study medication dispensed at Visit 2, will be used for the study medication administration at Visit 2 and will be returned at Visit 3.

Study medication dispensed at Visit 3, will be used for the study medication administration on the day of Visit 3 and will be returned at Visit 4.

Study medication dispensed at Visit 4, will be used for the study medication administration at Visit 4 and will be returned at Visit 5.

Study medication dispensed at Visit 5, will be used for the study medication administration at Visit 5 and will be returned at Visit 6.

Study medication dispensed at Visit 6, will be used for the study medication administration at Visit 6 and Visit 7 and will be returned at Visit 7.

No study medication will be dispensed at Visit 7.

Any Respimat inhaler that has been reported as malfunctioning by a patient or investigator will be returned to the Department of Drug Delivery, Boehringer Ingelheim Pharma GmbH & Co. KG (Germany), for investigation. A detail of the procedure for the return of used inhalers with malfunction is provided in Appendix 10.2.

Proprietary confidential information.
© 2012 Boehringer Ingelheim International GmbH or one or more of its affiliated companies. All rights reserved.
This document may not - in full or in part - be passed on, reproduced, published or otherwise used without prior written permission.

See Section 4.1.8 for details regarding drug accountability requirements.

4.1.5 Blinding and procedures for unblinding

4.1.5.1 Blinding

Patients, investigators and everyone involved in analysing or with an interest in this double-blind study will remain blinded with regard to the randomised treatment assignments until after database lock. The randomisation code will be kept secret by clinical trial support up to database lock.

BI will generate the randomisation schedule, and prepare and code the medication in a blinded fashion. Trial supplies will be assigned to the patients via IVRS/IWRS.

Refer to Section 4.1.5.2 for rules of breaking the code for an individual or for all patients in emergency situations.

4.1.5.2 Procedures for emergency unblinding

The ability to unblind will be available to the investigator via the IVRS/IWRS. Unblinding must only be used in emergency situations when the identity of the study drug must be known by the investigator to provide appropriate medical treatment or if required to assure safety of trial participants. Each site receives a manual from the IVRS/IWRS provider that contains instructions on how to unblind the treatment of a patient via the IVRS/IWRS (via 24-hour Emergency helpline). In case, emergency unblinding is conducted, the site must contact NBI and write the reasons for unblinding and the initial of the person who unblinds the code and the date of unblinding in eCRF or worksheet. If possible, the CML and TCM must be contacted prior to the site unblinding a patient's treatment. Patients unblinded to treatment will be withdrawn from the trial.

4.1.6 Packaging, labelling, and re-supply

All study medication will be contained in individual patient treatment kit**s** identified with the trial number and a specific medication number. Each patient treatment kits will contain one Respimat inhaler and one drug-filled cartridge and each kit will contain sufficient medication for 30 days of treatment.

The Respimat inhaler will lock after 60 actuations have been administered and will no longer actuate any medication.

The kits will have a two-part tear-off label. One part of each tear-off label will remain on the kit box, and the other part will be attached to a special drug dispensing log which will be part of the ISF. Examples of the labels are provided in the ISF.

The investigator or designee should fill out the following information on the medication label of Respimat prior to dispensing the medication to the patient when he/she assembles and primes the first device:

- trial site's name (should be entered on the medication label of kit at time of dispense)
- date of first priming (should be entered at time of the first priming only for the first device)

Proprietary confidential information.
© 2012 Boehringer Ingelheim International GmbH or one or more of its affiliated companies. All rights reserved.
This document may not - in full or in part - be passed on, reproduced, published or otherwise used without prior written permission.

For details of packaging and the description of the label, see the ISF.

Medication dispensing

The allocation of treatment boxes dispensed at each visit will be handled by an IVRS/IWRS.

At Visit 2, patients will be dispensed one Respimat treatment kit (one Respimat inhaler and one drug-filled cartridge) and one Respimat kit for reserve medication. When a new reserve medication is dispensed, site staff will attach a label with "Reserve". This is to allow the patient the flexibility of not having to return to the clinic immediately to replace a lost Respimat inhaler. The reserve Respimat and drug-filled cartridge should NOT be assembled prior to leaving the clinic. The patient must assemble and prime the reserve device at home if needed.

At Visit 3, patients will be dispensed two Respimat treatment kits. The second Respimat and drug-filled cartridge in the Respimat treatment kit should NOT be assembled prior to leaving the clinic. The patient must assemble and prime this device at home after the previous cartridge is emptied and he/she should fill out the following information on the medication label when he/she assembles and primes the device:

- date of first priming (should be entered by the patient at time of first priming).

At Visits 4 and 5, patients will be dispensed three Respimat treatment kits. The second and the third Respimat and drug-filled cartridges in each Respimat treatment kit should NOT be assembled prior to leaving the clinic. The patient must assemble and prime this device at home after the previous cartridge is emptied and he/she should fill out the following information on the medication label when he/she assembles and primes the device:

- date of first priming (should be entered by the patient at time of first priming).

At Visits 6, patients will be dispensed four Respimat treatment kits. The second, the third, and the fourth Respimat and drug-filled cartridges in each Respimat treatment kit should NOT be assembled prior to leaving the clinic. The patient must assemble and prime this device at home after the previous cartridge is emptied and he/she should fill out the following information on the medication label when he/she assembles and primes the device:

- date of first priming (should be entered by the patient at time of first priming).

Additional four Respimat treatment kits for reserve medication are available at the investigational site for issuing on an as needed basis. Patients should always have a reserve Respimat treatment kit in their possession. At each visit, site staff should assess whether the reserve medication can be re-dispensed to the patient (considering remaining actuations and shelf-life) or if dispense of a new (replacement) reserve Respimat inhaler plus drug-filled cartridge is needed.
When a new reserve medication is dispensed, site staff will attach a label with "Reserve". New reserve Respimats and drug-filled cartridges should NOT be assembled prior to leaving the clinic. The patient must assemble and prime the reserve device at home if needed. Allocation of new reserve medication boxes will be handled by the IVRS/IWRS system.

The minimum amount of kits provided by the IVRS/IWRS for each patient at each visit is shown in Table 4.1.6: 1. In total, 18 kits are prepared for one patient at maximum, such as 13 for usual treatment and 5 reserve medications (a reserve medication prepared for each 3 months at maximum).

Proprietary confidential information.
© 2012 Boehringer Ingelheim International GmbH or one or more of its affiliated companies. All rights reserved.
This document may not - in full or in part - be passed on, reproduced, published or otherwise used without prior written permission.

Table 4.1.6: 1 Dispensing of medication by the IVRS/IWRS

Study Period	Screening	Treatment Period					
Visit	1	2	3	4	5	6	7
Study week	-4	0	4	12	24	36	52
Study day	-28	1	29	85	169	253	365
Dispense medication		X	X	X	X	X	
Quantity of respimat	0	2*	2	3	3	4	0
Termination of study medication							X

* 1 study medication and 1 study medication for a reserve medication are provided at Visit 2. In case the reserve medication is primed or expires etc, an additional medication will be provided as a new reserve medication for the patient.

Open-label supplies

BI will provide the following open-label supplies:

- Respimat inhalers, placebo cartridges for training purposes. A new training Respimat should be used for each patient. The training Respimat can be used until 3 months after priming or until the device is empty. The date of first priming should be entered on the medication label of the Respimat.
- Salbutamol HFA MDI inhalation aerosol (100 µg per actuation) for use as rescue medication during screening, treatment and follow-up periods (Visit 0 to Visit 8). It will also be used for reversibility testing to confirm eligibility of a patient as inlusion criteria 5. Salbutamol HFA MDI will be dispensed to the patient at clinic visits as needed.

4.1.7 Storage conditions

All clinical trial supplies must be stored in a locked, secure cabinet and must be kept in their original packaging under the recommended storage conditions and may only be dispensed to trial subjects according to protocol. Trial supplies should not be frozen.

A temperature log must be maintained at the site. If the storage conditions are found to be outside the specified range, immediately contact the CML.

Further details are provided in the IB and on the labels, a sample of which will be part of the ISF.

4.1.8 Drug accountability

Drug supplies, which will be provided by the sponsor and/or a CRO appointed by the sponsor, must be kept in a secure, limited access storage area under the storage conditions defined by the sponsor. Where necessary, a temperature log must be maintained to make certain that the drug supplies are stored at the correct temperature.

The investigational drug storage manager will receive the investigational drugs delivered by the sponsor when the following requirements are fulfilled:

- approval of the study protocol by the institutional review board (IRB),

Proprietary confidential information.
© 2012 Boehringer Ingelheim International GmbH or one or more of its affiliated companies. All rights reserved.
This document may not - in full or in part - be passed on, reproduced, published or otherwise used without prior written permission.

- availability of a signed and dated clinical trial contract between the sponsor and the head of trial centre,
- approval/notification of the regulatory authority, e.g. competent authority,
- availability of the curriculum vitae of the principal investigator,
- availability of a signed and dated CTP or immediately imminent signing of the CTP.

The investigational drug storage manager must maintain records of the product’s delivery to the trial site, the inventory at the site, the use by each patient, and the return to the sponsor or alternative disposition of unused products.

These records will include dates, quantities, batch/serial numbers, expiry (‘use by’) dates, and the unique code numbers assigned to the investigational products and trial patients. The investigational drug storage manager will maintain records that document adequately that the patients were provided the doses specified by the CTP and reconcile all investigational products received from the sponsor.

The investigational drug storage manager should return the unused and collected investigational drugs (including empty boxes) to the sponsor after unblinding the trial. In case investigational drugs are returned before unblinding of the trial, the investigational drug storage manager should seal the opened box (excluding empty boxes) for the patient, and before returning the unused and collected investigational drugs (including empty boxes) to the sponsor.When returning the investigational drugs, the investigational drug storage manager should exercise utmost caution to assure that the sponsor and other relevant trial staff members remain blinded to the patient's name on the package (box or label) of the investigational drugs.

At the time of return to the sponsor and/or appointed CRO, the investigational drug storage manager must verify that all unused or partially used drug supplies have been returned by the patient and that no remaining supplies are in the investigator’s possession.

Upon completion of the trial, the investigational drug storage manager submits to the sponsor a copy of the investigational drug dispensing and return log. When submitting the copy, the investigational drug storage manager should exercise caution to assure that the sponsor and other relevant trial staff members remain blinded to the patient's name.

Rescue medication in this trial (salbutamol HFA MDI) is considered a non-investigational medicinal product. Source data documentation and full drug accountability in regard to dispensed and returned medication to investigational site and to patients are required.

4.2 CONCOMITANT THERAPY, RESTRICTIONS, AND RESCUE TREATMENT

The investigator must record all medication used by the patient in the 12 weeks prior to Visit 0 and throughout the trial on the Concomitant Therapy eCRF.

Proprietary confidential information.
© 2012 Boehringer Ingelheim International GmbH or one or more of its affiliated companies. All rights reserved.
This document may not - in full or in part - be passed on, reproduced, published or otherwise used without prior written permission.

4.2.1 Rescue medication, emergency procedures, and additional treatments

4.2.1.1 Rescue medication

Administration of rescue medication is allowed at any point during the trial. Open-label salbutamol HFA MDI (100 μg per actuation) will be provided as rescue medication (non-investigational medicinal product). During the complete trial period including screening, treatment and follow-up period, only salbutamol HFA MDI provided by BI is allowed for as occasion requires (PRN) rescue medication use. Formoterol, alone or in fixed combinations with an ICS such as Symbicort® (budesonide and formoterol), is not allowed as rescue medication in this trial. During the screening and treatment period, patients must record the number of inhalations (actuations) of rescue medication used during the daytime and the night time in their eDiary.

If rescue medication is administered on a visit day within 8 hours prior to the pre-dose PFT, the visit will be re-scheduled once. Further rescheduling should be discussed with the CML.

4.2.1.2 Emergency procedures

There are no special emergency procedures to be followed.

4.2.1.3 Additional treatments

Medications allowed to control acute asthma exacerbations as medically necessary during the screening, treatment and follow-up period:

1. PRN salbutamol HFA inhalation aerosol (MDI) provided by BI and to be recorded in the patient's eDiary.

2. Temporary addition of systemic corticosteroids is allowed during the study period, which is judged at the discretion of the investigator. Pulmonary function testing should not occur within 4 weeks of the last administered dose of the addition (see Section 6.1 for visit schedule).

3. Temporary increases in the dose of ICS are allowed during the study period, which is judged at the discretion of the investigator. Pulmonary function testing should not occur within 3 weeks of the last administered dose of an increase (see Section 6.1 for visit schedule).

4. Temporary addition of theophylline preparations is allowed during the study period, which is judged at the discretion of the investigator. Pulmonary function testing should not occur within one week of the last administered dose of an increase or addition (see Section 6.1 for visit schedule).

5. The use of antibiotics is not restricted and may be used as medically necessary for asthma exacerbations and/or other infections. Pulmonary function testing should not occur within 4 days of the last administered dose of an increase or addition of antibiotics if given for an asthma exacerbation or respiratory tract infection (see Section 6.1 for visit schedule).

Proprietary confidential information.
© 2012 Boehringer Ingelheim International GmbH or one or more of its affiliated companies. All rights reserved.
This document may not - in full or in part - be passed on, reproduced, published or otherwise used without prior written permission.

The treatment of asthma exacerbations including initiation of systemic corticosteroids should be done according to the investigator´s or treating physician´s medical judgement and should be in line with national and international recommendations. In the case of life-threatening exacerbations, any and all therapies deemed medically necessary can be prescribed.

Medications allowed prior to and throughout the trial:

1. Maintenance treatment with a medium dose of ICS (required for study entry; refer to inclusion criterion no. 6).

2. Inhaled LABAs, patch beta-adrenergics, cromone, antihistamines, anti-allergic drugs, methylxanthines, mucolytic agents not containing bronchodilators and LTRAs (if stabilised for at least 4 weeks prior to the trial and remains stable throughout the trial).

3. Any orally inhaled rapid-acting beta-adrenergic agent is allowed prior to Visit 0. During the screening and randomised treatment periods and during the follow-up period, only salbutamol HFA MDI provided by BI is allowed for PRN rescue medication use. The washout requirements before clinic visits need to be followed.

4. Oral beta-adrenergics and beta blockers may be re-introduced during the follow-up period. However, it is not allowed to start with oral beta-adrenergics and beta blockers if not already prescribed prior to study entry. Treatment with pulmonary medications should remain stabilised as far as possible throughout the trial period.

Refer to Section 4.2.2.1 for washout periods prior to pulmonary function testing during the study (including Visit 1).

4.2.2 Restrictions

4.2.2.1 Restrictions regarding concomitant treatment

The following table provides an overview of required, permitted and restricted medication.

Proprietary confidential information.
© 2012 Boehringer Ingelheim International GmbH or one or more of its affiliated companies. All rights reserved.
This document may not - in full or in part - be passed on, reproduced, published or otherwise used without prior written permission.

Table 4.2.2.1: 1 Overview of required, permitted and restricted medication

Medications prescribed for asthma may be washed out appropriately after Visit 0 (after signing informed consent) to comply with the criteria in the table below.

Drug Class	Sub-class	Prior to Visit 1	Visit 1 and Screening Period	Treatment Period	Follow up Period
Corticosteroids	ICS	REQUIRED<br>Patients must have been on maintenance treatment with a medium, stable dose for at least 4 weeks prior to Visit 1	REQUIRED Maintenance treatment with a medium, stable dose<br>Temporary addition to treat exacerbations is allowed.[1]	REQUIRED Maintenance treatment with a medium, stable dose<br>Temporary addition to treat exacerbations is allowed.[1]	REQUIRED Maintenance treatment with a medium, stable dose
	Systemic corticosteroids	NOT permitted for at least 4 weeks prior to Visit 1	NOT permitted<br>Temporary addition to treat exacerbations is allowed.[1]	NOT permitted<br>Temporary addition to treat exacerbations is allowed. [1]	Permitted

Proprietary confidential information.
© 2012 Boehringer Ingelheim International GmbH or one or more of its affiliated companies. All rights reserved.
This document may not - in full or in part - be passed on, reproduced, published or otherwise used without prior written permission.

Table 4.2.2.1: 1(cont'd) Overview of required, permitted and restricted medication

Drug Class	Sub-class	Prior to Visit 1	Visit 1 and Screening Period	Treatment Period	Follow up Period
Beta-adrenergics/ Beta-blockers	Inhaled short-acting beta-adrenergics (SABAs)	Permitted	Rescue<br>(prior to PFTs at least 8-hour washout)	Rescue<br>(prior to PFTs at least 8-hour washout)	Rescue
	Inhaled LABAs and patch beta-adrenergics	Permitted<br>To be stabilised for 4 weeks prior to Visit 1 and throughout the trial.	Permitted<br>(prior to PFTs at least 24-hour washout)	Permitted<br>(prior to PFTs at least 24-hour washout)	Permitted
	Oral beta-adrenergics	NOT permitted for at least 4 weeks prior to Visit 1	NOT permitted	NOT permitted	Permitted<br>(only re-introduction is allowed. NOT allowed to start if not used prior to trial entry)
	Beta blockers	NOT permitted for at least 4 weeks prior to Visit 1<br>Topical beta-blocker eye medications for treatment of non-narrow angle glaucoma are allowed.	NOT permitted<br>Topical beta-blocker eye medications for treatment of non-narrow angle glaucoma are allowed.	NOT permitted<br>Topical beta-blocker eye medications for treatment of non-narrow angle glaucoma are allowed.	Permitted<br>(only re-introduction is allowed. NOT allowed to start if not used prior to trial entry)
Anticholinergics	Short-acting anticholinergics<br>(inhalation aerosol and nasal spray)	Permitted	NOT permitted from 8 hours prior to Visit 1	NOT permitted	Permitted
	Long-acting anticholinergics	NOT permitted for at least 4 weeks prior to Visit 1	NOT permitted	Study medication	NOT permitted

Proprietary confidential information.
© 2012 Boehringer Ingelheim International GmbH or one or more of its affiliated companies. All rights reserved.
This document may not - in full or in part - be passed on, reproduced, published or otherwise used without prior written permission.

Table 4.2.2.1: 1(cont'd) Overview of required, permitted and restricted medication

Drug Class	Sub-class	Prior to Visit 1	Visit 1 and Screening Period	Treatment Period	Follow up Period
Miscellaneous	Other investigational drugs	NOT permitted for at least 4 weeks prior to Visit 1	NOT permitted	NOT permitted	NOT permitted
	Combination ICS/LABA (e.g. Adoair® Symbicort®)	Permitted<br>Combination ICS/LABA should be switched to the ICS mono-product without changing the steroid dose at least 24 hours prior to PFTs.<br>To be stabilised for 4 weeks prior to Visit 1 and throughout the trial.	Permitted<br>(prior to PFTs at least 24-hour washout)	Permitted<br>(prior to PFTs at least 24-hour washout)	Permitted
	Cromone	Permitted<br>To be stabilised for 4 weeks prior to Visit 1 and throughout the trial.	Permitted	Permitted	Permitted
	Antihistamines and anti-allergic drugs	Permitted<br>To be stabilised for 4 weeks prior to Visit 1 and throughout the trial.	Permitted	Permitted	Permitted
	Methylxanthines	Permitted<br>To be stabilised for 4 weeks prior to Visit 1 and throughout the trial.	Permitted Temporary addition of theophylline to treat exacerbations is allowed[1]	Permitted Temporary addition of theophylline to treat exacerbations is allowed[1]	Permitted

Proprietary confidential information.
© 2012 Boehringer Ingelheim International GmbH or one or more of its affiliated companies. All rights reserved.
This document may not - in full or in part - be passed on, reproduced, published or otherwise used without prior written permission.

Table 4.2.2.1: 1(cont'd) Overview of required, permitted and restricted medication

Drug Class	Sub-class	Prior to Visit 1	Visit 1 and Screening Period	Treatment Period	Follow up Period
	Mucolytics	Permitted<br>To be stabilised for 4 weeks prior to Visit 1 and throughout the trial.	Permitted	Permitted	Permitted
	LTRAs	Permitted<br>To be stabilised for 4 weeks prior to Visit 1 and throughout the trial.	Permitted	Permitted	Permitted
	Anti-IgE treatment (e.g. Omalizumab)	NOT permitted for at least 6 months prior to Visit 1	NOT permitted	NOT permitted	Permitted
	´Experimental´, non-approved asthma medications (e.g TNF-alpha blockers)	NOT permitted for at least 4 weeks prior to Visit 1	NOT permitted	NOT permitted	NOT permitted

1 Refer to Section 4.2.1.3 for washout period prior to PFTs in case of treatment of an asthma exacerbation.

Medication restrictions for pulmonary function testing (including Visit 1):

1. At least a 24-hour washout of LABA bronchodilators and patch beta-adrenergic bronchodilators prior to PFTs including Visit 1.

2. At least a 24-hour washout of combination products, ICS/LABA prior to PFTs including Visit 1.
   Combination products, ICS/LABA should be switched to ICS mono-product without changing the steroid dose at least 24 hours prior to PFTs.

3. At least an 8-hour washout of SABA bronchodilators prior to PFTs.

4. At least an 8-hour washout of short-acting anticholinergic bronchodilators prior to Visit 1 (not allowed between Visit 1 and 7).

Proprietary confidential information.
© 2012 Boehringer Ingelheim International GmbH or one or more of its affiliated companies. All rights reserved.
This document may not - in full or in part - be passed on, reproduced, published or otherwise used without prior written permission.

5. At least a 12-hour washout of short-acting methylxanthines prior to PFTs
6. At least a 24-hour washout of long-acting methylxanthines prior to PFTs
7. On a visit day (except Visit 3, 4A/B, 5A/B, and 6A/B/C), the evening doses of the patient's regular ICS therapy, LTRA (if applicable) and study medication should be taken after the visit day pre-dose PFT (i.e. at the clinic and not at home).

4.2.2.2 Restrictions on diet and life style

Restrictions prior to PFT visits

1. Medication washout restrictions should be adhered to as described in Section 4.2.2.1.
2. The patient must arrive at the building where the pulmonary function testing is performed at least 10 minutes prior to the start of each test.
3. On PFT days (including Visit 1), patients must refrain from strenuous activity for at least 12 hours prior to pulmonary function testing. Patients should also avoid cold temperatures, environmental smoke, dust or areas with strong odours (e.g. perfumes).
4. Coffee, tea, chocolate, cola and other caffeine-containing beverages and foods, and ice-cold beverages are not allowed at least 2 hours prior to the pulmonary function testing at clinic visits. Decaffeinated beverages are acceptable.
5. If a patient (re-)starts smoking during the trial, smoking should be discouraged for the 12 hours prior to pulmonary function testing until the end of all planned testing and will not be permitted in the 30-minute period prior to spirometry.

4.3 TREATMENT COMPLIANCE

The patient will complete an eDiary confirming that study medication has been taken and indicating the number of actuations of salbutamol HFA MDI use. The investigator will review these records with the patient at each visit (Visits 2 to 7) to assess treatment compliance. Compliance should be emphasised with a goal of at least 80% compliance rate. However, randomised patients will not be discontinued for lack of compliance without prior discussion with the CML.

On visit days, compliance will be guaranteed by administration of the trial drug under supervision of the investigating physician or designee.

Each patient will be trained in the correct use of the Respimat inhaler at Visit 1 and Visit 2.

Proprietary confidential information.
© 2012 Boehringer Ingelheim International GmbH or one or more of its affiliated companies. All rights reserved.
This document may not - in full or in part - be passed on, reproduced, published or otherwise used without prior written permission.

5. VARIABLES AND THEIR ASSESSMENT

5.1 EFFICACY

5.1.1 Endpoints of efficacy

5.1.1.1 Primary endpoints

In this trial, no primary endpoints of efficacy are defined because the primary objective of this trial is to evaluate the long term safety of tiotropium delivered via the Respimat inhaler in the patients with asthma.

5.1.1.2 Secondary endpoints

1. Trough FEV_1 response and trough forced vital capacity (FVC) at all visits except Visit 3, 3A, 4A/B, 5A/B, and 6A/B/C of the 52-week treatment period.

   Trough FEV_1 is defined as the FEV_1 measured (in the evening) at the -10 minute time point at the end of the dosing interval (24 hours post drug administration). Trough FEV_1 response is defined as the change from baseline in trough FEV_1.

   Baseline is the pre-treatment FEV_1 measured at Visit 2 in the evening 10 minutes prior to the evening dose of the patient's usual ICS controller medication (if regular medication/schedule for the patient) and first dose of study medication.

   Trough FVC and baseline FVC are defined in the same way as FEV_1.

2. Individual in-clinic PEF measurements during the 52-week treatment period.

3. PEF variability: PEF variability is the absolute difference between morning and evening PEF value divided by the mean of these two values (weekly means will be compared) during the 52-week treatment period.

4. PEF AM/PM: change from baseline in mean weekly morning and pre-dose evening PEF respectively measured by patients at home during the 52-week treatment period. Baseline is defined as the last week prior to randomisation.

5. Use of PRN salbutamol HFA MDI rescue medication during the 52-week treatment period: number of actuations of rescue therapy used per day (i.e. the full 24 hour period, the daytime and the night time; weekly means will be compared).

6. Asthma symptoms as assessed by the patient's eDiary during the 52-week treatment period. Analysis with regard to daytime and nocturnal symptoms will be done.

5.1.1.3 Other endpoints

1. FEV_1 AM/PM: mean morning and pre-dose evening FEV_1 respectively measured by patients at home (weekly means will be compared) from baseline to the last week of treatment. Baseline is defined as the last week prior to randomisation.

Proprietary confidential information.
© 2012 Boehringer Ingelheim International GmbH or one or more of its affiliated companies. All rights reserved.
This document may not - in full or in part - be passed on, reproduced, published or otherwise used without prior written permission.

2. The responder as assessed by the ACQ determined at Visit 5 (after 24 weeks from first administration of study medication) and at Visit 7 (the end of the 52-week treatment period).

   The following definition for responder will be used. A patient is said to be a responder if for that patient an improvement of at least 0.5 for the ACQ was observed. The minimum clinically important difference (MCID) for the ACQ is 0.5 (R09-1589).

5.1.2 Assessment of efficacy

PFT on study visits

Spirometers with a site-equipment will be used for the in-clinic spirometry measurements. The spirometers and their use, including daily calibration on patients' visit days, must meet American Thoracic Society (ATS)/European Respiratory Society (ERS) criteria (P05-12782). Spirometry will be conducted with the patient in a seated position having abstained from medications as specified in Section 4.2.2.1, and it is preferable that the same trained individual performs the PFTs for a given patient. The best of three efforts will be defined as the highest FEV_1, the highest FVC and the highest PEF each obtained on any of three blows meeting the ATS criteria (with a maximum of five attempts). The highest FEV_1, FVC and PEF will be selected regardless of whether they come from different spirometric manoeuvres or from the same manoeuvre. The highest FEV_1, FVC and PEF on study visits will be entered in the eCRF manually.

For each patient, pulmonary function testing will always start at approximately the same time of the day and depending on the time of dosing. At Visit 1 pulmonary function testing will be performed between 17:00 and 20:00. At Visits 2 to 7, visits and PFTs will be scheduled to enable dosing between 17:00 and 20:00. At Visits 4 to 7 except Visits 4A/B, 5A/B, and 6A/B/C pulmonary function testing should start with ± 30 minutes at maximum difference between the start of the tests on Visit 2 and the tests conducted on subsequent test days. The end of the second inhalation of the evening dose of study medication from the Respimat will be regarded as time point zero for pulmonary function testing.

At Visits 2 to 7 except Visit 3, 3A, 4A/B, 5A/B, and 6A/B/C, the 10 minute pre-dose measurement will be obtained in the period from 25 minutes to 5 minutes prior to the evening dose of ICS and study medication.

If a patient is unable to complete the PFTs during a visit, the CML should be notified as soon as possible. The eCRF will be completed indicating the reason for stopping testing. Refer to Section 4.2.1.1 for more details on conduction of trial procedures if rescue medication was administered during a visit day. Patients who are unable to complete the trial visit may leave the clinic only upon instruction from the supervising physician.

Refer to Section 4.1.4 (Study medication at clinic visits) for more information on medication intake times. Refer to Section 4.2.2.1 for restrictions regarding concomitant therapy prior to PFTs. Refer to Section 4.2.2.2 for restrictions on diet and life style prior to PFTs. Refer to the Flow Chart for the time schedule.

Proprietary confidential information.
© 2012 Boehringer Ingelheim International GmbH or one or more of its affiliated companies. All rights reserved.
This document may not - in full or in part - be passed on, reproduced, published or otherwise used without prior written permission.

Asthma Control Questionnaire (ACQ)

The ACQ developed by Elizabeth Juniper (R00-1157) has 6 patient self-administered questions for the time period of the last week prior to the visit and one question concerning pre-bronchodilator FEV_1 to be completed by a member of the clinic staff. Each question has a 7-point scale. The ACQ will be completed at Visits 1, 2, 5 and 7 and should be completed prior to any discussion with a health professional (physician, nurse or study coordinator). Question 7 will be completed after pulmonary function testing. The questions in the questionnaire are weighted equally, the score is the mean of the responses to all 7 questions.
The ACQ is provided on paper. Patients should be by themselves in a quiet place when they complete the questionnaire. The investigator (or designated site personnel) should check that all items have been completed by the patient, but the response to each item should not be questioned. The responses of ACQ will be entered in the eCRF manually.

Please refer to Appendix 10.4 for an example of the questionnaire.

Electronic peak flow meter with eDiary (AM3)

The patients will receive an AM3 (EResearch Technology GmbH, Hoechberg, Germany) which combines the features of an electronic peak flow meter (measurement of both PEF and FEV_1) and an eDiary in one device.

Patients will receive the AM3 at Visit 1 and instructions for the use of the device at Visits 1 and 2. They will use the device during the screening and treatment period (Visits 1 to 7). The device will be used twice daily to first record questions related to asthma symptoms and quality of life (QOL), use of rescue salbutamol HFA MDI, use of study medication (during the treatment period only), and then to measure PEF and FEV_1. Morning and evening recordings and measurements should be performed at approximately the same time of the day (± 30 minutes) between 5:00 and 8:00 and 17:00 and 20:00, respectively.

The patient will be alerted by the AM3 to contact the investigator in case of one of the following situations:

- A decrease of patient's best morning PEF of ≥30% from the patient's mean morning PEF for at least 2 consecutive days

  During the screening period, patient's mean morning PEF is defined as the mean value of all best morning PEF values obtained during the first 7 days after Visit 1.

  During the treatment period, patient's mean morning PEF is defined as the mean value of all best morning PEF values obtained during the complete screening period including the morning of Visit 2.

- The patient used 12 or more actuations of rescue medication for at least 2 consecutive days

All PEF/FEV_1 and eDiary data saved in the AM3 will be downloaded at each visit after

Proprietary confidential information.
© 2012 Boehringer Ingelheim International GmbH or one or more of its affiliated companies. All rights reserved.
This document may not - in full or in part - be passed on, reproduced, published or otherwise used without prior written permission.

Visit 1 except Visits 3A, 4A/B, 5A/B, and 6A/B/C and transmitted to central data management of the vendor. At each trial visit the investigator receives a print-out of all downloaded AM3 data for review. Details and instructions for use are given in the Appendix 10.6 and the ISF.

eDiary at home

The diary part of the AM3 will be used to record the answers to the questions raised in the daily diary. The eDiary includes questions on asthma symptoms, QOL and number of actuations of rescue medication. The diary must be answered in the morning immediately upon arising (after the patient has cleared out mucus) and in the evening both prior to administration of maintenance ICS (if regular medication/schedule for the patient), LTRA (if applicable), study medication (only in the evening), and rescue medication (if needed). Study medication taken in the evening during the treatment period will also be recorded in the AM3.

Peak flow measurements at home

The patient will record twice-daily (morning and evening) PEF and FEV_1 during the screening and treatment period (Visits 1 to 7) with the AM3 immediately after answering the eDiary questions.

The morning measurement should be performed upon arising after the patient has cleared out mucus and prior to administration of maintenance ICS (if regular medication/schedule for the patient), LTRA (if applicable), and rescue medication (if needed). The evening measurement will be performed prior to administration of maintenance ICS (if regular medication/schedule for the patient), LTRA (if applicable), study medication,and rescue medication (if needed).

The patient should perform three PEF manoeuvres in the standing position with the AM3. All acceptable PEF and FEV_1 values are stored in the AM3 with date and time of the reading. The highest PEF and the highest FEV_1 out of up to three acceptable blows, but not necessarily from the same blow, will be used for evaluation.

Peak flow measurements and eDiary at Visits 2 to 7 except Visits 3, 3A, 4A/B, 5A/B, and 6A/B/C

The morning recordings at home should be done as usual.

In the afternoon/evening, patients should answer the evening questions of the eDiary and use the electronic peak flow meter in the clinic between -1:00 and -0:30 and prior to administration of maintenance ICS (if regular medication/schedule for the patient) LTRA (if applicable), and study medication. The medication should be administered at the clinic after the pre-dose pulmonary function testing with the spirometer in clinic.

Proprietary confidential information.
© 2012 Boehringer Ingelheim International GmbH or one or more of its affiliated companies. All rights reserved.
This document may not - in full or in part - be passed on, reproduced, published or otherwise used without prior written permission.

5.2 SAFETY

5.2.1 Endpoints of safety

1. All AEs.

2. Vital signs: pulse rate and blood pressure (seated) recorded not only in conjunction with spirometry but also all records performed at all visits during treatment period.

3. Laboratory data at all visits except Visits 2, 3A, 4A/B, 5A/B, and 6A/B/C during treatment period.

4. 12-lead electrocardiogram (ECG) at visit 7.

5. Vital status information collected for all randomised patients including prematurely discontinued patients on the originally planned follow up visit date (Visit 8). Regarding vital status of the patients who withdraw from the trial, please refer to Sections 6.2.3 and 8.1.

5.2.2 Assessment of adverse events

5.2.2.1 Definitions of adverse events

<u>AE</u>

AE is defined as any untoward medical occurrence, including an exacerbation of a pre-existing condition, in a patient in a clinical investigation who received a pharmaceutical product. The event does not necessarily have to have a causal relationship with this treatment.

<u>SAE</u>

A SAE is defined as any AE which results in death, is immediately life-threatening, results in persistent or significant disability/incapacity, requires or prolongs patient hospitalisation, is a congenital anomaly/birth defect, or is to be deemed serious for any other reason if it is an important medical event when based upon appropriate medical judgement which may jeopardise the patient and may require medical or surgical intervention to prevent one of the other outcomes listed in the above definitions.
An AE which possibly leads to disability will be reported as an SAE. Every new occurrence of cancer will be reported as a SAE regardless of the duration between discontinuation of the drug and the occurrence of the cancer.

<u>Significant events</u>

The following are considered as significant events:

- Hepatic injury defined by the following alterations of liver parameters: an elevation of AST and/or ALT ≥3 fold ULN combined with an elevation of total bilirubin ≥2 fold ULN measured in the same blood draw sample.

Significant events are to be reported in an expedited manner similar to SAEs, even if they do not meet any of the seriousness criteria (for details see Section 5.2.2.2).

Proprietary confidential information.
© 2012 Boehringer Ingelheim International GmbH or one or more of its affiliated companies. All rights reserved.
This document may not - in full or in part - be passed on, reproduced, published or otherwise used without prior written permission.

Intensity of AE

The intensity of the AE should be judged based on the following:

Mild: Awareness of sign(s) or symptom(s) which is/are easily tolerated
Moderate: Enough discomfort to cause interference with usual activity
Severe: Incapacitating or causing inability to work or to perform usual activities

Causal relationship of AE

Medical judgment should be used to determine the relationship, considering all relevant factors, including pattern of reaction, temporal relationship, de-challenge or re-challenge, confounding factors such as concomitant medication, concomitant diseases and relevant history. Assessment of causal relationship should be recorded in the case report forms. The reason for the decision on causal relationship needs to be provided in the case report form (CRF).

Yes: There is a reasonable causal relationship between the investigational product administered and the AE.
No: There is no reasonable causal relationship between the investigational product administered and the AE.

If a SAE is reported from a still blinded trial, the causal relationship must be provided by the investigator for all potential trial drugs, i.e. the BI trial drug and for all other trial drugs (i.e. any active comparator or placebo according to the trial design).

Worsening of underlying disease or other pre-existing conditions

Worsening of the underlying disease or of other pre-existing conditions will be recorded as an (S)AE in the (e)CRF.

Changes in vital signs, ECG, physical examination, and laboratory test results

Changes in vital signs, ECG, physical examination and laboratory test results will be recorded as an (S)AE in the (e)CRF , if they are judged clinically relevant by the investigator.

Asthma Exacerbations

The patient will document any worsening of asthma symptoms during the screening and treatment period in the electronic diary of the AM3 and measure the PEF twice daily (see Appendix 10.6). In addition, the patient will receive a paper patient diary card to document specific asthma symptoms, required medical treatment (e.g. change in asthma medication, need for medical care) or lost working days due to the asthma worsening (see Appendix 10.5). The investigator will review the patient's entries and enter the relevant information from the paper patient diary card in the eCRF. The paper patient diary card will remain at the site. A new patient diary card should be dispensed at every visit as needed. The investigator should collect all information regarding asthma exacerbations including review of the electronic and paper diary. Specific questions should be raised to capture any asthma worsening, any changes in concomitant asthma medication including the introduction of systemic corticosteroids or any unplanned need for medical care due to asthma or lost working days that have not been documented in the patient's diaries.

Proprietary confidential information.
© 2012 Boehringer Ingelheim International GmbH or one or more of its affiliated companies. All rights reserved.
This document may not - in full or in part - be passed on, reproduced, published or otherwise used without prior written permission.

It is the investigator's responsibility to report any deterioration of asthma as an AE regardless if the sponsor's definition of asthma exacerbations is fulfilled or not.

The treatment of asthma exacerbations including initiation of systemic corticosteroids should be done according to the investigator´s or treating physician´s medical judgement and should be in line with national and international recommendations. If systemic corticosteroids are required, the GINA guidelines recommend to initially dose oral glucocorticosteroids between 0.5 to 1 mg of prednisolone or equivalent /kg body weight during 24-hours (P10-03196). Whenever feasible, the following scheme is recommended for the trial: 30 mg/day prednisolone or prednisolone equivalent for 7 days.

The onset of an asthma exacerbation should be defined by the onset of the first worsened symptom respectively PEF deterioration. The end of an asthma exacerbation should be recorded as defined by the investigator. Courses of systemic corticosteroids that are separated by one week or more should be treated as separate exacerbations.

Refer to Appendix 10.7 for the BI definition of an asthma exacerbation in general and a severe asthma exacerbation.

5.2.2.2 Adverse event and serious adverse event reporting

All AEs, serious and non-serious, occurring during the course of the clinical trial (i.e., from signing the informed consent onwards through the observational phase) will be collected, documented and reported to the sponsor by the investigator on the appropriate CRFs/eCRFs/SAE reporting forms. Reporting will be done according to the specific definitions and instructions detailed in the 'AE Reporting' section of the ISF.

For each AE, the investigator will provide the onset date, end date, intensity, treatment required, outcome, seriousness, and action taken with the investigational drug. The investigator will determine the relationship of the investigational drug to all AEs as defined in Section 5.2.2.1.

The investigator also has the responsibility to report AEs occurring in a certain period (until 3 weeks after the last dose of study medication) after a patient completes the trial. Any AEs reported to the sponsor during this phase must be documented in the safety database.

If not stipulated differently in the ISF, the investigator must report the following events via telephone/fax using the SAE form immediately (within 24 hours or the next business day whichever is shorter) to the sponsor: SAEs and non-serious AEs occurring at the same time as an SAE and/or which are medically related to the SAE(s), and significant events. With receipt of any further information to these events, a follow-up SAE report has to be provided. SAEs and non-serious AEs must include a causal relationship assessment made by the investigator. This information must be also reported immediately to the head of the trial site.

BI has set up a list of AEs which are defined to be always serious. In order to support the investigator with the identification of these "always serious adverse events", if a non serious AE is identified to be serious per BI definition, a query will be raised. The investigator must verify the description and seriousness of the event. If the event description is correct, the item "serious" needs to be ticked and an SAE has to be reported in expedited fashion following the same procedure as above.

The list of these adverse events can be found via the Remote Data Capture (RDC) environment.

Proprietary confidential information.
© 2012 Boehringer Ingelheim International GmbH or one or more of its affiliated companies. All rights reserved.
This document may not - in full or in part - be passed on, reproduced, published or otherwise used without prior written permission.

The SAE form is to be forwarded to the defined unique entry point identified for the sponsor (contact details will be provided in the ISF). This immediate report is required irrespective of whether the investigational product has been administered or not and irrespective of causal relationship. It also applies if new information to existing SAEs or significant events becomes available.

Vital status information

After any premature withdrawal of patients that took at least one dose of study medication, the vital status information (dead or alive) will be collected on the originally planned visit date of the follow up visit (Visit 8). Any death during the vital status observation period needs to be reported as SAE by the investigator according to the BI SAE procedures.

Pregnancy

In rare cases, pregnancy might occur in clinical trials. Once a female subject has been enrolled into the clinical trial, after having taken study medication, the investigator must report immediately any drug exposure during pregnancy to the sponsor. Drug exposure during pregnancy has to be reported immediately (within 24 hours or next business day whichever is shorter) to the defined unique entry point for SAE forms of the sponsor (contact details will be provided in the ISF). The outcome of the pregnancy associated with the drug exposure during pregnancy must be followed up. In the absence of an (S)AE, only the Pregnancy Monitoring Form for Clinical Trials and not the SAE form is to be completed. The ISF will contain the Pregnancy Monitoring Form for Clinical Trials (Part A and Part B).

5.2.3 Assessment of safety laboratory parameters

Clinical laboratory testing

Clinical laboratory testing will be conducted on all patients at Visit 1 (to determine patient's eligibility) and at Visits 3, 4, 5, 6 and Visit 7 or at the withdrawal visit if the patient does not complete all trial visits. Lab parameters will be analysed by the central laboratory. Please refer to Appendix 10.8 for methodological details. Only at Visit 1, the white blood cell count, eosinophil count (absolute and relative), total serum IgE and creatinine levels will be recorded on the eCRF.

Haematology:

- Haemoglobin
- Haematocrit
- Absolute and relative eosinophil count (to be recorded in eCRF at Visit1)
- Red blood cell count
- White blood cell count (to be recorded in eCRF at Visit1) including differential test (neutrophils, lymphocytes, monocytes, eosinophils, basophils)
- Platelet count

Blood chemistry:

- Total serum IgE (to be recorded in eCRF at Visit1)
- lactate dehydrogenase (LDH)
- gamma-glutamyltransferase (γ-GT)
- serum glutamic oxaloacetic transaminase (SGOT) [alanine aminotransferase (AST)]
- serum glutamic pyruvic transaminase (SGPT) [aspartate aminotransferase (ALT)]

Proprietary confidential information.
© 2012 Boehringer Ingelheim International GmbH or one or more of its affiliated companies. All rights reserved.
This document may not - in full or in part - be passed on, reproduced, published or otherwise used without prior written permission.

- Calcium
- Inorganic phosphorus
- Creatinine (to be recorded in eCRF at Visit1)
- Potassium
- Sodium
- Chloride

All clinically significant findings at screening (Visit 1) will be recorded on the Medical History/Baseline Condition page in the eCRF.

New clinically significant findings or worsening of screening conditions detected during treatment period (or at a withdrawal visit) will be recorded as AEs on the appropriate eCRF page. An explanation of the aetiology of clinically significant abnormal findings must be made on the eCRF. All relevant abnormal findings have to be followed up until they have normalised or have been sufficiently characterised.

Pregnancy testing

A pregnancy test will be conducted at Visit 1 and Visit 7 (or at the withdrawal visit if the patient does not complete all trial visits) in all women of childbearing potential. It will be sufficient to use a urine test kit (provided by BI or by the investigator/hospital).

5.2.4 Electrocardiogram

A standard 12-lead ECG with a site-equipment will be performed on all patients at Visit 1 (to obtain information about the patient's baseline condition and to determine the patient's eligibility) and at Visit 7 or at the withdrawal visit if the patient does not complete all trial visits.

Measurements will be obtained after the patient lied down and rested for a minimum of 5 minutes. ECGs will be completed using local equipment. Clinical evaluation of ECGs is the responsibility of the Investigator.

All clinically significant findings at screening (Visit 1) will be recorded on the Medical History/Baseline Condition page in the eCRF.

New clinically significant findings or worsening of screening conditions detected during treatment period (or at a withdrawal visit) will be recorded as AEs on the appropriate eCRF page. An explanation of the aetiology of clinically significant abnormal findings must be made on the eCRF. All relevant abnormal findings have to be followed up until they have normalised or have been sufficiently characterised.

Proprietary confidential information.
© 2012 Boehringer Ingelheim International GmbH or one or more of its affiliated companies. All rights reserved.
This document may not - in full or in part - be passed on, reproduced, published or otherwise used without prior written permission.

5.2.5 Assessment of other safety parameters

Vital signs

Pulse rate, systolic and diastolic blood pressure will be measured and recorded in conjunction with pulmonary function testing at Visits 2 to 7 except Visits 3, 3A, 4A/B, 5A/B, and 6A/B/C and prior to inhalation of medication. Measurements will always be obtained immediately before pulmonary function testing with the patient seated and rested for a minimum of 5 minutes. The same person using the same blood pressure instrument on the same arm should perform all recordings.

All clinically significant findings at screening (Visit 1) will be recorded on the Medical History/Baseline Condition page in the eCRF.

New clinically significant findings or worsening of screening conditions detected during treatment period (or at a withdrawal visit) will be recorded as AEs on the appropriate eCRF page. An explanation of the aetiology of clinically significant abnormal findings must be made on the eCRF. All relevant abnormal findings have to be followed up until they have normalised or have been sufficiently characterised.

Physical examination

A complete, head-to-toe physical examination will be completed on all patients at the screening visit (Visit 1) and at Visits 2, 3, 4, 5, 6 and 7 or at the withdrawal visit if the patient does not complete all trial visits. The physical examination will also include measurements of systolic and diastolic blood pressure and pulse rate, which will be measured with the patient seated after having rested for at least 5 minutes.

All clinically significant findings at screening (Visit 1) will be recorded on the Medical History/Baseline Condition page in the eCRF.

New clinically significant findings or worsening of screening conditions detected during treatment period (or at a withdrawal visit) will be recorded as AEs on the appropriate eCRF page. An explanation of the aetiology of clinically significant abnormal physical findings must be made on the eCRF. All relevant abnormal physical findings have to be followed up until they have normalised or have been sufficiently characterised.

Vital status

Vital status information (dead or alive) will be collected at the originally planned visit date of the follow up visit (Visit 8) for discontinued patients following randomisation. The vital status eCRF will be completed. Collection of vital status information does not require a patient visit. If no information can be collected despite at least 3 (documented) phone calls and at least one registered letter have remained unanswered, the patient will be regarded as lost to follow-up. As for Patients who prematurely discontinue the study due to withdrawal of consent and agree to be contacted in order to obtain vital status information, they will be asked to cooperate for collecting vital status information (dead or alive) at the originally planned visit date of the follow up visit (Visit 8) for discontinued patients following randomisation.

Proprietary confidential information.
© 2012 Boehringer Ingelheim International GmbH or one or more of its affiliated companies. All rights reserved.
This document may not - in full or in part - be passed on, reproduced, published or otherwise used without prior written permission.

5.3 APPROPRIATENESS OF MEASUREMENTS

PFTs are a validated and well established measurement tool for lung function testing. PFTs will be conducted at clinic visits using the Spirometer of each site. FEV_1 is a standard measurement for the assessment of lung function.

The AM3 device (EResearch Technology GmbH, Hoechberg, Germany) will be used for measurement of PEF and FEV_1 and to record the data of the eDiary. The AM3 complies with the regulations of European Medical Device Directive and the Food and Drug Administration (FDA) guidelines in the United States.

The ACQ is a well-established, and validated questionnaire.

Proprietary confidential information.
© 2012 Boehringer Ingelheim International GmbH or one or more of its affiliated companies. All rights reserved.
This document may not - in full or in part - be passed on, reproduced, published or otherwise used without prior written permission.

6. INVESTIGATIONAL PLAN

6.1 VISIT SCHEDULE

This trial consists of a screening period, a treatment period and a follow-up period. Following the screening visit (Visit 1) and the 4-week screening period, patients will be randomised into the double-blind portion of the study (Visit 2). Patients will visit hospital every 4 weeks during a treatment period (Visits 2 to 7). Additional one visit will be scheduled 3 weeks after post-treatment (Visit 8).

Patients should make every attempt to complete the study as specified. Investigators should encourage patient treatment compliance and adherence to other protocol specific activities.

All deviations from the planned visit schedule will be documented.

Rescheduling in general

- A patient may be rescheduled twice (within 2 weeks of the scheduled visit date) due to lack of medication washout compliance.

Rescheduling prior to randomisation

- If on Visit 1, the inclusion criteria of a bronchodilator reversibility (15-30 minutes after 400 μg salbutamol) resulting in a FEV1 increase of ≥12% and ≥200 mL is NOT met, the reversibility test can be repeated once within 2 weeks. At the repeated reversibility test, the above criteria must be met; otherwise the patient is considered as non-eligible.

- The screening period (between Visits 1 and 2) may be extended by an additional 4 weeks as a basic rule for administrative reasons.

- If a patient experiences an asthma exacerbation or respiratory tract infection in the 4 weeks prior to Visit 1, the visit will be postponed until 4 weeks following recovery from the infection or exacerbation.

- If a patient experiences an asthma exacerbation or respiratory tract infection during the screening period (between Visits 1 and 2), randomisation will be postponed until 4 weeks following recovery from the infection or exacerbation.

- If the screening period is extended by more than an additional 4 weeks, but not more than an additional 8 weeks, the screening examination has to be repeated prior to randomisation. The repeat screening examination will include a physical examination, vital signs (blood pressure and pulse rate), 12-lead ECG and clinical laboratory evaluation (haematology, serum chemistry, and pregnancy test). The patient should return for these evaluations 2 weeks prior to the re-scheduled randomisation visit (Visit 2). All AEs and concomitant therapies will be recorded. If the screening period is to be extended more than an additional 8 weeks, the CML should be contacted.

Proprietary confidential information.
© 2012 Boehringer Ingelheim International GmbH or one or more of its affiliated companies. All rights reserved.
This document may not - in full or in part - be passed on, reproduced, published or otherwise used without prior written permission.

- If on Visit 2, the eDiary completion compliance is below 80%, rescheduling of Visit 2 is required. Patients may continue the trial if they show an eDiary completion compliance of at least 80% at the randomisation visit (Visit 2). Rescheduling of Visit 2 (for 2 weeks) is allowed twice.

  eDiary Completion Compliance is derived from the number of acceptable sessions. A session is either a set of morning data entries, or evening data entries. An acceptable session during the screening period is one in which at least 2 acceptable PEFs at the morning and evening sessions were stored and all diary data were entered. The calculation of the compliance during the screening period will be based on the last 10 days prior to Visit 2.

- If on Visit 2, the inclusion criteria of an ACQ mean score of ≥1.5 is NOT met, Visit 2 can be repeated once within 2 weeks. At the repeated Visit 2, the above criteria must be met; otherwise the patient is considered as non-eligible.

Refer to Section 4.2.1.3 for details on medications allowed to control acute asthma exacerbations and restrictions for these medications prior to PFTs.

Rescheduling after randomisation

- If rescheduling of visits after randomisation is necessary, the total daily doses of the Respimat inhaler (i.e. 30 days) need to be obeyed and the need to take reserve medication should be avoided. If possible an additional intermediate visit to dispense the new drug supply should be planned in order to avoid use of the reserve study medication. Refer to the Flow Chart for the rescheduling time windows.

- If rescue medication is administered during a visit day within 8 hours prior to the pre-dose PFT, the visit will be rescheduled once. Further rescheduling should be discussed with the CML.

- Subsequent visits should always be planned to take place at the originally scheduled dates to assure a 52-week treatment period and 3-week follow-up period.

Refer to Section 4.2.1.3 for details on medications allowed to control acute asthma exacerbations and restrictions for these medications prior to PFTs.

6.2 DETAILS OF TRIAL PROCEDURES AT SELECTED VISITS

6.2.1 Screening and run-in period

Informed Consent Visit (Visit 0)

- Informed Consent will be obtained prior to patient participation in the trial, which includes any medication washout procedures or restrictions. Upon obtaining Informed Consent, the patient will be instructed on the medication washout and other restrictions needed for the screening PFT at Visit 1. Refer to Section 4.2.2.1 for washout periods prior to pulmonary function testing at Visit 1. The interval between Visit 0 and Visit 1 may be between 1 and 28 days depending on medication washout requirements and restrictions.

Proprietary confidential information.
© 2012 Boehringer Ingelheim International GmbH or one or more of its affiliated companies. All rights reserved.
This document may not - in full or in part - be passed on, reproduced, published or otherwise used without prior written permission.

- The patient will receive directions on the as needed use of the salbutamol HFA MDI (as rescue medication) that will be dispensed at this visit.
- A preliminary check of in-/exclusion criteria is recommended at Visit 0 to avoid unnecessary washout procedures in non-eligible patients.

Observations and procedures at Visit 1

- Questions 1 to 6 of the ACQ questionnaire will be patient self-administered for assessment of degree of symptoms prior to any discussion with a health professional and prior to pulmonary function testing.
- Medication washout compliance will be verified.
- Demographic data (sex, race, date of birth, height, weight, duration of asthma, asthma background characteristics, pack years, smoking and employment status) will be recorded.
- Medical history regarding cardiovascular disorders, cerebrovascular accidents (CVAs), urinary/renal disorders/diseases, cancer and narrow-angle glaucoma will be recorded.
- Current conditions and conditions for which therapy is given in the last 12 weeks prior to Visit 1 as well as any chronic disease (excluding asthma) will be recorded (baseline conditions).
- Physical examination including vital signs (blood pressure and pulse rate) will be conducted and a 12-lead ECG will be performed. The vital signs measurement (seated) and ECG should be conducted following 5 minutes of rest and prior to the PFT measurements.
- All AEs experienced since signed Informed Consent will be reviewed and recorded.
- Concomitant therapy for the previous 12 weeks will be recorded.
- Blood samples will be collected and submitted to the central laboratory for haematology and serum chemistry. Blood samples need to be taken prior to the salbutamol dosing.
- A urine pregnancy test will be conducted (if applicable).
- Pulmonary function testing with the spirometer will be conducted in the evening between 17:00 and 20:00 immediately prior to (-10 min) and 15-30 minutes after the inhalation of 4 actuations of salbutamol. Question 7 of the ACQ questionnaire (regarding pre-bronchodilator FEV_1 predicted) will be completed by qualified site staff.
- Inclusion and exclusion criteria will be reviewed.
- Patients will be trained in the use of the Respimat inhaler.
- The patient's ability to perform technically acceptable PFTs and their ability to use the Respimat inhaler will be assessed.
- Patients qualified to enter the 4-week screening period of the trial will be issued
  - an electronic peak flow meter with integrated eDiary (AM3)
  - a paper patient diary card
  - additional rescue medication if needed
- Patients will receive training and instructions on
  - the use of the AM3 (including performance of PEFs and completing the eDiary)
  - the use of rescue medication
  - medication restrictions and washout requirements for the screening period and subsequent visits
  - returning all issued medication, the AM3 device and the paper patient diary card to the clinic on all subsequent visits.

Proprietary confidential information.
© 2012 Boehringer Ingelheim International GmbH or one or more of its affiliated companies. All rights reserved.
This document may not - in full or in part - be passed on, reproduced, published or otherwise used without prior written permission.

Screening Period

Patients who qualify on Visit 1 will measure twice-daily PEF and record their asthma symptoms and the number of actuations of rescue medication (daytime and nighttime) in the eDiary during the 4-week screening period.

If there is any indication during the screening period that the patient is not stable enough to complete the trial or that the patient is non-compliant with the study medication or restrictions, the patient should not be randomised. This evaluation should take place by the investigator after PEF and eDiary data saved in the AM3 have been downloaded and reviewed.

Details of any patient who is screened for the trial but is found to be ineligible must be entered in the Enrolment log and documented in the eCRF.

6.2.2 Treatment period

Observations and procedures at Visit 2

- At home, prior to the visit, the patient should answer the morning questions of the eDiary and use the electronic peak flow meter (AM3) as usual.
- Patients will answer the evening questions of the eDiary and use the electronic peak flow meter at the clinic.
- AM3 data will be downloaded and reviewed by the investigator. eDiary compliance should be reviewed (see inclusion criterion no 11, exclusion criterion no 24, and Section 6.1).
- Questions 1 to 6 of the ACQ questionnaire will be patient self-administered for assessment of degree of symptoms prior to any discussion with a health professional and prior to pulmonary function testing.
- Medication washout compliance will be verified.
- Patient's paper diary card will be collected and reviewed.
- AEs and changes in concomitant therapies will be recorded.
- Information regarding asthma exacerbations will be recorded.
- Patients will be trained in the use of the Respimat inhaler. Note: the patient should NOT inhale from a placebo inhaler at this visit.
- Pre-dosing PFT with the spirometer will be performed prior to inhalation of any medication. Question 7 of the ACQ questionnaire (regarding pre-bronchodilator FEV_1 predicted) will be completed by qualified site staff. Vital signs will be conducted in conjunction with the pre-dose PFT measurement.
- Inclusion and exclusion criteria will be reviewed to determine eligibility.
- Patients who meet all inclusion criteria and violate none of the exclusion criteria will be assigned to treatment according to the following procedure:

    1. Randomise patient using IVRS/IWRS.
    2. Allocate the appropriate medication kits using IVRS/IWRS.

- Patients will inhale medication in a fixed sequence as described in Section 4.1.4.

Proprietary confidential information.
© 2012 Boehringer Ingelheim International GmbH or one or more of its affiliated companies. All rights reserved.
This document may not - in full or in part - be passed on, reproduced, published or otherwise used without prior written permission.

- Patients will be issued
  - study medication including reserve medication
  - additional rescue medication if needed
  - a new paper patient diary card if needed
- Patients will receive training and instructions on
  - the use of the AM3 (including performance of PEFs and completing the eDiary)
  - the use of rescue medication
  - medication restrictions and washout requirements for the treatment period and subsequent visits
  - returning all issued medication, the AM3 device and the paper patient diary card to the clinic on all subsequent visits.

Observations and Procedures at Visits 3, 4, 5 and 6

- At home, prior to the visit, the patient should answer the morning questions of the eDiary and use the electronic peak flow meter (AM3) as usual.
- Patients will answer the evening questions of the eDiary and use the electronic peak flow meter at the clinic at all visits (except Visit 3).
- AM3 data will be downloaded and reviewed by the investigator.
- At Visit 5, questions 1 to 6 of the ACQ questionnaire will be patient self-administered for assessment of degree of symptoms prior to any discussion with a health professional and prior to pulmonary function testing.
- Medication washout compliance will be verified (except Visit 3).
- Patient's paper diary card will be collected and reviewed.
- AEs and changes in concomitant therapies will be recorded.
- Blood samples will be collected and submitted to the central laboratory for haematology and serum chemistry.
- Information regarding asthma exacerbations will be recorded.
- Study medication from the previous visit will be collected prior to study medication administration and new study medication will be dispensed. Allocate the appropriate medication kits (including new reserve medication if needed) using IVRS/IWRS.
- Pre-dosing PFT with the spirometer will be performed prior to inhalation of any medication. At Visit 5, question 7 of the ACQ questionnaire (regarding pre-bronchodilator FEV_1 predicted) will be completed by qualified site staff.
- Patients will inhale medication in a fixed sequence as described in Section 4.1.4.
- Physical examination including vital signs (blood pressure and pulse rate) will be conducted at the end of the visit after all PFT measurements.
- Patients will be issued
  - new study medication (including reserve medication if needed)
  - additional rescue medication if needed
  - a new paper patient diary card if needed
- Patients will receive instructions on
  - medication restrictions and washout requirements for the treatment period and subsequent visits
  - returning all issued medication, the AM3 device and the paper patient diary card to the clinic on all subsequent visits.

Proprietary confidential information.
© 2012 Boehringer Ingelheim International GmbH or one or more of its affiliated companies. All rights reserved.
This document may not - in full or in part - be passed on, reproduced, published or otherwise used without prior written permission.

Observations and Procedures at Visits 3A, 4A/B, 5A/B, and 6A/B/C

- AM3 data can be downloaded and reviewed by the investigator.
- Patient's paper diary card will be collected and reviewed.
- AEs and changes in concomitant therapies will be recorded.
- Information regarding asthma exacerbations will be recorded.
- Patients will be issued
    - reserve medication if needed
    - additional rescue medication if needed
    - a new paper patient diary card if needed
- Patients will receive instructions on
    - medication restrictions and washout requirements for the treatment period and subsequent visits
    - returning all issued medication, the AM3 device and the paper patient diary card to the clinic on all subsequent visits

6.2.3 End of trial and follow-up period

Observations and Procedures at Visit 7 (EoT)

- At home, prior to the visit, the patient should answer the morning questions of the eDiary and use the electronic peak flow meter (AM3) as usual.
- Patients will answer the evening questions of the eDiary and use the electronic peak flow meter in the clinic.
- AM3 data will be downloaded and reviewed by the investigator.
- Questions 1 to 6 of the ACQ questionnaire will be patient self-administered for assessment of degree of symptoms prior to any discussion with a health professional and prior to pulmonary function testing.
- Medication washout compliance will be verified.
- The AM3 will be collected.
- Patient's paper diary card will be collected and reviewed.
- AEs and changes in concomitant therapies will be recorded.
- Information regarding asthma exacerbations will be recorded.
- The patient's smoking status will be assessed.
- A 12-lead ECG will be recorded
- Blood samples will be collected and submitted to the central laboratory for haematology and serum chemistry.
- A urine pregnancy test will be conducted (if applicable).
- Study medication from the previous visit will be collected after study medication administration and no new study medication will be dispensed.
- Pre-dosing PFT with the spirometer will be performed prior to inhalation of any medication. Question 7 of the ACQ questionnaire (regarding pre-bronchodilator FEV_1 predicted) will be completed by qualified site staff.
- Patients will inhale medication in a fixed sequence as described in Section 4.1.4.
- Physical examination including vital signs (blood pressure and pulse rate) will be conducted at the end of the visit after all PFT measurements.

Proprietary confidential information.
© 2012 Boehringer Ingelheim International GmbH or one or more of its affiliated companies. All rights reserved.
This document may not - in full or in part - be passed on, reproduced, published or otherwise used without prior written permission.

- Patients will be issued additional rescue medication if needed.
- Patients will receive instructions for the follow-up period.

Observations and Procedures at Visit 8

The patient will visit the clinic 3 weeks after the last dose of study medication. Any AEs or changes in concomitant therapies that have occurred will be recorded in addition to the trial completion information. Any ongoing (serious) AEs should be followed until the event is resolved or there is a mutual agreement between the investigator and CML that follow-up is sufficient. Rescue medication will be collected.

Observations and Procedures in case of premature withdrawal

The following procedures should be performed after any premature withdrawal of patients that took at least one dose of study medication

- Physical examination including vital signs (blood pressure and pulse rate) will be conducted
- A 12-lead ECG will be recorded
- Blood samples will be collected and submitted to the central laboratory for haematology and serum chemistry.
- A urine pregnancy test will be conducted (if applicable).
- AEs and changes in concomitant therapies will be recorded. Any ongoing (serious) AEs should be followed until the event is resolved or there is a mutual agreement between the investigator and CML that follow-up is sufficient. All SAEs that occur within 3 weeks after a patient terminates study medication must be reported according to BI SAE procedures.
- Smoking status will be assessed.
- Study medication (used and unused) will be collected.
- AM3 data will be downloaded and reviewed by the investigator.
- The AM3 will be collected.
- Patient's paper diary card will be collected and reviewed.

The investigator should make every effort to perform the follow-up visit 3 weeks after the last dose of study medication on patients that withdrew prematurely.

Vital status information

After any premature withdrawal of patients that took at least one dose of study medication, the vital status information (dead or alive) will be collected on the originally planned visit date of the follow up visit (Visit 8). The vital status eCRFs will be completed. Collection of vital status information does not require a patient visit. Patients will be asked to consent to telephone follow-up at their normal exit date from the trial. As for Patients who prematurely discontinue the study due to withdrawal of consent and agree to be contacted in order to obtain vital status information, they will be asked to cooperate for collecting vital status information (dead or alive) at the originally planned visit date of the follow up visit (Visit 8) for discontinued patients following randomisation. If death occurs, the investigator will review the circumstances, including the relevant medical records to ascertain the most likely primary and secondary causes. Any death during the vital status observation period needs to be reported as SAE by the investigator according to the BI SAE procedures. Collection of vital status information will be performed in accordance with national ethical and regulatory guidelines.

Proprietary confidential information.
© 2012 Boehringer Ingelheim International GmbH or one or more of its affiliated companies. All rights reserved.
This document may not - in full or in part - be passed on, reproduced, published or otherwise used without prior written permission.

Withdrawal of Consent

In parallel with signing informed consent it will be confirmed if contacting at the originally planned visit date of the follow up visit (Visit 8) after the patient's withdrawal of consent in order to obtain vital status information can be agreed in the same informed consent. At the patient's prematurely termination due to withdrawal of consent, the patients should be asked to re-confirm that he/she (or someone he/she designates) agrees to be contacted by the investigators or site staff in the study team in order to obtain vital status information and this re-confirmation is documented in the patients' notes or the worksheet.

Proprietary confidential information.
© 2012 Boehringer Ingelheim International GmbH or one or more of its affiliated companies. All rights reserved.
This document may not - in full or in part - be passed on, reproduced, published or otherwise used without prior written permission.

7. STATISTICAL METHODS AND DETERMINATION OF SAMPLE SIZE

7.1 STATISTICAL DESIGN - MODEL

Design

This is a double-blind, placebo-controlled, multi-centre, parallel group study in which 52 weeks of randomised treatment are preceded by 4-week screening (baseline) period and followed by 3-week follow-up period. The primary objective is to evaluate the long-term safety of two doses (2.5 µg and 5 µg) of tiotropium Respimat in Japanese patients with asthma. According to the regulatory guidance, “sample size and exposure duration needed to assess the clinical safety of new drug on its clinical stage intended for long-term treatment of non-life-threatening conditions" as *Yakushin Notification No.592* issued from Ministry of Health, Labour and Welfare (MHLW) dated May 24, 1995, this study is not designed to detect any difference of the two tiotropium doses against placebo in terms of overall or any specific AE incidence. However it is designed to assess safety reducing possible bias against tiotropium - specific AEs by placing a placebo group from its design perspective. Thus, the comparisons of tiotropium versus placebo are not part of an inferential analysis. Safety will be evaluated descriptively on the basis of AEs, clinical laboratory tests, vital signs and 12-lead ECG.

Primary endpoint

No primary endpoints of efficacy are defined because the primary objective of this trial is to evaluate the long term safety with 2 doses of tiotropium delivered via the Respimat inhaler in the patients with asthma.

Secondary endpoints

For secondary endpoints and safety refer to Sections 5.1.1.2 and 5.2.1, respectively.

Baseline

For laboratory measurements the baseline will be the pre-dose measurements taken at Visit 1.

For all spirometry endpoints at clinic visits, the PFT in the evening of the randomisation visit (Visit 2), which is measured just prior to the evening dose of the patient’s usual ICS medication and first administration of the randomised treatment, is defined as baseline.

For all endpoints measured with the AM3 device at home, the average data obtained in the week immediately preceding Visit 2 will be used as baseline.

For ACQ, baseline is defined as the value obtained at the randomisation visit (Visit 2).

Response

Response is defined as the change from baseline.

Proprietary confidential information.
© 2012 Boehringer Ingelheim International GmbH or one or more of its affiliated companies. All rights reserved.
This document may not - in full or in part - be passed on, reproduced, published or otherwise used without prior written permission.

7.2 NULL AND ALTERNATIVE HYPOTHESES

The objective of this trial is to evaluate the safety of tiotropium (2.5 µg, 5 µg) delivered by the Respimat inhaler once daily for 52 weeks in asthma patients. Thus, it is not planned to test any statistical hypotheses against placebo with regard to any endpoints in a confirmatory sense. All p-values will be nominally interpreted and no multiplicity adjustments will be made.

7.3 PLANNED ANALYSES

The efficacy analyses and the summary of safety data will be based on all randomised patients that received at least one dose of study medication; this set of patients will be the treated set (TS). Full analysis set (FAS) includes all patients of the TS for which baseline and at least one post-baseline efficacy measurement are available. Clear definitions of each analysis set will be provided in the trial statistical analysis plan (TSAP).

7.3.1 Primary analyses

Analysis of safety based on the primary objective of this trial is described in Section 7.3.3.

7.3.2 Secondary analyses

Secondary analyses below will be performed on the FAS.

PFT Parameters

The PFT parameters of trough FEV_1 response and trough FVC response at clinical visits (12, 24, 36 and 52 weeks after the first administration) will be analysed using a restricted maximum likelihood (REML)-based mixed effect model with repeated measures (MMRM). Analyses will include the fixed, categorical effects of treatment, centre, visit and treatment by-visit interaction, as well as the continuous, fixed covariates of baseline value and baseline value-by-visit-interaction. A compound symmetry (co)variance structure will be used to model the within-patient errors. The Kenward-Roger approximation will be used to estimate denominator degrees of freedom. Analyses will be performed on least squares means using a two-sided alpha=0.05 (two-sided 95% confidence intervals). These endpoints are also summarised descriptively using observed cases. The mean pre-dose morning and evening PEF and FEV_1 , PEF variability measured with the AM3 device by the patients at home (weekly mean and overall mean) will be analysed using the same method as mentioned above.

Use of PRN salbutamol HFA MDI

The number of actuations rescue therapy used per day (i.e. daytime, night-time and the full 24 hour period) will be summarised in descriptive statistics.

Asthma symptoms

Nocturnal and daytime asthma symptoms as well as the number of asthma free days will be summarised in descriptive statistics.

Proprietary confidential information.
© 2012 Boehringer Ingelheim International GmbH or one or more of its affiliated companies. All rights reserved.
This document may not - in full or in part - be passed on, reproduced, published or otherwise used without prior written permission.

ACQ

ACQ endpoint (change from baseline) and ACQ responder will be summarised descriptively at each of clinic visits defined during the 52-week treatment period.

7.3.3 Safety analyses

Safety analyses will be performed on TS. In general, safety analyses will be descriptive in nature and will be based on BI standards. No hypothesis testing is planned prospectively. AEs will be coded using the MedDRA coding dictionary. Standard BI summary tables and listings will be produced to compare the incidence of AEs across the treatment groups. All events with an onset after the first dose of study medication up to a period of ~~3 weeks~~**30 days** after the last dose of study medication will be assigned to the treatment period for evaluation. Other AEs will be assigned either to the screening or post study period as appropriate. Laboratory data will be summarised descriptively based on BI standards. Vital signs will be also summarised descriptively. ECG will be tabulated as a part of medical history with respect to clinically significant findings.

7.3.4 Interim analyses

No interim analysis will be planned in this study.

7.4 HANDLING OF MISSING DATA

Every effort will be made to collect PFT data at the time points, except if the patient has used rescue medication. Post-baseline missing PFT values will be replaced with the least favourable PFT value if a patient withdraws due to worsening of asthma.

Randomly missing data after inhalation of study medication for which there are data from that visit both before and after the inhalation will be linearly interpolated. Randomly missing data with no subsequent non-missing values for that visit will be imputed using the last observation carried forward (LOCF) technique.

Data missing due to worsening of asthma or need of rescue medication will be replaced with the least favourable data for that visit (including pre-dose values).

For Patient Daily Record data, when the number of salbutamol HFA MDI doses is missing but other data are filled out on any given day then these data will be imputed by zero (because the presence of other data is interpreted as meaning that the patient was not having a problem).

Before calculating the baseline and treatment means, the following data will be excluded:

- data with a missing Patient Daily Record date,
- PEF data which is less than 50 L/min,
- duplicate data for the same date
- Daily Record data for days after drug was discontinued,
- Patient Daily Record data for the period during which systemic steroids or theophylline doses were increased because of an exacerbation of asthma.

Proprietary confidential information.
© 2012 Boehringer Ingelheim International GmbH or one or more of its affiliated companies. All rights reserved.
This document may not - in full or in part - be passed on, reproduced, published or otherwise used without prior written permission.

Missing ACQ data will be imputed according to the methods used in the validation of the respective questionnaire and will be described in detail in the TSAP.

Methods to handle any other exceptional cases will be determined finally at blineded report planning meeting (BRPM) to be held before unblinding the data so that those will apply to other trials of this type in a consistent manner.

Full details on the handling of missing data will be provided in the TSAP.

7.5 RANDOMISATION

Patients will be randomised to 2.5 µg tiotropium inhalation solution, or 5 µg tiotropium inhalation solution, or placebo with 2:2:1 allocation in blocks over the 52-week treatment period. The sponsor will arrange for the randomisation as well as packaging and labelling of study medication. Each patient will have a single randomisation number indicating the allocated treatment. Medication numbers will be assigned at a visit level. The randomisation list will be generated using a validated system involving a pseudo-random number generator and a supplied seed number, thereby ensuring that the resulting allocation to a treatment is both reproducible and nonpredictable. The seed numbers and the block size will be documented in the CTR.

Access to the randomisation codes is restricted to the Clinical Trial Support and Clinical Trial Supplies Unit at BI Pharma GmbH & Co. KG and IVRS (or IWRS) vendor. Persons directly involved in the conduct and analysis of the trial will have no access to the treatment allocation prior to the database lock.

7.6 DETERMINATION OF SAMPLE SIZE

The sample size is determined based on the notification about “sample size and exposure duration needed to assess clinical safety of new drug on its clinical stage intended for long-term treatment of non-life-threatening conditions" as described in section 7.1. According to the notification 100 patients are required for a minimum exposure of one year.

The dropout rate can be estimated approximately10% according to the study outcomes of Symbicort® and Xolair® respectively, which are accessible to the public.

Thus, based on the above regulatory guidance with the dropout rate in mind, 280 patients (112 patients per tiotropium Respimat group and 56 patients for Placebo as referenced group) are needed to evaluate the safety profile for tiotropium Respimat 2.5 µg and 5 µg.

Proprietary confidential information.
© 2012 Boehringer Ingelheim International GmbH or one or more of its affiliated companies. All rights reserved.
This document may not - in full or in part - be passed on, reproduced, published or otherwise used without prior written permission.

8. INFORMED CONSENT, DATA PROTECTION, TRIAL RECORDS

The trial will be carried out in compliance with the protocol, the principles laid down in the Declaration of Helsinki, version as of October 1996 (as long as local laws do not require to follow other versions), in accordance with the ICH Harmonised Tripartite Guideline for GCP, relevant BI SOPs, and the Japanese GCP regulations (Ministry of Health and Welfare Ordinance No. 28, March 27, 1997. Standard medical care (prophylactic, diagnostic and therapeutic procedures) remains in the responsibility of the treating physician of the patient.

The investigator should inform the sponsor immediately of any urgent safety measures taken to protect the study subjects against any immediate hazard, and also of any serious breaches of the protocol/ICH GCP and the Japanese GCP regulations (Ministry of Health and Welfare Ordinance No. 28, March 27, 1997).

The rights of the investigator, of trial site, and of the sponsor with regard to publication of the results of this trial are described in the investigator contract or trial site's contract. As a general rule, no trial results should be published prior to finalisation of the CTR.

Insurance Cover: The terms and conditions of the insurance cover are made available to the investigator and the patients via documentation in the ISF.

8.1 STUDY APPROVAL, PATIENT INFORMATION, AND INFORMED CONSENT

This trial will be initiated only after all required legal documentation has been reviewed and approved by the respective IRB/Independent Ethics Committee (IEC) and competent authority (CA) according to national and international regulations. The same applies for the implementation of changes introduced by amendments.

Prior to patient participation in the trial, written informed consent must be obtained from each patient (or the patient's legally accepted representative) according to ICH GCP and to the regulatory and legal requirements of the participating country. Each signature must be personally dated by each signatory and the informed consent and any additional patient-information form retained by the investigator as part of the trial records. A signed copy of the informed consent and any additional patient information must be given to each patient or the patient's legally accepted representative.

The investigator must give a full explanation to trial patients by using the patient information form, which is prepared avoiding the use of technical terms and expressions. In the same written informed consent it should be described to confirm if contacting patients, who prematurely discontinue the trial, after the patient's withdrawal of consent in order to obtain vital status information can be agreed. The patient is given sufficient time to consider participation in the trial. The investigator obtains written consent of the patient's own free will with the informed consent form after confirming that the patient understands the contents. The investigator must sign (or place a seal on) and date the informed consent form. If a trial collaborator has given a supplementary explanation, the trial collaborator also signs (or places a seal on) and dates the informed consent.

The patient must be informed that his/her personal trial-related data will be used by BI in accordance with the local data protection law. The level of disclosure must also be explained to the patient.

The patient must be informed that his/her medical records may be examined by authorised monitors (CML/CRA) or Clinical Quality Assurance auditors appointed by BI, by appropriate IRB/IEC members, and by inspectors from regulatory authorities.

Proprietary confidential information.
© 2012 Boehringer Ingelheim International GmbH or one or more of its affiliated companies. All rights reserved.
This document may not - in full or in part - be passed on, reproduced, published or otherwise used without prior written permission.

8.2 DATA QUALITY ASSURANCE

A quality assurance audit/inspection of this trial may be conducted by the sponsor or sponsor's designees or by IRBs/IECs or by regulatory authorities. The quality assurance auditor will have access to all medical records, the investigator's trial-related files and correspondence, and the informed consent documentation of this clinical trial.

The data management procedures to ensure the quality of the data are described in detail in the trial data management and analysis plan (TDMAP) available in the CTMF.

8.3 RECORDS

CRFs for individual patients will be provided by the sponsor, either on paper or via remote data capture. See Section 4.1.5.2 for rules about emergency code breaks. For drug accountability, refer to Section 4.1.8.

8.3.1 Source documents

Source documents provide evidence for the existence of the patient and substantiate the integrity of the data collected. Source documents are filed at the investigator's site.

Data entered in the eCRFs that are transcribed from source documents must be consistent with the source documents or the discrepancies must be explained. The investigator may need to request previous medical records or transfer records, depending on the trial; also current medical records must be available.

For eCRFs all data must be derived from source documents.

8.3.2 Direct access to source data and documents

The investigator/institution will permit trial-related monitoring, audits, IRB/IEC review and regulatory inspection, providing direct access to all related source data/documents. CRFs/eCRFs and all source documents, including progress notes and copies of laboratory and medical test results must be available at all times for review by the sponsor's clinical trial monitor, auditor and inspection by health authorities (e.g. FDA). The CRA/on site monitor and auditor may review all CRFs/eCRFs, and written informed consents. The accuracy of the data will be verified by reviewing the documents described in Section 8.3.1.

8.3.3 Storage of records

Trial sites:
The trial sites must retain the source documents and essential documents for a period defined by the Japanese GCP regulation and the trial site's contract with the sponsor.

Sponsor:
The sponsor must retain the essential documents according to the sponsor's SOPs.

Proprietary confidential information.
© 2012 Boehringer Ingelheim International GmbH or one or more of its affiliated companies. All rights reserved.
This document may not - in full or in part - be passed on, reproduced, published or otherwise used without prior written permission.

8.4 LISTEDNESS AND EXPEDITED REPORTING OF ADVERSE EVENTS

8.4.1 Listedness

To fulfil the regulatory requirements for expedited safety reporting, the sponsor evaluates whether a particular adverse event is "listed", i.e. is a known side effect of the drug or not. Therefore a unique reference document for the evaluation of listedness needs to be provided. For the 2.5 and 5 µg tiotropium bromide this is the current version of the Investigator’s Brochure (U92-0551). For the non-investigational medicinal product salbutamol, the reference document is the US-PI (Proair HFA). The current versions of these reference documents are to be provided in the ISF. No AEs are classified as listed for matching placebo, study design, or invasive procedures.

8.4.2 Expedited reporting to health authorities and IECs/IRBs

Expedited reporting of serious adverse events, e.g. suspected unexpected serious adverse reactions (SUSARs) to health authorities and IECs/IRBs, will be done according to local regulatory requirements. Further details regarding this reporting procedure are provided in the ISF.

8.5 STATEMENT OF CONFIDENTIALITY

Individual patient medical information obtained as a result of this trial is considered confidential and disclosure to third parties is prohibited with the exceptions noted below. Patient confidentiality will be ensured by using patient identification code numbers.

Treatment data may be given to the patient’s personal physician or to other appropriate medical personnel responsible for the patient’s welfare. Data generated as a result of the trial need to be available for inspection on request by the participating physicians, the sponsor’s representatives, by the IRB and the regulatory authorities.

8.6 COMPLETION OF TRIAL

When the trial is completed, the investigator should inform the head of the trial site of the completion in writing, and the head of the trial site should promptly inform the IRB and sponsor of the completion in writing.

8.7 PROTOCOL VIOLATIONS

The investigator or sub-investigator should record all CTP violations. The investigator should provide and submit the sponsor and the head of the trial site the records of violations infringing the Japanese GCP or violations to eliminate an immediate hazard to trial subjects and for other medically inevitable reasons.

8.8 COMPENSATION AVAILABLE TO THE PATIENT IN THE EVENT OF TRIAL RELATED INJURY

In the event of health injury associated with this trial, the sponsor is responsible for compensation based on the contract signed by the trial site.

Proprietary confidential information.
© 2012 Boehringer Ingelheim International GmbH or one or more of its affiliated companies. All rights reserved.
This document may not - in full or in part - be passed on, reproduced, published or otherwise used without prior written permission.

9. REFERENCES

9.1 PUBLISHED REFERENCES

[P05-12782] Miller MR, Hankinson J, Brusasco V, Burgos F, Casaburi R, Coates A, et al. Standardisation of spirometry. Eur Respir J 2005; 26 (2):319-338.

[P10-03196] Global Initiative for Asthma (GINA). Global strategy for asthma management and prevention (updated 2009). website: ginasthma.org 2009; .

[R94-1408] Quanjer PH, Tammeling GJ, Cotes JE, Pedersen OF, Peslin R, Yernault JC Lung volumes and forced ventilatory flows.Report Working Party Standardization of Lung Function Tests, European Community for Steel and Coal.Official Statement of the European Respiratory Society. Eur Respir J 1993;6(Suppl 16):5-40.

[R00-1157] Juniper EF, O'Byrne PM, Guyatt GH, Ferrie PJ, King DR Development and validation of a questionnaire to measure asthma control. Eur Respir J 1999;14:902-907.

[R08-5197] Lange P, Nyboe J, Appleyard M, Jensen G, Schnohr P Relationship of the type of tobacco and inhalation pattern to pulmonary and total mortality. Eur Respir J 1992;5:1111-1117.

[R09-1589] Juniper E Asthma Control Questionnaire: background, administration and analysis. website: qoltech.co.uk ; Bosham: QOL Technologies 2005.

[R10-4989] Asthma prevention and management guideline 2009, Japan in Japanese. Japanese Society of Allergology 2009.

9.2 UNPUBLISHED REFERENCES

[U92-0551] Investigator´s Brochure, Tiotropium Bromide, Ba 679 BR (tiotropium bromide inhalation powder and tiotropium bromide inhalation solution). Project No. 205.P1 to 205. P5. Version 17. 05 February 2010.

[U96-0240] Towse LJ, Dewberry HM. A double-blind, placebo-controlled, crossover study to determine the effect of inhaled Ba 679 BR (tiotropium) on methacholine responsiveness in patients with mild asthma. BI Trial No. 205.121. 03 June 1996.

[U98-3174] Koker P, Souhrada JF, Terlizzi E, Wang Q, Vogt K. The effect of twenty-one day dosing of tiotropium on bronchomotor tone in patients with moderate to severe asthma (a randomized, double-blind, placebo-controlled, parallel study). BI Trial No. 205.201. 17 August 1998.

[U98-3274] Koker P, Souhrada JF, Terlizzi E, Wang Q, Reilly K. The effect of tiotropium in patients with nocturnal asthma (a randomized, double-blind, double-dummy, placebo and active-controlled, parallel study). BI Trial No. 205.202. 09 November 1998.

Proprietary confidential information.
© 2012 Boehringer Ingelheim International GmbH or one or more of its affiliated companies. All rights reserved.
This document may not - in full or in part - be passed on, reproduced, published or otherwise used without prior written permission.

[U99-1019] Freund E, Beckers B. The protective effect and safety of 36 µg inhaled tiotropium (Ba 679) against exercise-induced bronchoconstriction in patients with bronchial asthma (double-blind, randomised, placebo-controlled, parallel study). BI Trial No. 205.203. 20 October 1998.

[U02-1222] Vitry F, Lafferre M. Evaluation of local tolerability of an acidic (pH=2.7) solution for inhalation administered via the RESPIMAT® device in 32 asthmatic adults. A single-dose (4 puffs), cross-over randomized study. BI Trial No. 205.248. 22 January 2002.

[U04-2041] Cornelissen PJG, Van Noord JA, Mueller A, Platz J. A Randomised, Double-Blind, Double-Dummy, Placebo-Controlled, Crossover Efficacy and Safety Comparison of 4-Week Treatment Periods of Two Doses [5 µg (2 actuations of 2.5 µg) and 10 µg (2 actuations of 5 µg] of Tiotropium Inhalation Solution Delivered by the Respimat Inhaler, Tiotropium Inhalation Powder Capsule (18 µg) delivered by the HandiHaler in Patients with Chronic Obstructive Pulmonary Disease (COPD). 26 November 2004.

[U05-1949] Cornelissen PJG, Mueller A, Platz J, Fogarty C. A Randomised, Double-Blind, Double-Dummy, Placebo-Controlled, Crossover Efficacy and Safety Comparison of 4-Week Treatment Periods of Two Doses [5 µg (2 actuations of 2.5 µg) and 10 µg (2 actuations of 5 µg)] of Tiotropium Inhalation Solution Delivered by the Respimat Inhaler, Tiotropium Inhalation Powder Capsule (18 µg) Delivered by the HandiHaler in Patients with Chronic Obstructive Pulmonary Disease (COPD). 01 August 2005.

[U07-3262] Fujimoto T, Kagimura T, Hata A, Tatami S. A randomised, double-blind, double-dummy, crossover efficacy and safety comparison of 4-week treatment periods of Ba 679 BR Respimat 5 µg and tiotropium inhalation capsule 18 µg in patients with COPD. 03August 2007.

[U08-2081] Sigmund R, Jordan D, Kerstjens HAM, Gahlemann M. A Randomised, Double-Blind, Placebo-Controlled, Crossover Efficacy and Safety Evaluation of 8-Week Treatment Periods of Two Doses [5 µg (2 actuations of 2.5 µg) and 10 µg (2 actuations of 5 µg)] of Tiotropium Inhalation Solution Delivered by the Respimat Inhaler as Add-on Therapy in Patients with severe persistent Asthma. BI Trial No. 205.341. 22 October 2008.

[U09-1701] Schmidt P, Kornmann O, Pivovarova A, Frampton H. A 16-week randomised, placebo-controlled, double-blind, doubledummy, parallel-group study comparing the efficacy and safety of tiotropium inhalation solution delivered by the Respimat® inhaler (2 puffs of 2.5 µg once daily) with that of salmeterol from the hydrofluoroalkane metered dose inhaler (2 puffs of 25 µg twice daily) in moderate persistent asthma patients homozygous for B16-Arg/Arg. BI Trial No. 205.342. 14 August 2009.

Proprietary confidential information.
© 2012 Boehringer Ingelheim International GmbH or one or more of its affiliated companies. All rights reserved.
This document may not - in full or in part - be passed on, reproduced, published or otherwise used without prior written permission.

10. APPENDICES

10.1 INSTRUCTIONS FOR THE USE OF THE RESPIMAT INHALER

Instructions for Use

Respimat inhaler

How to use your Respimat inhaler

This leaflet explains how to use and care for your Respimat inhaler. Please read and carefully follow these instructions.

The Respimat inhaler releases medication slowly and gently, making it easy to inhale it into your lungs.

The Respimat inhaler enables you to inhale the medicine contained in a cartridge. You will need to use this inhaler only ONCE A DAY. Each time you use it take 2 actuations. In the box you will find the Respimat inhaler and the Respimat cartridge. Before the Respimat inhaler is used for the first time, the cartridge provided must be inserted.

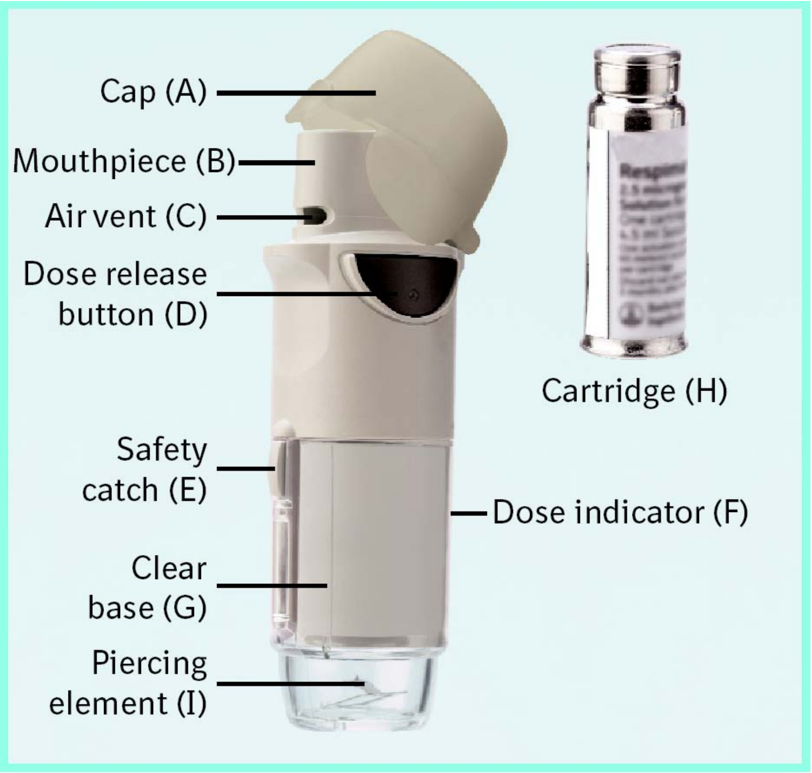

Respimat inhaler and the Respimat cartridge

Proprietary confidential information.
© 2012 Boehringer Ingelheim International GmbH or one or more of its affiliated companies. All rights reserved.
This document may not - in full or in part - be passed on, reproduced, published or otherwise used without prior written permission.

Inserting the cartridge and preparation for use

The following steps 1-6 are necessary before the first-time use:

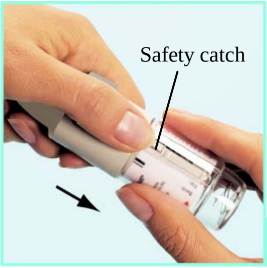 1	1) With the grey cap closed, press the safety catch (E) and pull off the clear base (G).
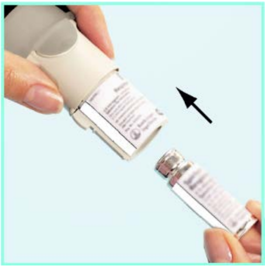 2a<br>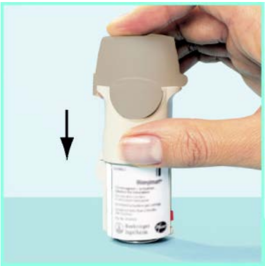 2b	2) Take the cartridge (H) out of the box. Push the narrow end of the cartridge into the inhaler until it clicks into place (2a). The cartridge should be pushed gently against a firm surface to ensure that it has gone all the way in (2b).<br><br>Do not remove the cartridge once it has been inserted into the inhaler.
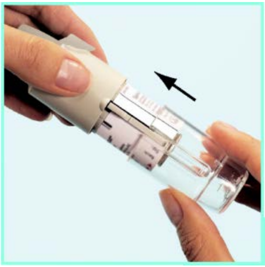 3	3) Replace the clear base (G).<br>Do not remove the clear base again.

Proprietary confidential information.
© 2012 Boehringer Ingelheim International GmbH or one or more of its affiliated companies. All rights reserved.
This document may not - in full or in part - be passed on, reproduced, published or otherwise used without prior written permission.

To prepare the Respimat inhaler for the first-time use

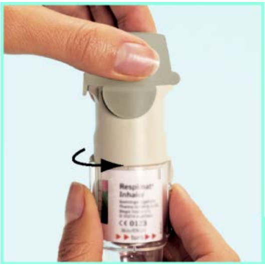 4	4) Hold Respimat inhaler upright, with the grey cap (A) closed. Turn the clear base (G) in the direction of the red arrows on the label until it clicks (half a turn).
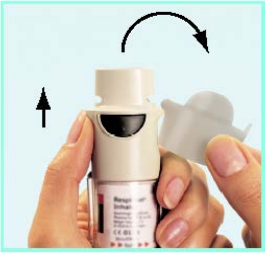 5	5) Open the grey cap (A) until it snaps fully open.
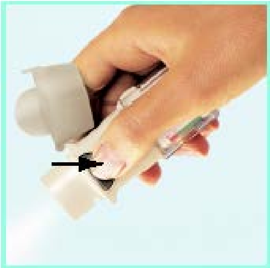 6	6) Point the Respimat inhaler towards the ground.<br>Press the dose release button (D). Close the grey cap (A).<br><br>Repeat steps 4, 5 and 6 until a cloud is visible.<br><br>Then repeat steps 4, 5 and 6 three more times to ensure the inhaler is prepared for use.<br><br>Your Respimat inhaler is now ready to use.<br><br>These steps will not affect the number of doses available. After preparation your Respimat inhaler will be able to deliver 60 actuations.

Proprietary confidential information.
© 2012 Boehringer Ingelheim International GmbH or one or more of its affiliated companies. All rights reserved.
This document may not - in full or in part - be passed on, reproduced, published or otherwise used without prior written permission.

Using the Respimat inhaler

You will need to use this inhaler only ONCE A DAY.

Each time you use it take 2 actuations.

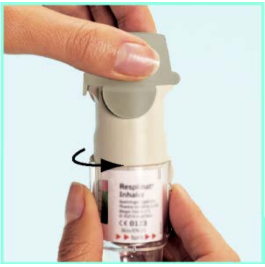 I	I) Hold Respimat inhaler upright, with the grey cap (A) closed, to avoid accidental release of dose. Turn the clear base (G) in the direction of the red arrows on the label until it clicks (half a turn).
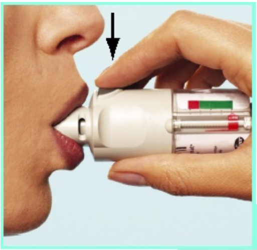 II	II) Open the grey cap (A) until it snaps fully open. Breathe out slowly and fully, and then close your lips around the end of the mouthpiece without covering the air vents (C). Point your Respimat inhaler to the back of your throat. While taking in a slow, deep breath through your mouth, press the dose release button (D) and continue to breathe in slowly for as long as you can. Hold your breath for 10 seconds or for as long as comfortable.<br>III) Repeat steps I and II one more time so that you get the full dose.<br><br>You will need to use this inhaler only ONCE A DAY.<br><br>Close the grey cap until you use your Respimat inhaler next time.<br><br>If the Respimat inhaler has not been used for more than 3 days release one actuation towards the ground. If the Respimat inhaler has not been used for more than 21 days repeat steps 4 to 6 until a cloud is visible. Then repeat steps 4 to 6 three more times.

Proprietary confidential information.
© 2012 Boehringer Ingelheim International GmbH or one or more of its affiliated companies. All rights reserved.
This document may not - in full or in part - be passed on, reproduced, published or otherwise used without prior written permission.

When to get a new Respimat inhaler

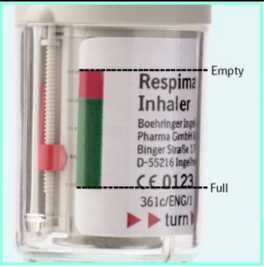	The Respimat inhaler contains 60 actuations (30 doses). The dose indicator shows approximately how many doses are left. When the pointer enters the red area of the scale, there is, approximately, medication for 15 actuations (7 days) left.<br><br>Once the dose indicator has reached the end of the red scale (i.e., all 60 doses have been used), the Respimat inhaler is empty and locks automatically. At this point, the base cannot be turned any further.

What if...

What if...	Reason	What to do
I can't turn the base easily.	a) The Respimat inhaler is already prepared and ready to use.<br><br>b) The Respimat inhaler is locked after 60 actuations (30 doses).	a) The Respimat inhaler can be used as it is.<br><br>b) Prepare and use your new Respimat inhaler.
I can't press the dose release button.	The clear base has not been turned.	Turn the clear base until it clicks. (half a turn)
The clear base springs back after I have turned it.	The clear base was not turned far enough.	Prepare the Respimat inhaler for use by turning the clear base until it clicks. (half a turn)
I can turn the clear base past the point where it clicks.	Either the dose release button has been pressed, or the clear base has been turned too far.	With the grey cap closed, turn the base until it clicks. (half a turn)

Proprietary confidential information.
© 2012 Boehringer Ingelheim International GmbH or one or more of its affiliated companies. All rights reserved.
This document may not - in full or in part - be passed on, reproduced, published or otherwise used without prior written permission.

How to care for your inhaler

Clean the mouthpiece including the metal part inside the mouthpiece with a damp cloth or tissue only, at least once a week.

Any minor discoloration in the mouthpiece does not affect the performance of your Respimat inhaler.

If necessary, wipe the outside of your Respimat inhaler with a damp cloth.

Further information

The Respimat inhaler must not be disassembled after inserting the cartridge and replacing the clear base.

Do not touch the piercing element inside the base.

Keep out of the reach and sight of children.

Do not freeze.

Boehringer Ingelheim Pharma GmbH & Co. KG

D - 55216 Ingelheim

Germany

CE 0123

HI-Master-Version-Respimat-20080831

PLEASE ALWAYS ENTER THE DATE OF FIRST PRIMING

ON THE MEDICATION LABEL!

Proprietary confidential information.
© 2012 Boehringer Ingelheim International GmbH or one or more of its affiliated companies. All rights reserved.
This document may not - in full or in part - be passed on, reproduced, published or otherwise used without prior written permission.

10.2 INSTRUCTIONS FOR RETURN OF MALFUNCTIONING RESPIMAT INHALERS

Respimat inhalers, with the used cartridge in situ, that appeared to malfunction, will be returned to the Boehringer Ingelheim OPU responsible for packaging and labeling as soon as possible. The name, address and contact person are listed below:

Mr. Friedemann Kaufmann
Boehringer Ingelheim Pharma GmbH & Co. KG
Business Unit Development
Dpt. Quality & Records Management
55216 Ingelheim am Rhein
Germany

The following information should be included when the inhaler is returned:

a) Medication number

b) Visit number

c) Date of malfunction

d) Description of malfunction and cause of malfunction (if known)

e) Person identifying malfunction

f) Malfunctioned after amount of days or weeks of treatment

g) BI personnel contacted and date contacted

h) Date shipped to Boehringer Ingelheim

i) Trial number/country

j) Investigator’s name/center number

l) Date of return to the investigator

The original of the Product/Device Complaint Form should be included with the returned inhaler. In parallel, a scanned copy of the form should be send to the responsible CTSU coordinator of the trial via email. A copy of the form should be filed with the Drug Dispensing Log.

All inhalers and cartridges should be wrapped in bubble wrap or a similar packing material, placed in a secure shipping box (not a packing envelope) and shipped by overnight express.

Any questions regarding shipping and handling should be directed to the CML

Proprietary confidential information.
© 2012 Boehringer Ingelheim International GmbH or one or more of its affiliated companies. All rights reserved.
This document may not - in full or in part - be passed on, reproduced, published or otherwise used without prior written permission.

10.3 ADDITIONAL INFORMATION REGARDING IN/EX CRITERIA

Classification of medium ICS doses

Table 10.3: 1 Definition of medium daily doses of ICS adapted from GINA 2009 (P10-03196)

Drug	*Medium daily Dose (µg)*
Beclomethasone dipropionate	≥500 and ≤1000
Budesonide	≥400 and ≤800
Ciclesonide	≥160 and ≤320
Fluticasone	≥250 and ≤500
Mometasone furoate	≥400 and ≤800

As CFC preparations are taken from the market, medication inserts for HFA preparations should be carefully reviewed by the investigator for the equivalent correct dosage. Regarding specific requirements in Japan please refer to the ISF for a detailed list.

Reversibility testing (P05-12782)

At the screening visit (Visit 1) following the completion of 3 acceptable pre-bronchodilator forced expiratory manoeuvres, salbutamol HFA MDI will be administered to each patient in order to document the degree of reversibility. Immediately after (within 10 min) pre-bronchodilator forced expiratory manoeuvres and after a gentle and incomplete expiration, a dose of 100 µg of salbutamol HFA MDI is inhaled in one breath to total lung capacity (TLC). The breath is then held for 5–10 seconds before the subject exhales. Four separate doses (total dose 400 µg) are delivered at approximately 30-second intervals (this dose ensures that the response is high on the salbutamol HFA MDI dose–response curve). 3 additional, acceptable post-bronchodilator forced expiratory manoeuvre tests are recorded ≥10 min and up to 30 min later after the last dose of salbutamol HFA MDI is inhaled.

Calculation of predicted normal values according to ECSC (R94-1408)

For height measured in meters
Males: FEV_1 predicted (L) = 4.30 x [height (m)] - 0.029 x [age (yrs)] - 2.49
Females: FEV_1 predicted (L) = 3.95 x [height (m)] - 0.025 x [age (yrs)] - 2.60

Patients with ages 18-25 will have predicted FEV_1 calculated with age 25.

The race correction factors in Table 10.3: 2 will apply.

Proprietary confidential information.
© 2012 Boehringer Ingelheim International GmbH or one or more of its affiliated companies. All rights reserved.
This document may not - in full or in part - be passed on, reproduced, published or otherwise used without prior written permission.

Table 10.3: 2 Race correction factors

Races/Ethnicities	*Correction factor FEV_1*	*Correction factor FVC*
Caucasian	1.0	1.0
Orientals Hong Kong Chinese	1.0	1.0
Orientals Japanese Americans	0.89	1.0
Polynesians	0.9	0.9
North Indians & Pakistanis	0.9	0.9
South Indians	0.87	0.87
African Descent	0.87	0.87
Other (Japanese)	1.0	1.0

Calculation of variation of absolute FEV_1 values

The value of Visit 1 is regarded as 100% and the following formula applies:

$$FEV_1 \text{ variation (\%)} = \frac{FEV_1 \text{ pre-dose at Visit 2 (L)}}{FEV_1 \text{ pre-bronchodilator at Visit 1 (L)}} \times 100 - 100$$

Calculation of number of pack years

$$\text{Pack years} = \frac{\text{Number of cigarettes/day}}{20} \times \text{years of smoking}$$

The following equivalents for the tobacco content should be used for smokers other than cigarettes smokers (R08-5197):

- One plain or filter cigarette = 1 gram of tobacco
- One cigar = 5 grams of tobacco
- One cheroot or cigarillo = 3 grams of tobacco
- One gram of pipe tobacco = 1 gram of tobacco

Calculation of pack years based on tobacco contents:

$$\text{Pack years} = \frac{\text{Number of gram/day}}{20} \times \text{years of smoking}$$

Proprietary confidential information.
© 2012 Boehringer Ingelheim International GmbH or one or more of its affiliated companies. All rights reserved.
This document may not - in full or in part - be passed on, reproduced, published or otherwise used without prior written permission.

10.4 ASTHMA CONTROL QUESTIONNAIRE (ACQ)

ASTHMA CONTROL QUESTIONNAIRE

© 1997
QOL TECHNOLOGIES Ltd.

For further information:

Elizabeth Juniper, MCSP, MSc
Professor
20 Marcuse Fields,
Bosham,
West Sussex,
PO18 8NA. UK
Telephone: + 44 (0) 1243 572124
Fax: + 44 (0) 1243 573680
E-mail: juniper@qoltech.co.uk
Web: www.qoltech.co.uk

© The Asthma Control Questionnaire is copyrighted. It may not be altered, sold (paper or electronic), translated or adapted for another medium without the permission of Elizabeth Juniper.

December 2002

Proprietary confidential information.
© 2012 Boehringer Ingelheim International GmbH or one or more of its affiliated companies. All rights reserved.
This document may not - in full or in part - be passed on, reproduced, published or otherwise used without prior written permission.

ASTHMA CONTROL QUESTIONNAIRE© PATIENT ID:____________________________

DATE:________________________________

Page 1 of 2

Please answer questions 1 - 6.

Circle the number of the response that best describes how you have been during the past week.

1. On average, during the past week, how often were you **woken by your asthma** during the night?
   - 0 Never
   - 1 Hardly ever
   - 2 A few times
   - 3 Several times
   - 4 Many times
   - 5 A great many times
   - 6 Unable to sleep because of asthma

2. On average, during the past week, how **bad were your asthma symptoms when you woke up** in the morning?
   - 0 No symptoms
   - 1 Very mild symptoms
   - 2 Mild symptoms
   - 3 Moderate symptoms
   - 4 Quite severe symptoms
   - 5 Severe symptoms
   - 6 Very severe symptoms

3. In general, during the past week, how **limited were you in your activities** because of your asthma?
   - 0 Not limited at all
   - 1 Very slightly limited
   - 2 Slightly limited
   - 3 Moderately limited
   - 4 Very limited
   - 5 Extremely limited
   - 6 Totally limited

4. In general, during the past week, how much **shortness of breath** did you experience because of your asthma?
   - 0 None
   - 1 A very little
   - 2 A little
   - 3 A moderate amount
   - 4 Quite a lot
   - 5 A great deal
   - 6 A very great deal

Proprietary confidential information.
© 2012 Boehringer Ingelheim International GmbH or one or more of its affiliated companies. All rights reserved.
This document may not - in full or in part - be passed on, reproduced, published or otherwise used without prior written permission.

ASTHMA CONTROL QUESTIONNAIRE© PATIENT ID:______________________________

DATE:__________________________________

Page 2 of 2

5. In general, during the past week, how much of the time did you **wheeze?**
   - 0 Not at all
   - 1 Hardly any of the time
   - 2 A little of the time
   - 3 A moderate amount of the time
   - 4 A lot of the time
   - 5 Most of the time
   - 6 All the time

6. On average, during the past week, how many **puffs/inhalations of short-acting bronchodilator** (e.g. Ventolin/Bricanyl) have you used each day? *(If you are not sure how to answer this question, please ask for help)*
   - 0 None
   - 1 1 - 2 puffs/inhalations most days
   - 2 3 - 4 puffs/inhalations most days
   - 3 5 - 8 puffs/inhalations most days
   - 4 9 - 12 puffs/inhalations most days
   - 5 13 - 16 puffs/inhalations most days
   - 6 More than 16 puffs/inhalations most days

To be completed by a member of the clinic staff

7. FEV_1pre-bronchodilator: ....

   FEV_1predicted:.....................................

   FEV_1%predicted:.................................
   (Record actual values on the dotted lines and score the FEV_1 % predicted in the next column)
   - 0 > 95% predicted
   - 1 95 - 90%
   - 2 89 - 80%
   - 3 79 - 70%
   - 4 69 - 60%
   - 5 59 - 50%
   - 6 < 50% predicted

Proprietary confidential information.
© 2012 Boehringer Ingelheim International GmbH or one or more of its affiliated companies. All rights reserved.
This document may not - in full or in part - be passed on, reproduced, published or otherwise used without prior written permission.

10.5 PAPER PATIENT DIARY CARD

Boehringer Ingelheim

BI Trial No. 205.464

Tiotropium /TINA

Patient's documentation of Asthma worsening

Patient No.:

Asthma symptoms new or increased?							Actions due to new or increased asthma symptoms			
Start date (dd mon yy)	End date (dd mon yy)	Cough	Wheezing	Shortness of breath	Chest tightness	Others	Start date (dd mon yy)	End date (dd mon yy)	Change in asthma medication (Tradename, Route of administration)	Need for any medical care (physician, hospital) Or Lost working days
01-JUL-10	02-JUL-10	X		X	X		02-JUL-10	05-JUL-10		

Proprietary confidential information.
© 2012 Boehringer Ingelheim International GmbH or one or more of its affiliated companies. All rights reserved.
This document may not - in full or in part - be passed on, reproduced, published or otherwise used without prior written permission.

10.6 AM3 PATIENT INSTRUCTION CARD

Please refer to ISF.

Proprietary confidential information.
© 2012 Boehringer Ingelheim International GmbH or one or more of its affiliated companies. All rights reserved.
This document may not - in full or in part - be passed on, reproduced, published or otherwise used without prior written permission.

10.7 DEFINITION ASTHMA EXACERBATION

Asthma exacerbation

For the purposes of this trial, an asthma exacerbation in general is defined by the sponsor as

- an episode of progressive increase in one or more asthma symptoms, like shortness of breath, cough, wheezing, or chest tightness, or some combination of these symptoms. Respiratory distress is common. The symptoms should be outside the patient´s usual range of day-to-day asthma and should last for at least two consecutive days.

and/or

- a decrease of patient´s best morning PEF of ≥30% from the patient´s mean morning PEF for at least two consecutive days. Relevant PEF deteriorations are marked on the AM3 data reports downloaded at each visit.

  During the screening period, patient's mean morning PEF is defined as the mean value of all best morning PEF values obtained during the first 7 days after Visit 1.

  During the treatment period, patient's mean morning PEF is defined as the mean value of all best morning PEF values obtained during the complete screening period including the morning of Visit 2.

<u>Severe asthma exacerbation</u>

Severe asthma exacerbations are defined by the sponsor as a subgroup from all asthma exacerbations according to sponsor´s definition given above that require an initiation of treatment with systemic (including oral) corticosteroids for at least 3 days.

PEF decreases marked on AM3 report

A asymptomatic PEF-decrease as described above is considered an asthma exacerbation per protocol, regardless of being accompanied by asthma symptoms, need for additional asthma medication or if considered medically relevant or not. At every AM3 download, the report includes an alert section summarizing all relevant PEF-decreases that occurred since the last visit. The investigator needs to discuss the report with the patient and decide which alerts are valid (explained by decreased lung function) and which are not valid (e.g. explained by non-compliance as for instance device was used by other person than patient or PEF measurement done incorrectly).

For each valid alert, the investigator needs to document this finding as adverse event in the eCRF. If symptomatic, examples of AE verbatims would be asthma aggravation, asthma exacerbation or bronchitis. If asymptomatic, the AE verbatim would be ´asymptomatic peak expiratory flow

Proprietary confidential information.
© 2012 Boehringer Ingelheim International GmbH or one or more of its affiliated companies. All rights reserved.
This document may not - in full or in part - be passed on, reproduced, published or otherwise used without prior written permission.

decrease´ if no symptoms were reported. In addition, the Asthma exacerbation verification page in the eCRF needs to be entered.

Note: if a respiratory tract infection without asthma worsening was the reason of PEF decrease (e.g. bronchitis), then this would only be documented as AE, not as asthma exacerbation per protocol.

For each non-valid alert, the investigator needs to document the rationale on the AM3-report.

Proprietary confidential information.
© 2012 Boehringer Ingelheim International GmbH or one or more of its affiliated companies. All rights reserved.
This document may not - in full or in part - be passed on, reproduced, published or otherwise used without prior written permission.

10.8 CLINICAL LAB PARAMETERS

Laboratory specimens need to be taken prior to the salbutamol HFA MDI dosing. Lab parameters will be analysed by the central laboratory. Lab samples may be stored overnight. The local lab at the sites should be contacted to discuss the required overnight storage conditions (fridge or room temperature).

The haematological parameters will include the following:

- Haemoglobin
- Haematocrit
- Absolute and relative eosinophil count (to be recorded on eCRF)
- Red blood cell count
- White blood cell count (to be recorded on eCRF) including differential test (neutrophils, lymphocytes, monocytes, eosinophils, basophils)
- Platelet count

The blood chemistry parameters will include the following:

- Total serum IgE (to be recorded on eCRF)
- LDH
- γ-GT
- SGOT (AST)
- SGPT (ALT)
- Calcium
- Inorganic phosphorus
- Creatinine (to be recorded on eCRF)
- Potassium
- Sodium
- Chloride

Proprietary confidential information.
© 2012 Boehringer Ingelheim International GmbH or one or more of its affiliated companies. All rights reserved.
This document may not - in full or in part - be passed on, reproduced, published or otherwise used without prior written permission.

11. DESCRIPTION OF GLOBAL AMENDMENT

**Number of global amendment**		1
**Date of CTP revision**		10 February 2011
**EudraCT number**		-
**BI Trial number**		205.464
**BI Investigational Product**		Tiotropium inhalation solution - Respimat Inhaler (Ba 679 BR Respimat)
**Title of protocol**		A Phase III randomised, double-blind, placebo-controlled, parallel-group trial to evaluate safety and efficacy of tiotropium inhalation solution delivered via Respimat inhaler (2.5 and 5 µg once daily) compared with placebo over 52 weeks in patients with moderate to severe persistent asthma
**To be implemented only after approval of the IRB**		☒
**To be implemented immediately in order to eliminate hazard – IRB to be notified of change with request for approval**		☐
**Can be implemented without IRB approval as changes involve logistical or administrative aspects only**		☐
**Section to be changed**		10.3 Additional information regarding in/ex criteria
**Description of change**		To clarify race correction factor of Japanese
**Rationale for change**		According to a change of handling race correction factor of Japanese which was reconsidered by the sponsor, it was decided to describe it in the protocol that "Others" should be used for Japanese's data.

Proprietary confidential information.
© 2012 Boehringer Ingelheim International GmbH or one or more of its affiliated companies. All rights reserved.
This document may not - in full or in part - be passed on, reproduced, published or otherwise used without prior written permission.

**Number of global amendment**		2
**Date of CTP revision**		5 January 2012
**EudraCT number**		-
**BI Trial number**		205.464
**BI Investigational Product**		Tiotropium inhalation solution - Respimat Inhaler (Ba 679 BR Respimat)
**Title of protocol**		A Phase III randomised, double-blind, placebo-controlled, parallel-group trial to evaluate safety and efficacy of tiotropium inhalation solution delivered via Respimat inhaler (2.5 and 5 µg once daily) compared with placebo over 52 weeks in patients with moderate to severe persistent asthma
**To be implemented only after approval of the IRB**		☒
**To be implemented immediately in order to eliminate hazard –<br>IRB to be notified of change with request for approval**		☐
**Can be implemented without IRB approval as changes involve logistical or administrative aspects only**		☐
**Section to be changed**	I)<br>II)<br>III)<br>IV)<br>V)	*Flowchart and Section 6.2.2*<br>*Abbreviations, Section 5.2.3 and Section 10.8.*<br>*Section 3.3.2*<br>*Section 5.2.2.1*<br>*Section 5.2.2.2*
**Description of change**	I)<br>II)<br>III)<br>IV)<br>V)	*Administrative additions and clarification.*<br>*Administrative additions.*<br>*Clarification.*<br>*Administrative change and clarification.*<br>*Administrative change.*
**Rationale for change**	I)<br>II)<br>III)<br>IV)<br><br><br>V)	*Administrative additions and clarification.*<br>*Administrative additions.*<br>*Clarification spirometry criteria.*<br>*Administrative change regarding reporting of significant events.*<br>*Clarification of (S)AE reporting.*<br>*Administrative change regarding reporting of always serious AEs.*

Proprietary confidential information.
© 2012 Boehringer Ingelheim International GmbH or one or more of its affiliated companies. All rights reserved.
This document may not - in full or in part - be passed on, reproduced, published or otherwise used without prior written permission.

**Number of global amendment**		3
**Date of CTP revision**		01 Jun 2012
**EudraCT number**		-
**BI Trial number**		205.464
**BI Investigational Product**		Tiotropium inhalation solution - Respimat Inhaler (Ba 679 BR Respimat)
**Title of protocol**		A Phase III randomised, double-blind, placebo-controlled, parallel-group trial to evaluate safety and efficacy of tiotropium inhalation solution delivered via Respimat inhaler (2.5 and 5 µg once daily) compared with placebo over 52 weeks in patients with moderate to severe persistent asthma
**To be implemented only after approval of the IRB**		☒
**To be implemented immediately in order to eliminate hazard –<br>IRB to be notified of change with request for approval**		☐
**Can be implemented without IRB approval as changes involve logistical or administrative aspects only**		☐
**Section to be changed**	I)<br>II)	*Front page,Clinical Trial Protocol Synopsis*<br>*Section 7.3.3*
**Description of change**	I)<br>II)	*Change of Coordinating Investigator's Institution*<br>*Change of safety evaluation period for safety analyses*
**Rationale for change**	I)<br>II)	*Due to transfer*<br>*Adapt for project standard*

Proprietary confidential information.
© 2012 Boehringer Ingelheim International GmbH or one or more of its affiliated companies. All rights reserved.
This document may not - in full or in part - be passed on, reproduced, published or otherwise used without prior written permission.
