# Supplementary material for: Long-Term Once-Daily Tiotropium Respimat® Is Well Tolerated and Maintains Efficacy over 52 Weeks in Patients with Symptomatic Asthma in Japan: A Randomised, Placebo-Controlled Study
Source: PLoS One. 2015 Apr 20;10(4):e0124109. doi: 10.1371/journal.pone.0124109 (PMC4404354; doi:10.1371/journal.pone.0124109)
Supplement: S1 Table — (DOCX) [file pone.0124109.s005.docx]

**Supporting Information**

**Table S1. Serious adverse events in each treatment group**

| **Preferred term** | **n** | **Intensity** | **Treatment-related?** | **Action required** |
| --- | --- | --- | --- | --- |
| **Tiotropium Respimat^®^ 5 µg** |  |  |  |  |
| Asthma | 1 | Moderate | No | None |
| Intervertebral disc protrusion | 1 | Moderate | No | None |
| Ovarian cyst | 1 | Moderate | No | None |
| Subileus | 1 | Moderate | No | None |
| **Tiotropium Respimat^®^ 2.5 µg** |  |  |  |  |
| Cellulitis | 1 | Severe | No | None |
| Influenza | 1 | Moderate | No | None |
| Oral papilloma | 1 | Mild | No | None |
| Pain in extremity | 1 | Moderate | No | None |
| **Placebo** |  |  |  |  |
| Aortic dissection | 1 | Severe | No | Discontinued |
| Asthma | 1 | Severe | Yes | None |
| Cyst | 1 | Mild | No | None |
| Decreased appetite | 1 | Moderate | No | None |
| Deep vein thrombosis | 1 | Mild | No | None |
| Diverticulitis | 1 | Mild | No | None |
| Loss of consciousness | 1 | Severe | No | None |
| Mesenteric haemorrhage^a^ | 1 | Moderate | No | None |
| Pneumonia bacterial | 1 | Mild | No | None |
| Rib fracture^a^ | 1 | Moderate | No | None |
| Sternal fracture^a^ | 1 | Moderate | No | None |

^a^Occurred in the same patient.
